# Nucleotide Metabolism in Health and Disease

**DOI:** 10.1002/mco2.70900

**Published:** 2026-08-12

**Authors:** Xiaoying Zhao, Yiyu Zeng, Yike Xu, Shu Rao, Mouna Khouchani, Hassna Soummane, Jingyi Li, Rui Liu

**Affiliations:** ^1^ Irradiation Preservation and Effect Key Laboratory of Sichuan Province. The Second Affiliated Hospital of Chengdu Medical College, Nuclear Industry 416 Hospital Chengdu Sichuan China; ^2^ Sichuan Clinical Research Center for Radiation and Therapy The Second Affiliated Hospital of Chengdu Medical College, Nuclear Industry 416 Hospital Chengdu China; ^3^ State Key Laboratory of Oral Diseases& National Center for Stomatology & National Clinical Research Center for Oral Diseases West China Hospital of Stomatology Sichuan University Chengdu China; ^4^ City University of Hong Kong Kowloon Hong Kong China; ^5^ Mohammed VI University Hospital, Cadi Ayyad University Marrakech Morocco

**Keywords:** DNA repair, immune diseases, neurodegenerative diseases, nucleotide metabolism, tumor

## Abstract

Nucleotide metabolism is a fundamental biochemical process, providing the building blocks for DNA and RNA synthesis while driving critical cellular functions like energy transfer, signal transduction, and coenzyme synthesis. The precise regulation of nucleotide de novo synthesis, salvage, and degradation pathways is imperative for maintaining genomic stability and tissue homeostasis. Aberrations in these networks are increasingly recognized as primary drivers in various pathologies; notably, cancer cells exploit metabolic–functional reprogramming to enable rapid proliferation and therapeutic resistance. Despite the growing recognition of these metabolic vulnerabilities, an integrated understanding of how specific enzymatic dysregulations translate into diverse disease phenotypes remains fragmented. This review systematically synthesizes the physiological regulatory mechanisms governing nucleotide metabolism and describes the molecular underpinnings of its dysregulation across malignant, immune, and neurodegenerative disorders. We critically examine how specific metabolic enzymes are exploited to drive pathogenesis and highlight the complex interplay between nucleotide metabolites and inflammatory or oncogenic signaling cascades. Furthermore, we evaluate recent clinical innovations in precision medicine, focusing on novel inhibitors targeting key metabolic nodes. By mapping these intricate metabolic networks, this article provides a crucial basis for overcoming current therapeutic challenges, offering profound insights to accelerate the development of next‐generation targeted therapies.

## Graphical abstract

Nucleotide metabolism, including de novo synthesis, salvage pathways, and catabolism, when dysregulated contributes to cancer, immune disorders, metabolic and urological diseases, and radiation injury. Metabolites such as adenosine, cGAMP, NAD, and cAMP and enzymes like RNR are promising therapeutic targets and biomarkers.

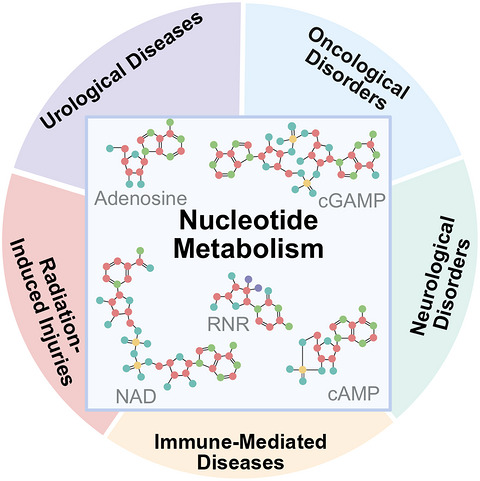

## 1 Introduction

Nucleotides are the basic units that make up nucleic acids (DNA and RNA) and also serve as important components of cellular energy currency, signaling molecules, and various coenzymes [1]. The dynamic balance of nucleotides in cells is mainly maintained through three pathways, including de novo synthesis pathway—using small molecule precursors such as amino acids and one carbon units to gradually synthesize, salvage synthesis pathway—reusing bases/nucleosides produced by nucleic acid degradation, and catabolic pathway—degrading and eliminating excess or damaged nucleotides. These pathways are subject to strict allosteric regulation, transcriptional regulation, and substrate availability control to ensure the stability of nucleotide libraries under different physiological states [2].

The precise balance of nucleotide metabolism is the foundation for maintaining normal cellular function. For example, tissues with active proliferation are highly dependent on efficient nucleotide synthesis to support DNA replication and cell division. Terminally differentiated tissues (such as nerves and muscles) rely more on remedial pathways to conserve energy and maintain nucleotide pools. The activation and differentiation of immune cells require metabolic reprogramming, in which upregulation of nucleotide synthesis is key to supporting their clonal expansion and effector functions. In addition, in the nervous system, ATP serves as the main energy source for neurotransmitter release and ion pump operation, while cAMP/cGMP participates in signaling pathways such as learning and memory. Cancer originates from cellular metabolic disorders, and changes in nucleotide metabolism are crucial for DNA replication, repair, and genomic stability. The dynamic metabolic demands of cancer cells require stimulation of de novo nucleotide synthesis to promote rapid proliferation [3].

However, when nucleotide metabolism is disrupted due to genetic defects, environmental stress, or disease states, it can lead to a series of pathological consequences [4]. Defects in adenosine deaminase (ADA) can lead to severe combined immunodeficiency. Deficiency of hypoxanthine–guanine phosphoribosyltransferase (HGPRT) causes Lesch–Nyhan Syndrome (LNS), characterized by neurological dysfunction and self‐injurious behavior [5]. Tumor cells commonly exhibit abnormal activation of the de novo nucleotide synthesis pathway to support their uncontrolled proliferation and genomic instability. For example, upregulation of pyrimidine synthesis rate limiting enzymes phosphoribosyl pyrophosphate synthase (PRPS) is common in various cancers [6]. In addition, abnormal accumulation of nucleotide metabolites can lead to various metabolic and inflammatory diseases. Intracellular purine metabolites such as ATP and ADP can act as danger signals (DAMPs) to activate inflammatory bodies and participate in atherosclerosis, autoimmune diseases, and other processes [7]. Infection and antiviral immunity: virus replication relies on the nucleotide metabolism mechanism of host cells, and the core of immune cells’ perception of viral nucleic acids (such as the cGAS–STING pathway recognizing cytoplasmic DNA) is also nucleotide derived signaling molecules [8, 9].

Therefore, a deeper understanding of nucleotide metabolism regulation mechanisms and disease associations not only helps to reveal the pathogenesis of various diseases, but also opens up new prospects for the development of novel diagnostic and therapeutic strategies targeting metabolic enzymes, intervening in metabolic flux, or utilizing metabolites as biomarkers [10, 11].

In this review, we provide a comprehensive analysis of the role of nucleotide metabolism in various diseases, including its regulatory mechanisms and therapeutic potential. We delve into the metabolic pathways of nucleotide synthesis and the pivotal roles of nucleotide‐metabolizing enzymes in DNA repair, oxidative stress, and immune modulation. Additionally, we explore innovative drug delivery approaches that target these metabolic pathways. Gaining insights into these intricate interactions is essential for developing more potent and precise treatment strategies, ultimately aiming to enhance treatment efficacy and the quality of life for patients.

## 2 Nucleotide Metabolic Pathway

The nucleotide metabolism pathway is a complex biochemical process in which nucleotides are synthesized and broken down in organisms and is crucial for cellular function. Nucleotides are not only the basic building blocks of DNA and RNA, but also participate in energy transfer, signal transduction, and coenzyme composition. Its metabolism mainly includes three pathways: de novo synthesis, salvage synthesis, and catabolism, which are finely regulated. The following will explain the system.

### 2.1 Nucleotides and Their Derivatives

Cyclic mononucleotides, which contain a single phosphate group, are defined by an intramolecular phosphodiester bond that connects the phosphorus atom to the sugar moiety's hydroxyl groups [12, 13]. Notably, second messengers include cyclic adenosine 5′‐monophosphate (cAMP) and cyclic guanosine 5′‐monophosphate (cGMP) within signal transduction pathways, acting as intracellular signaling molecules. Following stimulation with intercellular signaling molecules (first messengers)—including hormones, neurotransmitters, and mediators of inflammation—are formed within cells second messengers. Cyclic nucleotides beyond cyclic adenosine monophosphate [1] and cGMP are referred to as noncanonical cyclic nucleoside monophosphates, whose role as second messengers remains inconclusive.

Naturally occurring compounds that contain two nucleosides linked by a phosphate bridge of variable length (two to seven phosphates) are designated dinucleoside 5′,5′‐polyphosphates, also referred to as dinucleotides. Present in humans as well as other mammals, these molecules serve as strong endogenous agonists of the purinergic system [14, 15], and consequently affect cardiovascular physiology—manifested as vasodilatory responses, proliferative actions on vascular smooth muscle cells, and antiaggregatory effects on platelets. In contrast, nicotinamide adenine dinucleotide (NADH/NAD+), nicotinamide adenine dinucleotide phosphate (NADPH/NADP+), and flavin adenine dinucleotide (FADH2/FAD) participate in biochemical redox reactions as enzyme cofactors. Nonetheless, these substances are not dinucleotides in the strict sense, because their constituent groups—nicotinamide and flavin—do not belong to the nucleobase family. Rather, they are more accurately characterized as nicotinamide mononucleotide (NMN) and flavin mononucleotide derivatives, respectively. Finally, cyclic dinucleotides (cDNs) constitute a further class of signaling molecules with established importance in both prokaryotic and eukaryotic cells [16, 17, 18, 19].

In the biosynthesis of oligosaccharides and polysaccharides, glycosyltransferases rely on sugar nucleotides as glycosyl donors, given that these nucleotide‐activated monosaccharides provide the sugar moieties for glycosylation reactions. The same class of compounds also contributes to the glycosylation of proteins, lipids, and other biomolecules [20, 21]. Additionally, sugar nucleotides are utilized as building blocks for generating deoxysugars, aminodeoxysugars, chain‐branched sugars, and uronic acids. For plants and most animals, the biosynthetic route to vitamin C originates from sugar nucleotides. A notable exception is found in humans, who—through evolutionary loss—no longer possess the enzyme gulonolactone oxidase, which is responsible for converting L‐gulono‐γ‐lactone into ascorbic acid, the terminal step of vitamin C production [22]. As a consequence, dietary sources are indispensable for meeting human vitamin C requirements.

Nucleolipids, by definition, are amphipathic molecules carrying lipophilic groups that are covalently conjugated to a nucleoside or nucleotide core [23]. Among this family, cytidine diphosphate diacylglycerol is the most prominent example, functioning as a key intermediate in the synthesis of glycerophospholipids [24]. In recent years, these compounds have drawn growing interest as supramolecular carriers for nucleic acid and drug delivery.

### 2.2 Nucleotide Synthesis and Catabolism

Metabolic changes are a key characteristic of cellular life activities, and a deep understanding of nucleotide metabolism is crucial for advancing research on disease treatment [25].

**FIGURE 1:**
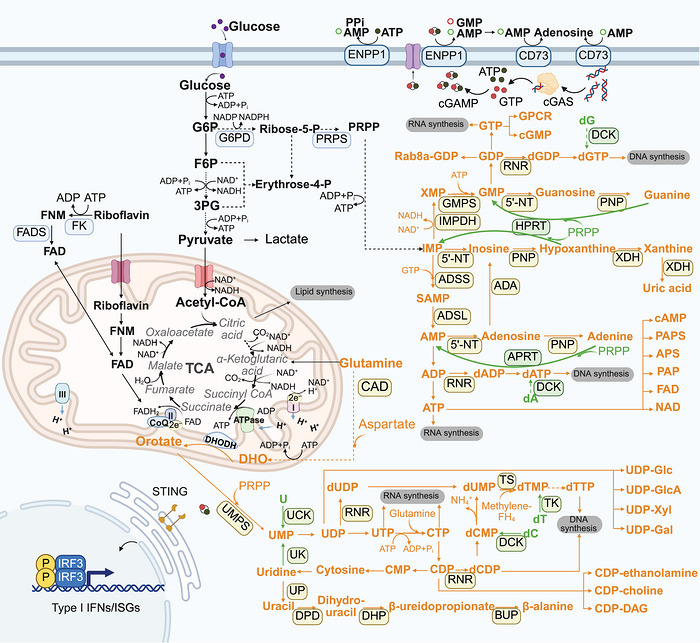
Metabolic pathway from glucose to nucleotides in cells. This schematic illustration depicts the metabolic pathways from glucose to nucleotides within cellular metabolism. The black pathways represent glycolysis in the cytoplasm and the tricarboxylic acid (TCA) cycle within the mitochondria. The orange pathways indicate the de novo synthesis of purines and pyrimidines, while the green pathways illustrate the salvage synthesis of purines and pyrimidines. This figure provides a visual overview of how glucose metabolism is intricately linked to the production of nucleotides, highlighting the key metabolic routes involved in cellular energy generation and nucleotide synthesis. Created with BioRender.com.

Purine and pyrimidine nucleotide synthesis occurs through two distinct pathways: the de novo pathway, which involves a series of energy‐consuming, multistep enzymatic reactions to incorporate small precursors into nucleotides, and the salvage pathway, where nucleosides or bases are converted to their corresponding nucleoside monophosphates (NMPs) through single phosphorylation or phosphoribosyl transferase reactions [26]. Significant differences exist between de novo and salvage synthesis of pyrimidines and purines [27, 28]. Essentially, the synthesis of various nucleotides revolves around the production of purine and pyrimidine bases [29] (Figure 1).

#### 2.2.1 Synthesis and Catabolism of Purine Nucleotides

There are two principal routes for the synthesis of purine nucleotides within the body: the de novo pathway and the salvage pathway [30]. The liver serves as the primary organ for the de novo synthesis of purine nucleotides, with the intestinal mucosa and thymus being secondary sites [6]. In contrast, the salvage synthesis predominantly occurs in the brain and bone marrow [31]. It has been established that the capacity for de novo purine nucleotide synthesis is not ubiquitous among all cells. Under normal conditions, the de novo pathway is regarded as the primary mechanism for the synthesis of purine nucleotides.

*De novo pathway*. The de novo pathway for the synthesis of purine nucleotides is a fundamental biological process that, with the exception of certain bacteria, is carried out by almost all living organisms [32]. Radioisotope tracing experiments have demonstrated that the precursors for the synthesis of purine bases are simple substances, with the elements for the synthesis of the purine ring coming from amino acids, CO_2_, and formyl groups (derived from tetrahydrofolate [THF]), among others [33]. The de novo synthesis of purine nucleotides occurs in the cytoplasm and begins with the stepwise synthesis of the purine ring starting from ribose‐5′‐phosphate. The process can be divided into two stages (Figure 1): first, the synthesis of inosine monophosphate (IMP), followed by the conversion of IMP into adenosine monophosphate [34] and guanosine monophosphate (GMP) [35].

*Synthesis of IMP* (Figure 1). The synthesis of IMP begins with ribose‐5′‐phosphate, which is produced in the pentose phosphate pathway (PPP) [36]. This compound undergoes activation by the action of phosphoribosyl pyrophosphate synthetase (PRPP synthetase), transferring one molecule of pyrophosphate from adenosine triphosphate (ATP) to the C1′ of ribose‐5′‐phosphate, resulting in the formation of 5′‐phosphoribosyl‐1′‐pyrophosphate (PRPP). Glutamine provides an amide group to replace the pyrophosphate on PRPP, forming 5′‐phosphoribosylamine (PRA), a reaction catalyzed by PRA transferase. This reaction is a key step in the de novo synthesis of purine nucleotides.

*The conversion of IMP to AMP and GMP* (Figure 1). IMP is an important precursor in the de novo synthesis of purine nucleotides and can be converted into AMP and GMP [37]. Unlike pyrimidine nucleotide synthesis, where the pyrimidine base is synthesized separately before being attached to ribose, purine nucleotides are synthesized directly on the ribose phosphate. This is a distinctive feature of purine nucleotide de novo synthesis.

*Regulation of de novo synthesis*. The de novo synthesis of purine nucleotides is primarily regulated by feedback inhibition from the end products AMP and GMP [38]. The key regulatory enzymes, PRPP synthetase and PRPP amidotransferase, are inhibited by the products IMP, AMP, and GMP [39]. Conversely, an increase in PRPP stimulates amidotransferase activity, accelerating the formation of PRA [40]. PRPP amidotransferase exists in an active monomeric form and an inactive dimeric form. IMP, AMP, and GMP can convert the active form into the inactive form, while PRPP has the opposite effect. In purine nucleotide synthesis regulation, PRPP synthetase plays a more significant role than amidotransferase, making its regulation crucial. Additionally, guanosine triphosphate (GTP) is required for the conversion of IMP to AMP, and ATP is required for the conversion of IMP to GMP [41]. GTP promotes AMP synthesis, while ATP promotes GMP synthesis. Excess AMP controls AMP synthesis without affecting GMP production, and excess GMP controls GMP synthesis without affecting AMP production. This crossregulation is important for maintaining the balance between ATP and GTP levels.

There are two salvage pathways for purine nucleotide synthesis [42]. The first involves the reuse of existing purine bases or nucleosides to synthesize purine nucleotides, which is simpler and less energy consuming. This process involves two enzymes: adenine phosphoribosyltransferase (APRT) and HGPRT [43]. Salvage synthesis also utilizes PRPP to provide ribose phosphate. APRT is inhibited by AMP, while HGPRT is inhibited by IMP and GMP. The second pathway involves the phosphorylation of adenine nucleosides to generate adenine nucleotides, catalyzed by adenine kinase. Other purine nucleosides do not have specific kinases in the body. Salvage pathways mainly involve the reactions catalyzed by phosphoribosyltransferases. Salvage synthesis is significant for saving energy and amino acids compared with de novo synthesis and is crucial in tissues like the brain and bone marrow, which lack the complete de novo synthesis enzyme system. For instance, patients with complete HGPRT deficiency suffer from LNS, a genetic metabolic disorder [44].

The production of deoxynucleotides occurs at the diphosphonucleotide level. In the body, deoxynucleotides are not synthesized by first forming deoxyribose and then attaching bases and phosphate [33]. Instead, they are generated through the direct reduction of the corresponding nucleotides, where hydrogen replaces the hydroxyl group on the C2 position of the ribose molecule. Except for deoxythymidine monophosphate (dTMP), which is derived from dUMP, other deoxynucleotides are formed at the diphosphonucleotide level (where N represents bases like A, G, U, C) and are catalyzed by ribonucleotide reductase [45].

Purine nucleotide antimetabolites are analogs of purines, amino acids, or folates [46]. They primarily interfere with or block purine nucleotide synthesis through competitive inhibition or “mimicry” mechanisms, thereby disrupting nucleic acid and protein biosynthesis. 6‐Mercaptopurine (6‐MP) has a structure similar to hypoxanthine and directly affects HGPRT, preventing the transfer of PRPP to guanine and hypoxanthine, thus inhibiting the salvage pathway of purine synthesis. In the body, 6‐MP can also convert into 6‐MP nucleotides with PRPP. Because 6‐MP nucleotides resemble IMP, they feedback inhibit PRPP amidotransferase, disrupting the formation of PRA and blocking de novo purine nucleotide synthesis. Due to these mechanisms, 6‐MP is a potent inhibitor of purine nucleotide synthesis and thus can suppress tumor growth [47]. Methotrexate (MTX) and aminopterin are folate analogs that competitively inhibit dihydrofolate reductase (DHFR). This inhibition prevents the reduction of folate to dihydrofolate and THF, thereby depriving purine molecules of essential one‐carbon units (C8 and C2) needed for purine nucleotide synthesis. MTX is used clinically for the treatment of leukemia and other conditions [48]. Azaserine, structurally similar to glutamine, interferes with glutamine's role in purine nucleotide synthesis, thereby inhibiting purine nucleotide production [49]. It is important to note that these drugs lack specificity for tumor cells and can also adversely affect rapidly proliferating normal tissues, leading to significant side effects.

The final product of purine nucleotide catabolism is uric acid [50]. The breakdown of nucleotides within cells is similar to the digestion process of nucleotides from dietary sources. Initially, nucleotides are hydrolyzed into nucleosides by nucleotidases. Nucleosides are then phosphorylated by nucleoside phosphorylases, yielding free bases and ribose‐1‐phosphate. Purine bases can either participate in nucleotide salvage pathways or be further hydrolyzed [51]. In humans, purine bases are ultimately degraded to uric acid, which is excreted in the urine [52]. The simplified pathway is as follows: AMP is converted to hypoxanthine, which is then oxidized to xanthine by xanthine oxidase (XO), eventually forming uric acid. GMP is converted to guanine, which is subsequently converted to xanthine, and finally to uric acid. Purine deoxynucleotides undergo similar metabolic pathways. The breakdown of purine nucleotides primarily occurs in the liver, small intestine, and kidneys, where XO is highly active. Uric acid, being relatively insoluble in water, can accumulate in the blood under conditions such as high purine intake, extensive nucleic acid breakdown (as seen in leukemia or malignancies), or impaired renal excretion. This can lead to hyperuricemia. Allopurinol is commonly used to treat gout. It is structurally similar to hypoxanthine, except that in allopurinol, the positions of N7 and C8 are swapped, allowing it to inhibit XO and thereby reduce uric acid production. Xanthine and hypoxanthine are more water soluble than uric acid and do not form crystals. Additionally, allopurinol reacts with PRPP to form allopurinol ribonucleotide, which depletes PRPP and, due to its structural similarity to IMP, inhibits the enzymes involved in de novo purine nucleotide synthesis. These effects collectively reduce purine nucleotide synthesis.

#### 2.2.2 Synthesis and Catabolism of Pyrimidine Nucleotides

The biosynthesis of pyrimidine nucleotides within the body proceeds via two distinct pathways: de novo synthesis and salvage synthesis (Figure 1). The de novo synthesis of pyrimidine nucleotides is a more straightforward process compared with that of purine nucleotides. Utilizing radioisotope tracing techniques, it has been established that the components necessary for the assembly of the pyrimidine moiety are derived from sources such as glutamine, CO_2_, and aspartate. The hepatic synthesis of pyrimidine nucleotides occurs in both the cytosolic and mitochondrial compartments of the cell. In contrast to the de novo synthesis of purine nucleotides, the construction of pyrimidine nucleotides is initiated with the synthesis of orotic acid, a molecule that contains the pyrimidine ring structure, which is subsequently conjugated with phosphoribosyl to form the nucleotide.

*Synthesis of uridine nucleotides* [53]. The synthesis of the pyrimidine ring begins with the formation of carbamoyl phosphate. As discussed in the amino acid metabolism chapter, carbamoyl phosphate is also a precursor for urea synthesis. However, the carbamoyl phosphate required for urea synthesis is generated in the liver mitochondria by carbamoyl phosphate synthetase (CPS) I, whereas the carbamoyl phosphate used in pyrimidine synthesis is synthesized in the cytoplasm from glutamine as the nitrogen source, catalyzed by CPS II. These two synthetases have different properties. The carbamoyl phosphate generated above then reacts with aspartate to form carbamoyl aspartate, catalyzed by aspartate transcarbamoylase in the cytoplasm. The latter is dehydrated to form dihydroorotate, which has a pyrimidine ring structure, by dihydroorotate dehydrogenase (DHODH), and further oxidized to form orotate [54]. Although orotate is not a pyrimidine‐base constituting nucleic acids, it can combine with PRPP to form orotate nucleotide under the catalysis of orotate phosphoribosyltransferase. Orotidine 5′‐monophosphate is then decarboxylated to form uridine monophosphate [55] by orotate nucleotide decarboxylase. Cytidine nucleotides and thymidine nucleotides can be derived from UMP [56]. In eukaryotic cells, the first three enzymes of pyrimidine nucleotide synthesis, namely, CPS II, aspartate transcarbamoylase, and dihydroorotase, are present on the same polypeptide chain of approximately 200 kDa, forming a multifunctional enzyme. The latter two enzymes are also multifunctional enzymes located on the same polypeptide chain. This arrangement facilitates a more uniform rate of pyrimidine nucleotide synthesis.

*Synthesis of cytidine triphosphate*. UMP is converted to uridine triphosphate (UTP) through the consecutive actions of uridylate kinase and diphosphate nucleoside kinase (Figure 1). Then, cytidine triphosphate (CTP) synthase catalyzes the conversion of UTP to CTP by consuming one ATP molecule and transferring an amino group from glutamine.

*Regulation of de novo synthesis* [57]. In bacteria, aspartate transcarbamoylase is the main regulatory enzyme for de novo pyrimidine nucleotide synthesis. In mammalian cells, however, the regulation is primarily mediated by CPS II, which is inhibited by UMP. Both enzymes are regulated by feedback mechanisms [58]. Additionally, the multifunctional enzymes involved in the initial and final steps of UMP synthesis in mammalian cells can be regulated by inhibition or derepression. Isotope labeling experiments show that the synthesis of pyrimidines and purines is coordinated, with their synthesis rates usually proceeding in parallel. Since PRPP synthetase is required for both pyrimidine and purine nucleotide synthesis, it can be simultaneously inhibited by feedback from both pyrimidine and purine nucleotides.

The salvage pathway for pyrimidine nucleotides is similar to that of purine nucleotides. Pyrimidine phosphoribosyl transferase has been purified from human red blood cells; it utilizes uracil, thymine, and lactate as substrates (in fact, this enzyme is the same as the lactate phosphoribosyl transferase mentioned earlier) but does not act on cytosine. Uridine kinase is also a salvage enzyme, catalyzing the conversion of uridine to uridine monophosphate. Deoxythymidine can be converted to dTMP by thymidine kinase (TK). This enzyme has low activity in normal liver but increased activity in regenerating liver and malignant tumors, and its activity is correlated with the degree of malignancy [59].

Pyrimidine nucleotide antimetabolites are also analogs of pyrimidines, amino acids, or folic acid. Similar to purine nucleotides, pyrimidine nucleotide antimetabolites, such as 5‐fluorouracil 5 [35], affect metabolism and have antitumor effects. 5‐FU itself is biologically inactive and must be converted into 5‐fluoro‐deoxyuridine monophosphate (FdUMP) and 5‐fluoro‐UTP (FUTP) in the body to be effective. FdUMP, with a structure similar to dUMP, inhibits thymidylate synthase (TS), blocking dTMP synthesis. FUTP can be incorporated into RNA as FUMP, disrupting RNA structure and function [60]. Analogous compounds like nitrogen mustard, which mimics glutamine, can inhibit CTP production; MTX interferes with folate metabolism, preventing the methylation of dUMP to dTMP, thus affecting DNA synthesis. Additionally, some nucleoside analogs that alter ribose structure, such as cytarabine and cladribine, are important anticancer drugs [61]. Cytarabine inhibits the conversion of CDP to dCDP, also affecting DNA synthesis.

Pyrimidine nucleotide degradation ultimately produces NH3, CO_2_, β‐alanine, and β‐aminoisobutyric acid. Pyrimidine nucleotides are first dephosphorylated and de‐ribosylated by nucleotide and nucleoside phosphatases to yield pyrimidine bases, which are then further degraded. Cytosine deaminates to uracil. Uracil is reduced to dihydrouracil and then hydrolyzed to produce NH3, CO2, and β‐alanine [62]. Thymine degrades to β‐aminoisobutyric acid, which is excreted in urine or further metabolized. The amount of β‐aminoisobutyric acid in the urine increases in individuals consuming DNA‐rich foods or those undergoing radiation or chemotherapy. Pyrimidine base degradation mainly occurs in the liver. Unlike purine base degradation that produces uric acid, pyrimidine base degradation products are all water soluble [63].

#### 2.2.3 Key Rate Limiting Enzymes in Nucleotide Metabolism

The key rate limiting enzymes in the nucleotide metabolism network, as the “valves” of metabolic pathways, control the synthesis and decomposition rates of nucleotides, thereby profoundly affecting the fate of cells. These enzymes not only include PRPP synthase and glutamine PRPP amide transferase in purine synthesis, as well as CPS II in the initial stage of pyrimidine synthesis, but also extend to key nodes connecting different organelles, such as DHODH located in mitochondria. In depth research on the regulatory mechanisms of these enzymes, especially their abnormalities in disease states, provides a core perspective for understanding disease occurrence and developing new therapies.

DHODH plays a unique and central role in the de novo synthesis pathway of pyrimidine nucleotides. It is located on the inner membrane of mitochondria, catalyzing the oxidation of dihydroorotic acid to orotic acid and transferring electrons to coenzyme Q, thereby directly coupling pyrimidine synthesis with the mitochondrial electron transport chain and cellular energy metabolism. This unique cellular localization enables its function to go beyond simple substrate conversion and become a key hub for integrating biosynthesis and energy states. In rapidly proliferating cells such as tumors, the expression of DHODH is often significantly upregulated by oncogenes (such as c‐Myc) to meet their massive demand for pyrimidine nucleotides due to uncontrolled proliferation [64]. Overexpressed DHODH not only directly supports DNA replication and transcription by maintaining a large pyrimidine pool, but also shapes a favorable microenvironment for tumor survival and progression by affecting signaling pathways such as NF‐κB [65], and inhibits cell differentiation programs, maintaining tumor cells in an immature proliferative state.

The inhibition of DHODH has been proven to be an effective anticancer strategy, and its mechanism of action deeply reveals the close relationship between nucleotide metabolism and cellular stress [66]. When DHODH is inhibited by small molecule inhibitors, it triggers a series of chain reactions [67]. First, the interruption of pyrimidine synthesis directly blocks DNA synthesis and inhibits cell proliferation. More importantly, inhibition can lead to the accumulation of oxidative coenzyme Q in mitochondria, interfere with the function of the electron transport chain, and cause a decrease in mitochondrial membrane potential and a large outbreak of ROS. This intense oxidative stress can cause mitochondrial damage, leading to leakage of mitochondrial DNA and other contents into the cytoplasm. The leaked mtDNA serves as a danger signal (DAMP) that can activate innate immune sensing pathways such as cGAS–STING [68]. At the same time, high levels of ROS can also activate NLRP3 inflammasome, jointly driving a strong inflammatory response and secreting cytokines such as Type I interferon (IFN‐I) and IL‐1 β. Therefore, the effects of DHODH inhibition are dual: direct antiproliferative effects and indirect cell killing and immune regulatory effects triggered by the metabolic–oxidative stress–inflammatory axis.

In addition to DHODH, other rate limiting enzymes in the nucleotide metabolism network are also important disease targets. For example, in purine synthesis, IMP dehydrogenase (IMPDH) is crucial for guanine nucleotide synthesis, and its activity is particularly important in lymphocyte proliferation [69]. The inhibitor mycophenolate mofetil has been widely used in immunosuppressive therapy [70]. RNR, as the sole provider of DNA synthesis precursor dNTP, is a classic chemotherapy target, and inhibitors such as hydroxyurea exert anticancer effects by interfering with its function [71]. These examples collectively demonstrate that targeting key enzymes involved in nucleotide metabolism can precisely intervene in the fundamental needs of rapidly proliferating cells.

In summary, nucleotide metabolism rate limiting enzymes represented by DHODH are the core regulatory components of cellular function. Future research will focus more on optimizing such strategies, such as developing more selective inhibitors, exploring combination therapies with immunotherapy or antioxidant pathway inhibitors to overcome potential drug resistance and maximize treatment windows, ultimately achieving safer and more effective targeted therapies for tumor metabolism.

### 2.3 Abnormal Nucleotide Metabolism in Tumor Cells

To proliferate, tumor cells often exhibit higher anabolic activity in nucleotide synthesis compared with normal tissues, resulting in elevated activity of nucleotide synthesizing enzymes, particularly deoxyribonucleotidases. In normal cells, the expression of deoxyribonucleotidases fluctuates in accordance with the cell cycle. However, cells transformed by oncogenic viruses lack this variability in expression, losing normal regulatory function and maintaining high expression levels, which subsequently leads to increased DNA synthesis [72].

Nucleotide synthesis occurs through two fundamental pathways: de novo synthesis and the salvage pathway. Normal cells primarily rely on the salvage pathway for nucleotides, which is more economical and energy efficient, alongside purines and pyrimidines. Due to their rapid growth, tumor cells cannot fulfill their fast‐growing needs solely through the salvage pathway and thus utilize both pathways. In some tumors, de novo synthesis even becomes the primary means of nucleotide synthesis [73]. Since de novo synthesis involves intricate enzymatic reactions starting from simple precursor molecules to gradually synthesize nucleotides, these precursor molecules are vital for nucleotide synthesis. These precursor molecules can originate from various sources, particularly intermediates of glucose metabolism, such as nitrogen possibly derived from glutamine metabolites. This elucidates the enhanced ability of tumor cells to uptake glucose and glutamine, which correlates with their augmented nucleotide synthesis. Ribose‐5‐phosphate (R5P), a required raw material in the process of nucleotide synthesis, is produced from glucose‐6‐phosphate (G6P) through the PPP of glucose metabolism [74]. Typically, PPP is regulated by both oncogenes and tumor suppressor genes. Tumor suppressor genes like p53 can bind to and inhibit the activity of glucose‐6‐phosphate dehydrogenase (G6PD), the rate‐limiting enzyme of the first step in PPP. Consequently, in normal cells, glucose is mainly utilized for glycolysis and the tricarboxylic acid cycle to produce energy [75]. However, in tumor cells, mutations in p53 abolish its inhibitory effect on G6PD, enhancing G6PD activity in the cytoplasm. As a result, a significant portion of glycolytic products are directed toward PPP rather than entering mitochondria for the tricarboxylic acid cycle to produce ATP. Thus, PPP plays a pivotal role in the anabolic processes of tumor cells, providing not only the necessary substance R5P for nucleotide synthesis but also NADPH, a cofactor required for lipid and nucleotide synthesis [76]. Studies have shown that PPP is also associated with lipid metabolism, as sterol regulatory element‐binding protein (SREBP) can upregulate G6PD expression, and SREBP expression is increased in most tumors. Given that G6PD activity is enhanced in many tumor cells, G6PD may represent a potential therapeutic target for cancer treatment. For instance, 6‐aminonicotinamide, an inhibitor of G6PD, has demonstrated anticancer effects in studies.

The activity of certain de novo nucleotide synthesizing enzymes is higher in tumor cells, such as DHFR, which catalyzes the production of THF from dihydrofolate, providing one‐carbon units for de novo thymidylate synthesis. DHFR is expressed at higher levels in colorectal cancer tissues compared with adjacent normal tissues. Another example is TS, which catalyzes the conversion of deoxyuridine to dTMP, a precursor for DNA synthesis, and its expression is increased in numerous tumors.

The intracellular pool of dNTPs produces a wide array of effects on DNA replication, DNA repair, mutagenesis, and cancer development. The dNTP pool is achieved through allosteric regulation of RNR and SAM and HD domain containing deoxynucleoside triphosphate triphosphohydrolase 1 (SAMHD1), which exhibit cell cycle‐dependent activity [77]. DNA damage triggers a roughly fourfold increase in S‐phase dNTP levels [78], while the recruitment of RNR and SAMHD1 to the damaged sites ensures stringent control over the dNTP supply needed for repair [79]. As part of the salvage pathway, the degradation of dNTPs is carried out by deaminases and phosphorylases, in conjunction with the mammalian triphosphohydrolase SAMHD1 [80, 81].

SAMHD1 is downregulated in various cancers and exhibits different activities toward drug substrates. Early in human cancer, there is evidence that DNA replication stress can lead to genomic instability [82]. The negative regulatory role of SAMHD1 in intercellular dNTP metabolism and DNA replication stress directly contributes to its association with multiple cancers. A growing body of evidence indicates that SAMHD1 is downregulated in several human cancers, such as chronic lymphocytic leukemia (CLL) [79, 83], lung cancer [84], cutaneous T‐cell lymphoma (CTCL) [85], acute myeloid leukemia [86], and colon cancer [87]. This observation is further supported by over 300 patient‐derived SAMHD1 mutations that have been deposited in online databases, including COSMIC, cBioPortal, and NCI GDC (COSMIC: COSG646673) [88, 89, 90, 91]. The SAMHD1 gene harbors cancer‐related mutations across its entire sequence, and these variants have been shown to influence SAMHD1 activity and expression levels in vivo [79, 83]. Among 160 CLL cases examined, SAMHD1 mutations were present in four patients, representing 2.5% of the cohort [92]. The incidence of acquired SAMHD1 mutations in patients with recurrent or chemotherapy‐refractory CLL is reported to be 11%, compared with 3% in the pretreatment group [79]. These findings suggest that SAMHD1 mutations may be important drivers of CLL progression and could potentially be used as biomarkers for CLL prognosis. Multiple tumor types—including CLL [93], myeloma [94], breast cancer [95], lung cancer [96], colorectal cancer [97, 98], pancreatic cancer [99], and glioblastoma [100]—harbor somatic mutations in the SAMHD1 gene. In a separate context, CTCL, one of the non‐Hodgkin's lymphoma subtypes, is distinguished by the accumulation of neoplastic CD4+ T lymphocytes in the skin [101]. Compared with healthy donors, SAMHD1 mRNA and protein levels are decreased in peripheral blood mononuclear cells from CTCL patients [85]. In CTCL, the functional significance of SAMHD1 downregulation remains unresolved with respect to whether it drives lymphomagenesis, whether its re‑expression reverses the disease phenotype, and whether its expression levels correlate with clinical outcome. Turning to lung cancer, reduced SAMHD1 transcript and protein have been observed [84], although their relationship with disease progression or prognosis is yet to be confirmed. Ectopic SAMHD1 expression in lung cancer‑derived cell lines elicits antiproliferative effects in vitro, together with elevated intracellular dNTP concentrations. This growth‑inhibitory property is recapitulated in HeLa cells upon wild‑type SAMHD1 overexpression, whereas a mutant deficient in dNTPase activity fails to suppress proliferation [79]. Although its expression is reduced in cancerous tissues, residual SAMHD1 activity persists at low concentrations and is sufficient to diminish the effectiveness of anticancer nucleoside mimetics—cytosine arabinoside [102], for example—by catalyzing the hydrolysis of dNTPs that bear canonical or noncanonical nucleobases and sugar moieties, including the triphosphate form of cytosine arabinoside [103, 104, 105]. SAMHD1‐mediated nucleoside triphosphate analog hydrolysis diminishes intracellular levels of therapeutic agents, ultimately weakening their anticancer efficacy. Therefore, SAMHD1 is considered as a clinical biomarker of cytosine arabinoside C sensitivity in cancer and has been suggested coadministration of Vpx and cytosine arabinoside C during treatment to enhance efficacy [106, 107, 108]. Representing one of the six established classes of anti‐HIV drugs, NRTIs were among the first antiretroviral agents approved for mitigating the clinical course of acquired immune deficiency syndrome [109]. Prior to their incorporation into proviral DNA during reverse transcription, NRTIs undergo phosphorylation by cellular kinases to yield 5′‐triphosphate derivatives that then outcompete dNTPs for DNA chain integration [110]. Integration of NRTIs into the nascent viral DNA strand during reverse transcription results in chain termination. NRTIs are particularly effective against HIV‐1 in myeloid cells because SAMHD1 expression reduces dNTPs level that can compete with chain terminators during proviral synthesis, thereby supporting the activation of certain NRTIs [111] and allowing SAMHD1‐mediated cleavage of NRTI triphosphates at rates significantly lower than cellular dNTPs, which enables these drugs to effectively compete for the HIV‐1 RT active site [112]. Now, with a better understanding of the role of SAMHD1 in the efficacy of anti‐HIV‐1 drugs, several nucleotide analogs that were initially not classified as antiretrovirals have been found to be effective anti‐HIV‐1 drugs in cells with low dNTP pools. Notably, two herpesvirus‐specific drugs and an anticancer drug have been shown to have enhanced anti‐HIV‐1 activity in cells with low dNTP pools. These studies suggest the possibility of reusing previously overlooked anti‐HIV‐1 drugs.

The modulation of cellular dNTP pools represents a key therapeutic strategy against cancer, particularly in the context of DNA damage response hyperactivation, where elevated nucleotide levels may drive tumorigenesis. De novo nucleotide biosynthesis is governed by RNR, which catalyzes the pivotal conversion of ribonucleotides to deoxyribonucleotides as the rate‐determining step [113]. This enzyme adopts a heterotetrameric structure that is maintained at uniform levels across all stages of the cell cycle, with biosynthetic activity persisting even in nondividing cells [114]. Transcription of the gene specifying the catalytically active R1 subunit is subject to regulation by both replicative stress and cell‐cycle‐dependent cues. RNR transcription in the S phase is 10–20 times higher than in the G1 phase [115]. With the help of the p53‐induced subunit R2, RNR serves a dual function in cellular metabolism: it not only drives de novo dNTP production but also translocates to DNA break sites to fuel the nucleotide demands of repair machineries. Neoplastic cells exhibit a strong dependency on RNR for de novo dNTP synthesis, and its overexpression—a diagnostic feature of diverse malignancies—promotes both nucleotide surplus and unrestrained mitotic activity. The antineoplastic agent hydroxyurea, which combines radical‐neutralizing and iron‐sequestering properties, abrogates RNR function by targeting the catalytic domain of the R2 homodimer [116]. Currently clinically used RNR inhibitors, for example, hydroxyurea and gemcitabine, are constrained by several drawbacks, namely, pronounced cytotoxicity, brief circulating half‐lives, and a propensity for resistance emergence under single‐agent administration. The evolving landscape of nucleotide synthesis‐targeted therapy features small‐molecule regulators of subunit activity alongside oligonucleotide‐based and gene‐therapeutic strategies that act on RNR [117].

The regulatory mechanisms of SAMHD1 and RNR expression and activity are similar, both depending on the cell cycle and being tightly controlled within the cell. Both enzymes are oligomeric assemblies that feature two allosteric binding sites, which together survey the nucleotide environment and adjust catalytic rates accordingly [77]. The allosteric control of SAMHD1 dNTPase unfolds in a defined sequence: initial engagement of the primary site by dGTP or GTP is followed by dNTP occupation of the secondary site, a step that elicits a conformational alteration of the active site cleft [118]. SAMHD1 further serves as a sensor for the dNTP pool, orchestrating a negative feedback mechanism that operates in opposition to the anabolic direction of RNR [119]. In this regulatory network, dATP stands out as the primary effector of dNTP homeostasis, displaying preferential binding to SAMHD1's second allosteric site [120]. The ratio of dATP to ATP also dictates RNR activity in a reciprocal manner. Elevated dATP levels inhibit RNR catalytic activity, while elevated ATP concentrations stimulate its anabolic activity [117].

**FIGURE 2:**
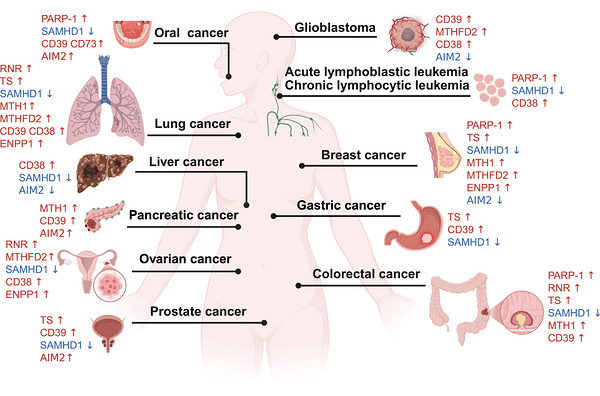
Altered expression of nucleotide metabolizing enzymes in various cancers. This schematic shows aberrant nucleotidase expression in different types of tumors, with arrow directions indicating upregulation or downregulation. The enzymes play critical roles in nucleotide synthesis and repair, which are essential for cancer cell proliferation and survival. Created with BioRender.com.

CD38 is a cell‐surface enzyme with dual catalytic functions, exhibiting both NADase and cyclase activities. CD38 expression is observed not only on immunocytes such as lymphocytes and plasma cells in the human body but is also abnormally expressed in various tumor cells and is closely related to the tumorigenesis and disease progression of tumors (Figure 2). CD38 has garnered considerable research interest over the past several decades. CD38 was first characterized as the T‐cell surface antigen T10, which was later recognized as both a T‐cell marker [121] and a thymocyte surface protein [122]. Subsequently, CD38 expression was detected on dendritic cells [123], neutrophils [124], monocytes and macrophages under inflammatory conditions [125], and lymphocytes [126]. CD38 is expressed in multiple tumors and plays a tumor‐promoting role, including in solid tumors such as cervical cancer [127], neuroblastoma [128], esophageal cancer [40], liver cancer [129], melanoma [130], and lung cancer [131], as well as in hematological tumors such as multiple myeloma and CLL [131, 132, 133].

Ectonucleotide pyrophosphatase phosphodiesterase‐1 (ENPP1) is a bifunctional ectoenzyme with both pyrophosphatase and phosphodiesterase catalytic functions. By participating in purinergic signaling, this enzyme critically influences a broad spectrum of physiological systems. These include immune responses, cardiovascular performance, neurological function, and hematological homeostasis [134, 135]. Multiple cancer types—among them lung cancer [136], ovarian cancer [137], and breast cancer [138]—exhibit elevated ENPP1 expression (Figure 2). In these malignancies, heightened ENPP1 expression predicts a poorer prognosis, raising interest in this molecule as a candidate for immune‐based cancer therapies. Studies have demonstrated that inactivation of ENPP1 can restrain tumor dissemination, augment the accumulation of immune cells within the tumor microenvironment (TME), and improve survival rates [55, 139]. When considered in light of its physiological functions and its link to aggressive neoplastic disease, ENPP1 stands out as an attractive target for the formulation of new antineoplastic treatment regimens.

The central role of nucleotide metabolism in driving cell proliferation, oncogenesis, and disease progression has made the suppression of nucleotide synthesis a mainstay therapeutic approach for cancers, infectious diseases, and immune‐related disorders [116]. The earliest antineoplastic pharmacotherapies were dominated by cytotoxic agents targeting nucleotide metabolism—specifically, nucleoside and nucleobase analogs, with thiopurines and fluoropyrimidines being representative members [140] (Table 1). These antimetabolites are structural mimetics of physiological nucleotides, allowing them to perturb nucleotide metabolism and curtail nucleic acid synthesis [141]. Historically, RNR inhibitors were among the first anticancer agents to enter clinical practice [116], and they retain their relevance in contemporary oncology. Gemcitabine, a representative nucleoside analog, serves as a standard treatment for pancreatic adenocarcinoma and is also indicated for breast, bladder, and non‐small cell lung cancer [142]. Nonetheless, gemcitabine resistance remains a frequent obstacle, often attributable to upregulation of nucleotide biosynthetic routes or perturbations in cellular nucleoside transport [143]. Azaserine and 6‐MP are employed in tumor treatment by interfering with de novo purine nucleotide synthesis [144]. Folate analogs such as MTX and aminopterin inhibit DHFR [145], thereby inhibiting purine nucleotide synthesis, and are utilized to treat malignancies such as leukemia. The active metabolite of 5‐fluorouracil, 5‐fluorodeoxyuridine, binds to TS to form a stable structure and inhibit its activity [146]. Last, the selective suppression of enzymes that drive de novo and salvage nucleotide biosynthesis—which are fueled by glucose and glutamine—has also found clinical application as a component of multimodal anticancer therapy. For most nucleoside analogs in clinical use, therapeutic action is achieved through DNA chain integration, the fidelity of which is governed by the availability of properly balanced dNTP pools during the replication process. Excessive intracellular dNTP levels can potentially outcompete these nucleoside analogs, leading to chemotherapy resistance [147]. Therefore, these drugs can be paired with other pharmacologic approaches, RNR inhibitors being one such example, given their capacity to lower dNTP concentrations.

**TABLE 1:** Clinical drugs targeting nucleotide metabolic enzymes.

Name	Structural formula	Target enzyme	Indication	Clinical trial phase	References
Olaparib	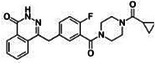	PARP1	Breast cancer Ovarian cancer Pancreatic cancer Prostate cancer	IV	[148]
Rucaparib	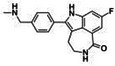		Prostate cancer Ovarian cancer	III	[149]
Talazoparib	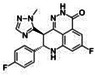		Breast cancer Prostate cancer	III	[150]
Niraparib	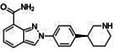		Epithelial ovarian cancer Fallopian tube cancer Primary peritoneal cancer	IV	[151]
Veliparib (ABT‐888)	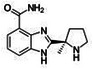		Triple‐negative breast cancer Non‐small cell lung cancer (NSCLC)	III	[152]
Fluzoparib	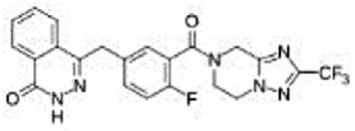		Breast cancer Ovarian cancer Pancreatic cancer Prostate cancer Lung cancer	III	[153]
Gemcitabine	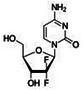	RNR	Testicular cancer Breast cancer Ovarian cancer NSCLC Pancreatic cancer	IV	[154]
Cytarabine	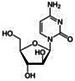	RNR	Acute myeloid leukemia (AML) Acute lymphoblastic leukemia (ALL) Chronic myeloid leukemia (CML)	IV	[155]
Capecitabine	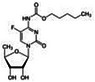	TS	Breast cancer Gastric cancer Colorectal cancer	IV	[156]
5‐Fluorouracil	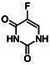		Colorectal cancer Stomach cancer Pancreatic cancer Breast cancer Cervical cancer	IV	[156]
Pemetrexed	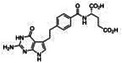		Pleural mesothelioma NSCLC	IV	[157]
Padnarsertib (KPT‐9274)	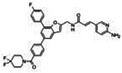	NAMPT	AML ALL Kidney cancer Breast cancer	I	[158]
Daporinad (APO866/FK866)	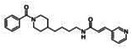		Melanoma Cutaneous T‐cell Lymphoma B‐cell chronic Lymphocytic leukemia	II	[159]
Oleclumab	IgG1λ	CD73	NSCLC Pancreatic cancer Colorectal cancer	III	[160]
Quemliclustat (AB680)	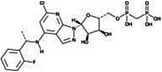	CD73	Colorectal cancer NSCLC Metastatic pancreatic Ductal adenocarcinoma (PDAC)	III	[161]
Mupadolimab	IgG1κ		Bladder cancer Breast neoplasms Castration resistant Prostate cancer Cervical cancer Colorectal cancer	III	[162]
IPH5301	IgG1		Breast cancer Pancreatic cancer Gastric cancer Lung cancer Ovary cancer	I	[163]
TTX‐030	IgG4	CD39	Pancreatic cancer Lymphoma	II	[164]
IPH5201	IgG1		NSCLC Melanoma Fibrosarcoma	II	[163]
SRF617	IgG4		Prostate cancer Advanced solid tumors	II	[165]
C‐176	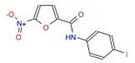	STING	Systemic lupus erythematosus (SLE) Aicardi–Goutières syndrome (AGS) stimulator of IFN genes‐associated vasculopathy with onset in infancy (SAVI)	Preclinical stage	[166]
H‐151	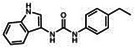	STING	SLE, AGS, SAVI	Preclinical stage	[166]
NO_2_‐FAs	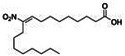		SLE, AGS, SAVI	/	[167]
BB‐Cl‐amidine	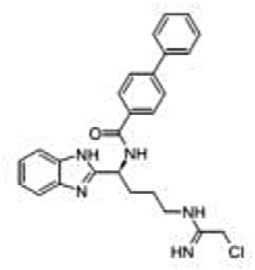		SLE, AGS, SAVI COPA syndrome Amyotrophic lateral sclerosis (ALS) Parkinson's disease	Experimental drug	[168]
Palbociclib	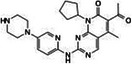		Advanced breast cancer	IV	[169]
SN‐011	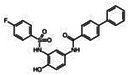		SLE, AGS	Preclinical stage	[170]
STING‐IN‐2 (C‐170)	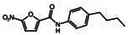		Inflammatory bowel disease	Preclinical stage	[171]
Suramin	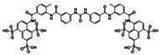	cGAS	Prostate cancer Autism spectrum disorder Multiple myeloma Plasma cell neoplasm	III	[172]
Oxychloroquine	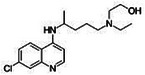		Acute rheumatoid arthritis Lupus erythematosus Porphyria cutanea tarda (PCT)	IV	[173]
Perillaldehyde	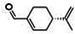	cGAS	AGS, SLE	Preclinical stage	[174]
PF‐06928215	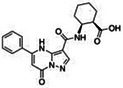		SLE	Preclinical stage	[173]

In summary, azaserine, 6‐MP, MTX, aminopterin, and 5‐FU are currently conventional antimetabolite chemotherapy drugs used in clinical practice, and their anticancer effects are predicated on the heightened activity of de novo nucleotide synthesizing enzymes in tumor cells.

## 3 Nucleotide Metabolism Reprogramming in Tumorigenesis and Progression

This section reviews the reprogramming of nucleotide metabolism during tumorigenesis and progression and its biological and therapeutic implications. We begin by outlining the core features of tumor metabolic reprogramming (Section 3.1), including the shift in energy metabolism, enhanced anabolic metabolism and the signaling functions of metabolites, and highlight the central role of nucleotide metabolism in supporting proliferation and maintaining redox balance. Subsequently (Section 3.2), we systematically survey key enzymes and pathways involved in DNA damage repair, replication and drug response, such as PARP1, RNR, TS, HGPRT, various nucleoside kinases and NDP and NTP kinases, and discuss their roles in drug resistance and as biomarkers. Next (Section 3.3), we examine regulators associated with oxidative stress and dNTP pool homeostasis, including MTH1, MTHFD2, SAMHD1, NAD+, and IMPDH, and their impact on genomic stability. Finally (Section 3.4), we summarize therapeutic strategies and emerging delivery systems targeting nucleotide metabolism, including adenosine‐ and ATP‐targeting approaches, STING agonists, nanoparticle, carrier‐free and vector‐based delivery systems, and Mn2+‐augmented radiotherapy, and discuss current challenges in clinical translation and future research directions. This section aims to construct a logically coherent knowledge chain from molecular mechanisms to therapeutic intervention, enabling readers to grasp the critical nodes of nucleotide metabolism in tumor biology and precision medicine.

### 3.1 Core Features of Tumor Cell Metabolic Reprogramming

Tumor cell metabolic reprogramming refers to the adaptive changes in cellular metabolic pathways driven by oncogenic signals to meet the demands of unlimited proliferation, increased biosynthesis, and maintenance of redox balance [175]. This concept was first proposed by Otto Warburg in the 1920s, with its core feature being that tumor cells preferentially obtain energy through glycolysis even in the presence of sufficient oxygen, known as the Warburg effect. This metabolic phenotype enables tumor cells to rapidly generate ATP and produce a large amount of intermediate metabolites, such as phosphoenolpyruvate and lactate, providing carbon sources for nucleic acid, lipid, and protein synthesis [176]. In addition to abnormal glycolysis, tumor cells also exhibit an overall enhancement of anabolic metabolism, including increased glutamine catabolism to replenish tricarboxylic acid cycle intermediates, active de novo fatty acid synthesis to construct cell membrane structures, and significantly elevated nucleotide metabolic flux to support DNA replication and RNA transcription. Compared with normal cells, tumor cell metabolism has three notable differences: first, the shift in energy production from oxidative phosphorylation to glycolysis; second, the enhanced plasticity of metabolic networks to adapt to microenvironmental changes; and finally, metabolites not only serve as energy carriers but also act as signaling molecules involved in the regulation of cell proliferation and survival. These metabolic characteristics collectively form the metabolic basis of the malignant phenotype of tumor cells and provide potential targets for tumor diagnosis and treatment [177].

The upregulation of nucleotide synthesis metabolism in tumor cells is the material basis for their rapid proliferation [178], primarily achieved through the coordinated activation of de novo synthesis pathways and salvage synthesis pathways. The de novo synthesis pathway is significantly upregulated in tumor cells to meet the high demand for deoxynucleotides during DNA replication. Hypoxia‐inducible factor‐1α is activated under hypoxic conditions in the TME, enhancing the expression of IMPDH in the purine nucleotide de novo synthesis pathway while inhibiting mitochondrial respiratory chain function, thereby indirectly promoting the diversion of glycolytic intermediates toward nucleotide synthesis [179]. The salvage synthesis pathway recycles extracellular or catabolically produced free purine/pyrimidine bases, which are catalyzed by specific kinases to generate nucleotides, serving as a supplementary mechanism to the de novo synthesis pathway in tumor cells. When the de novo synthesis pathway is restricted (e.g., by chemotherapy intervention), tumor cells can maintain nucleotide levels by upregulating key enzymes in the salvage pathways, such as purine nucleoside phosphorylase (PNP) [180] and TK1 [181].

The upregulation of nucleotide synthesis metabolism not only provides the genetic material precursors necessary for tumor cell proliferation but also allows its metabolic intermediates (such as PRPP and IMP) to participate in cell growth regulation by activating signaling pathways like mTOR and AMPK, forming a malignant feedback loop in the metabolic‐signaling regulatory network [182]. The molecular mechanisms of this metabolic reprogramming involve multiple levels, including transcriptional regulation, enzyme activity modification, and metabolic network remodeling, serving as important metabolic markers for tumor cells to adapt to malignant proliferation.

### 3.2 The Key Enzymes in Nucleotide Metabolism in Tumors DNA Damage and Repair Mechanisms

#### 3.2.1 PARP‐1

PARP1, the inaugural member of the PARP family now consisting of 18 distinct members [183], primarily catalyzes the polymerization of ADP‐ribose units derived from the ADP donor NAD+ onto itself or other target proteins, resulting in the formation of linear or branched PAR polymers. It consists of three major domains: an amino‐terminal DNA‐binding domain composed of zinc finger motifs, a central auto‐modification domain containing BRCT motifs, and a highly conserved carboxy‐terminal catalytic domain [184, 185, 186]. These domains collectively mediate PARP1's response to various types of DNA damage.

PARP actively participates in multiple DNA repair pathways, contributing to the maintenance of genomic integrity [187, 188, 189]. It plays a pivotal role in the repair of DNA single‐strand breaks (SSBs) [190]. Upon the occurrence of SSBs in radiation‐damaged DNA, PARP's ZnF domain rapidly recognizes and binds to the break sites, recruiting repair proteins (e.g., XRCC1) to the damage sites, thereby facilitating SSB repair and enhancing cancer cell resistance to radiation [191]. Inhibition of the PARP‐coupled BER (base excision repair) pathway [30] triggers an accumulation of SSBs that escape repair, which subsequently impede advancing replication forks and give rise to DSBs [192]. Pharmacological PARP suppression amplifies the cytotoxic effects of radiation by disabling the ability of neoplastic cells to resolve DNA injury. Their mechanism involves competitively binding to the PARP active site with NAD+, thereby inhibiting PARP activity [193].

#### 3.2.2 RNR

The pivotal enzyme in de novo dNTP biosynthesis is RNR, which catalyzes the reduction of nucleoside diphosphates (NDPs) to deoxy‐NDPs. Nucleotide reductase reduces nucleotides to their corresponding deoxynucleotides [119, 194]. In mammals, RNR is a tetrameric enzyme composed of two homotetrameric subunits, ribonucleotide reductase catalytic subunit M1 (RRM1) and ribonucleotide reductase catalytic subunit M2 (RRM2). RRM1 is constitutively expressed throughout the cell cycle, whereas RRM2 expression is induced upon entry into the S phase [195]. Additionally, RRM2 undergoes rapid degradation via the proteasome in the G2 phase [196, 197]. RNR activity is rate‐limited by RRM2, which is therefore regarded as its catalytic bottleneck. Under conditions of DNA damage, p53‐dependent signaling drives the expression of the alternative subunit RRM2B [198]. Beyond its lack of involvement in cell cycle control, RRM2B contributes critically to de novo dNTP synthesis during stress conditions [199, 200, 201] and participates in mtDNA maintenance via both replication and repair mechanisms [202, 203, 204]. RNR is widely recognized for its link to cancer, ranking among the earliest proteins whose expression is triggered by DNA lesions [205]. In vivo, RRM2 overexpression acts in an oncogenic manner to promote pulmonary malignancy, while RRM1 confers protective effects against neoplastic transformation, cell motility, and distant colonization [116]. Accumulating evidence—including data from our own team—supports RRM2 as a valuable prognostic and diagnostic marker for a broad spectrum of cancers [206, 207, 208, 209]. However, the suitability of RRM1 and RRM2B as clinically applicable tumor biomarkers remains a matter of ongoing debate [116].

Several analogs effectively target this enzyme. Among them, gemcitabine diphosphate (dF‐dCDP) mimics the RNR substrate [210] and permanently inactivates the enzyme by forming a covalent bond with the large subunit RRM1 [211]. Fludarabine (2‐F‐ara‐A), clofarabine (Cl‐F‐ara‐A), and cladribine (2‐CdA)—metabolic products of purine nucleoside mimetics—are distinguished by their reversible inhibitory action against RNR. In this context, both the diphosphate and triphosphate forms of these analogs are implicated in inhibiting RNR by interacting with catalytic and allosteric sites, respectively. These interactions alter the quaternary structures of RNR and promote the formation of persistent RRM1 hexamers [212, 213, 214]. The dual inhibitory activity of these purine analogs and gemcitabine toward both RNR and DNA synthesis gives rise to a self‐reinforcing mechanism. By inhibiting de novo dNTP synthesis, these analogs reduce the levels of endogenous dNTPs, thereby facilitating their misincorporation into DNA. Thioguanine's antitumor activity is also attributable to the suppression of nucleotide biosynthesis, specifically through its metabolite methylthioguanine monophosphate, which inhibits the crucial purine biosynthesis enzyme phosphoribosyl pyrophosphate amidotransferase [215]. Nevertheless, the precise extent to which this metabolic blockade contributes to the drug's cytotoxic effects has yet to be elucidated [216], despite decades of evidence suggesting a contributory role [217].

#### 3.2.3 TS

Fluoropyrimidines—namely, 5‐FU, fluorodeoxyuridine, and the prodrug capecitabine—serve as irreversible inhibitors of TS [218]. TS utilizes folate 5,10‐methylenetetrahydrofolate (CH2THF) as a methyl donor to catalyze the reductive methylation of deoxyuridine monophosphate (duMP) to dTMP [219]. This reaction is the only way cells make dTMP from scratch, and it is a critical step that controls the overall pace of DNA synthesis [220]. The monophosphate form of fluoropyrimidine, 5‐F‐dUMP, acts as a substrate analog that competes with dUMP for binding to the catalytic site of TS, forming a stable ternary complex with the enzyme and CH2THF. This prevents the normal binding of the substrate, thereby inhibiting the enzyme [221], which explains why folate supplementation can enhance the efficacy of 5‐FU [222]. Trifluorothymidine (TFT) also stops TS from working, but first it needs TKs to add a phosphate group. It is in the same family as 5‐FU [223]. Inhibition of TS depletes intracellular dTMP, leading to a deficiency of dTTP, which is essential for DNA synthesis. This also disrupts the levels of other dNTPs due to various feedback mechanisms [224, 225]. Furthermore, the absence of TS activity prevents the reversal of these metabolites, resulting in an expansion of the dUMP pool. The nucleotides in this pool can be turned into dUTP and mistakenly get built into DNA. Fluoropyrimidines also have active triphosphate forms—5‐F‐dUTP and TFT‐TP—that do the same thing [226]. In fact, all three of these (dUTP, 5‐F‐dUTP, and TFT‐TP) look so much like dTTP that the replication machinery picks them up by mistake [227, 228]. A self‐reinforcing mechanism is evident. Unlike sugar‐modified analogs, fluoropyrimidines and uridines resemble thioguanine and decitabine in that their mere incorporation into DNA is not the source of cytotoxicity. Instead, the damage arises from the fluorouracil and TFT that remain in the genome, which promote mutagenic and lethal DNA lesions [229, 230]. Cells have evolved repair systems like mismatch repair and BER [30] to remove these unwanted DNA changes [231, 232]. BER includes several enzymes that are specially designed to recognize and remove uracil and thymine mismatches from the genome, including UNG, TDG, SMUG, and MDB4 [233, 234]. However, the intermediate products generated during BER can sometimes be more harmful to cells than the original DNA damage. This is especially true when these intermediates persist, which tends to happen when repair cycles become futile due to a large supply of fluorouracil in the cell, ultimately resulting in DNA breakage [230]. Beyond inhibiting TS and integrating into DNA, fluoropyrimidine nucleotide triphosphates can also get incorporated into newly made RNA molecules. This insertion disrupts essential cellular functions like ribosomal RNA processing and mRNA splicing [235, 236]. The protective effect of high‐dose uridine therapy against 5‐FU toxicity further confirms how important RNA incorporation is to the mechanism of fluoropyrimidines [237, 238]. Indeed, uridine has been approved by the Food and Drug Administration (FDA) as a rescue therapy for 5‐FU or capecitabine overdose or severe toxicity [239].

#### 3.2.4 HGPRT

HGPRT is the enzyme that converts the thiopurine drugs 6‐MP and 6‐TG into their active monophosphate forms, known as 6‐thioinosine monophosphate (TIMP) and 6‐thioguanosine monophosphate (TGMP). The HPRT1 gene provides the instructions for making this enzyme. TIMP can subsequently undergo metabolism into purine monophosphate (TXMP) via IMPDH, while TGMP is processed by GMP synthetase (GMPS) [240]. These nucleotide analogs subsequently compete with natural guanine for incorporation into the DNA double strand as DNA [43]. Approximately one in every 6000 nucleotides is substituted with thioguanine [241]. Once incorporated into DNA, thioguanine residues are prone to random methylation, which predisposes them to pair with thymine rather than cytosine [241]. The repair system recognizes these TG·T mismatches through MutS homolog 6, but the location of the aberrant base on the template strand converts this recognition into a lethal event, ultimately triggering DNA breakage and apoptosis [216]. Lower rates of disease recurrence are observed in patients with higher DNA‐TG content [241, 242]. Beyond these well‐characterized effects, the thiopurine metabolic network produces multiple additional intermediates, some of which also exert antileukemic actions. For instance, methylated derivatives of thioinosine nucleotides directly block the de novo biosynthetic route for purine nucleotides [216, 243].

A study conducted in pediatric patients in the Netherlands revealed that reduced HGPRT activity correlated with a less favorable prognosis in precursor B‐cell acute lymphoblastic leukemia (pre‐B‐ALL), albeit without establishing a link to in vitro resistance to 6‐TG [244]. Another investigation hinted that decreased expression levels of HGPRT might hypothetically be associated with poorer treatment outcomes in ALL [245]. It has been reported or suggested that allopurinol, an inhibitor of XO, has the potential to augment HGPRT activity in individuals with inflammatory bowel disease (IBD) and ALL [246, 247]. Acquired HPRT1 mutations have been identified in 6‐TG‐resistant clones of the HL‐60 myeloid cell line [248]. Currently, there is limited understanding of the function of IMPDH in thiopurine resistance. Azathioprine is a prodrug that gets converted to 6‐MP outside cells. In one IBD patient, a mutation in the IMPDH1 promoter weakened transcription and affected the patient's response to the drug [249]. This case implies that cancer patients on thiopurine therapy might become resistant if IMPDH activity is reduced or lost. GMPS is downregulated in T‐cell variants that have developed resistance to thiopurines relative to the original cell lines [250], yet the clinical implications of GMPS alterations warrant further exploration.

#### 3.2.5 Nucleoside Monophosphate Kinase

The enzyme deoxycytidine kinase (dCK) serves as the primary kinase responsible for activating pyrimidine and purine nucleoside analogs. However, adenosine kinase has also been reported to play a minor role in the phosphorylation of purine nucleosides, such as ara‐A, although its significance has been subject to debate [251, 252]. In vitro studies using cell lines and patient‐derived primary mantle cell lymphoma cells have shown that diminished dCK activity results in resistance to ara‐C along with 2‐F‐ara‐A, dF‐dC, and 2‐CdA, without affecting nonantimetabolite cytotoxic drugs [253]. An independent investigation later confirmed these observations [254]. A separate study reported that breast cancer cell lines exhibit similar cytotoxic responses to decitabine and dF‐dC. Intriguingly, the same study observed that dCK levels are elevated in breast cancer tissues of patients with poor outcomes relative to those with better clinical outcomes [255]. Therefore, while downregulation of dCK may confer resistance to nucleoside analogs in vitro, its clinical relevance may be limited due to its association with reduced cell growth and proliferative ability in the physiological setting. This is supported by evidence suggesting that lower dCK level may increase sensitivity toward corticosteroid treatment in AML, in vitro and in clinical settings [256, 257]. Additionally, the lack of DCK mutations among patients with relapsed or refractory AML highlights that dCK downregulation in drug‐resistant cell lines is sufficient to explain clinical resistance [258]. Nevertheless, earlier work suggested that alternative splicing may contribute to diminished dCK activity in AML patients with acquired resistance [259]. In that same study, cell lines established before and after high‐dose ara‐C treatment showed that two resistant clones had reduced dCK expression following treatment [260]. Among seven ara‐C‐resistant AML patients, five exhibited low dCK activity. Polymorphisms in DCK may also be associated with prognosis in pediatric AML [261] and toxicity to ara‐C in pediatric ALL [262]. In pediatric AML regimens, etoposide is a topoisomerase II inhibitor that is commonly given alongside ara‐C and purines. Studies have shown that this drug can boost dCK activity [263].

DGK, which is encoded by the DGUOK gene, may play a minor role in mediating the cytotoxicity of purine nucleoside analogs. This is exemplified by Cl‐F‐ara‐A in cells lacking dCK [264], with simultaneous reduced dCK and dGK activity observed in cell lines with ara‐G resistance [265]. Overexpression experiments with dGK for 2‐CdA and ara‐G further support this notion [266]. Mitochondrially localized DGK has emerged as a potential key player in the S‐phase‐independent pharmacological activity of Cl‐F‐ara‐A [267].

The activation of thymidine nucleoside analogs depends on cytosolic TK1. In colorectal cancer cells, silencing of TK1 renders cells resistant to trifluridine while preserving their susceptibility to 5‐FU [268]. While TK1 is crucial for phosphorylating 5‐FdU, which is a 5‐FU derivative [269], this step can be circumvented via RNR‐mediated conversion of 5‐FUDP to 5‐FdUDP [270]. However, the role of TK2 in mediating response to nucleoside analog therapy is still uncertain. In this context, TK2‐inhibiting nucleoside analogs have emerged as promising S‐phase‐independent anticancer agents [271].

One way to get around resistance caused by a failure to add the first phosphate group is to supply the phosphorylated form of nucleoside analogs directly to the cell interior. This strategy is complicated by the fact that phosphate‐containing drugs are highly polar and cannot readily cross the lipid membrane because the required transporters are absent. Chemical modification of the drug thus becomes necessary to allow membrane permeation. This approach is analogous to the tactics developed for circumventing nucleoside analog import defects described earlier. A class of prodrugs known as ProTides has been designed to deliver monophosphorylated nucleoside analogs into cells. These compounds contain a propanoate triester group that facilitates their passage across the plasma membrane without requiring transport proteins, and they bypass the usual dependence on dCK to initiate drug activation [272, 273]. This approach has already been applied to antiviral agents that are now being tested in clinical studies [274]. Analogs of this technology have produced phosphoramidate derivatives of ara‐C and dF‐dC, which have undergone preclinical evaluation. Among these, the dF‐dC derivative has reached Phase I/II clinical investigation [275, 276]. Ara‐C‐resistant mouse L1210 cells, which have lost dCK expression, remain sensitive to the prodrug of ara‐C [277], demonstrating the conceptual validity of this approach. Other medicinal chemistry strategies exist for lipidated nucleoside analog monophosphates [278], diphosphates, and triphosphates, but they have not yet entered clinical trials due to various challenges [279].

#### 3.2.6 NDP Kinase

Cytidine and uridine analog monophosphates are phosphorylated by UMP/CMP kinase [280], a protein whose synthesis is directed by the CMPK1 gene [281, 282]. Studies examining mRNA expression in colorectal cancer tissues exhibiting clinical resistance to 5‐FU suggest that reduced UCK levels may constitute a mechanism of fluoropyrimidine resistance [283]. Additionally, an analysis of 80 xenograft models has indicated that CMPK1 expression could serve as a predictor of 5‐FU sensitivity [284]. Polymorphisms within the CMPK1 gene have been linked to overall survival in studies involving 102 patients with pancreatic cancer and lung cancer who were treated with dF‐dC [285, 286]. Therefore, assessing the status of CMPK1 could provide valuable insights for personalized metabolite therapy.

Moreover, mitochondrial UMP/CMP kinase 2, another monophosphate kinase, possesses the capability to phosphorylate dF‐dCMP, albeit its precise role in chemotherapy remains to be fully elucidated [287]. Beyond its involvement in the phosphorylation of TFT monophosphate [288], the role of TMP kinase in nucleoside analog therapy remains largely unknown, as its expression, unlike that of CMPK1, does not independently correlate with 5‐FU sensitivity in xenograft models [284]. Intriguingly, the monophosphate kinase responsible for generating diphosphates of purine nucleoside analogs has yet to be identified or further characterized.

#### 3.2.7 Nucleoside Triphosphate Kinase

The NDP kinases 1 and 2 (NDPK1/2), encoded by the genes NME1/2, are responsible for catalyzing the final phosphorylation step, ultimately yielding the active triphosphate forms of nearly all clinically utilized nucleobases and nucleoside analogs. While polymorphism in NME1 has been linked to toxicity in AML patients undergoing treatment with ara‐C, a study involving a cohort of 360 Caucasian patients failed to uncover any correlation between NME1 polymorphism and efficacy measures [289]. Similarly, a study on Chinese AML patients did not identify a significant association between single nucleotide polymorphisms (SNPs) in NME1 and clinical response. However, it did reveal a notable correlation between SNPs in NME2 and complete response [290]. The interpretation of these findings is intricate, as prior reports have indicated that NME1 expression is associated with a poor prognosis in AML [291, 292], whereas NDPK2 exhibits tumor suppressor functions [293].

### 3.3 The Key Enzymes in Nucleotide Metabolism in Tumors Oxidative Stress

**FIGURE 3:**
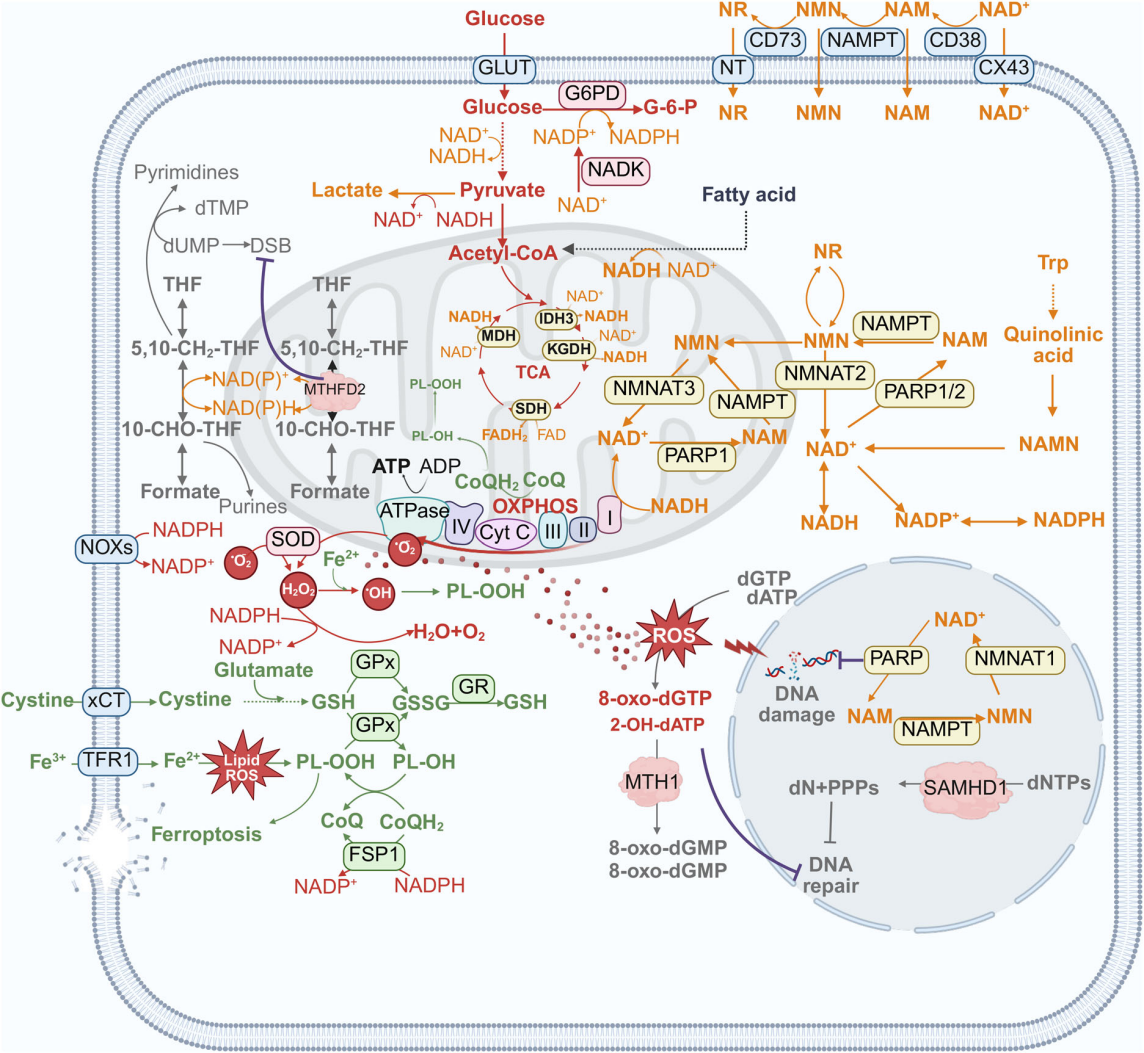
Regulation of oxidative stress response and metabolic pathways by nucleotide metabolizing enzymes. This figure delineates the intricate network of oxidative stress responses within the cell, highlighting the regulatory roles of nucleotide‐metabolizing enzymes in cellular metabolism. The yellow pathways represent reduction reactions, the red pathways indicate oxidation processes, and the green pathways are associated with ferroptosis. Created with BioRender.com.

Elevated ROS levels are a common feature of many cancers [54], often arising from disrupted redox homeostasis [294]. This buildup of ROS can inflict oxidative injury on DNA and proteins, potentially leading to mutagenesis or apoptotic cell death [295]. The ubiquitous upregulation of antioxidant defenses observed in cancer may be attributed to these high ROS levels [296], which has inspired strategies aimed at targeting elevated ROS for anticancer therapy [297, 298, 299, 300, 301, 302, 303] (Figure 3).

#### 3.3.1 MTH1

MTH1, the human ortholog of bacterial MutT and also termed NUDT1, functions in oxidative stress defense. This enzyme hydrolyzes oxidized nucleotides within the dNTP pool, such as 2‐OH‐dATP and 8‐oxo‐dGTP, thereby blocking the insertion of damaged nucleobases into DNA, which could otherwise lead to mutations [304, 305]. The dependence of lung cancer on functional MTH1 was initially established in Ogg1‐knockout mice. These animals lack the oxidative DNA repair enzyme OGG1, yet tumor development only occurs when MTH1 is active. This conclusion is reinforced by the observation that simultaneous deletion of both Ogg1 and Mth1 confers complete resistance to lung tumorigenesis [306], thus validating MTH1 as a druggable target in living organisms. Clinically relevant links have been drawn between MTH1 and oxidative DNA damage across a variety of disorders [307]. Tumor tissues frequently exhibit pronounced upregulation of MTH1 [308, 309, 310], and the protein's catalytic activity shows a positive correlation with 8‐oxo‐dGTPase levels in neoplastic cells [311, 312]. In addition, MTH1 functions to protect cancer cells from undergoing senescence when faced with oxidative stress [313, 314, 315].

The initial mechanistic insight linking MTH1 to cancer therapy came from Rai and colleagues at the Weinberg laboratory, who demonstrated that MTH1 is essential for preventing cancer cell senescence [313]. Since then, potent MTH1 inhibitors with anticancer activity have been developed [316, 317], sparking widespread attention to this enzyme as a viable target for cancer intervention. A range of MTH1 inhibitors with differential cytotoxic activity against tumors have been generated as a result of these observations. One of these compounds, TH1579, which is synonymous with karonudib and OXC‐101 [318], has entered clinical testing for both solid tumors (NCT03036228) and blood cancers (NCT04077307). The antitumor mechanisms of the first‐generation inhibitor TH588 and its more advanced derivative TH1579 are now clearly defined. Both molecules operate by blocking MTH1 catalysis and disrupting microtubule assembly, which together confer robust anticancer efficacy. In vitro exposure to these agents blocks cell cycle progression at mitosis and triggers the spindle checkpoint [319], prompting ROS accumulation. The resulting oxidative environment converts dGTP to 8‐oxo‐dGTP, which is then misincorporated into genomic DNA during replication, ultimately culminating in cancer cell death [320, 321].

#### 3.3.2 MTHFD2

MTHFD2 is active during embryonic development, turned off in mature tissues, and reactivated in malignant cells [322], positioning this enzyme as an attractive candidate for anticancer drug development. Within mitochondria, one‐carbon units are derived from serine and are carried by THF as methylene‐THF. This intermediate undergoes oxidation to formate, which is then exported to the cytoplasm [323, 324]. Once in the cytoplasm, formate enters pathways for purine, thymidylate, and methionine biosynthesis. The cytoplasmic oxidation of methylene‐THF depends entirely on the trifunctional NADP‐dependent enzyme MTHFD1, which possesses dehydrogenase, cyclohydrolase, and synthetase activities. In mitochondria, however, these same steps are performed by two separate enzymes: the bifunctional NAD‐dependent MTHFD2L, which handles dehydrogenase and cyclohydrolase functions, and the monofunctional MTHFD1L, which provides synthetase activity. In embryonic and transformed cells, the dehydrogenase and cyclohydrolase tasks in mitochondria are taken over by MTHFD2, reflecting a shift from MTHFD2L to MTHFD2 during oncogenic transformation [325, 326, 327].

MTHFD2 ranks among the metabolic enzymes most strongly overexpressed in malignancies [328], attracting considerable attention as a candidate for anticancer therapy. This enzyme contributes to the de novo production of thymidylate (dTMP) through the supply of methylene‐THF (CH2‐THF). Consequently, inactivation of MTHFD2 reduces dTTP levels, resulting in replication stress due to thymine deficiency. Reduced dTMP production simultaneously triggers accumulation of the TYMS substrate dUMP. Following phosphorylation, this metabolite gives rise to dUTP. When dUTP is heavily misincorporated into DNA, the resulting increase in genomic uracil aggravates replication stress and ultimately causing strand breakage and cell killing. MTHFD2 inhibitors are primarily divided into two series: DS18561882 [329] and TH9619 [330]. Multiple studies confirm that knocking down MTHFD2 with RNA interference efficiently eliminates most tumor cells. This phenotype is specifically attributable to MTHFD2 silencing, as restoration of the enzyme with an RNAi‐refractory mutant rescues cell viability [330]. Interestingly, inactivation of MTHFD2 via CRISPR–Cas9 yields a markedly different picture [331], in which tumor cells maintain their survival by exploiting the SHMT1 pathway [280].

#### 3.3.3 SAMHD1

SAMHD1 was initially described as DCIP, a protein induced by IFN‐gamma in dendritic cells, when it was recognized as the human counterpart of the mouse Mg11 gene in a dendritic cell cDNA library [332]. The enzyme's structural organization, determined in later investigations, features an N‐terminal SAM domain paired with a central HD domain containing conserved histidine [333] and aspartic acid [30]. Its capacity to hydrolyze the triphosphate group from dNTPs was subsequently documented [80, 81]. SAMHD1 is a representative of the HD domain superfamily, whose members are characterized by metal‐dependent phosphohydrolase function [334]. The catalytic reaction involves cleavage of the alpha‐phosphate bond of dNTPs, producing deoxynucleosides and inorganic triphosphate as products. DNA polymerases cannot utilize dNTPs that lack the triphosphate group for chain elongation, so SAMHD1 effectively excludes these molecules from participating in DNA synthesis by removing that required group. The activity level of the enzyme is governed by the prevailing nucleotide environment [120, 335, 336, 337]. All four canonical dNTPs and dUTP can be hydrolyzed by SAMHD1 [338], consistent with SAMHD1 being the primary regulator of the dNTP pool in human cells [339].

The composition of the dNTP pool is intimately connected to genomic stability, making SAMHD1 a significant player in cancer biology [340, 341]. Boosting the performance of standard antimetabolite therapies, especially nucleoside and nucleobase analogues, represents a major approach to exploiting SAMHD1 in cancer treatment [342]. By functioning as a triphosphohydrolase, SAMHD1 can revert the pharmacologically active triphosphate metabolites of nucleoside analogues to their inert prodrug counterparts [342]. The first evidence that nucleoside analogues are subject to SAMHD1‐mediated processing came from studies on clofarabine, whose active form Cl‐F‐ara‐ATP also acts as an AS2 agonist [343]. A growing list of nucleoside analogue metabolites have subsequently been confirmed as SAMHD1 substrates [344, 345, 346]. At present, therapeutic targeting of SAMHD1 relies largely on inducing its proteasomal breakdown, a tactic that can be augmented by Vpx delivery to selectively degrade the enzyme in cancer cells and potentiate ara‐C activity against AML [106]. Although several small‐molecule SAMHD1 catalytic inhibitors have been identified using recombinant protein in vitro, none have yet shown activity in cellular contexts [343, 347, 348, 349]. Additionally, deoxyguanosine and its structural relatives, including acyclovir, have been found to suppress SAMHD1 in vitro, suggesting a path forward for the design of novel chemical probes [347]. Indirect pharmacological strategies, for instance ribonucleotide reductase inhibitors, can inhibit SAMHD1‐mediated resistance in cancer cells by disrupting nucleotide metabolism [350].

#### 3.3.4 NAD+

NAD+ is essential for cellular homeostasis. The maintenance of cellular homeostasis relies heavily on NAD+, which cycles between its oxidized and reduced forms to facilitate electron transfer in diverse metabolic routes. These include glycolysis, fatty acid oxidation, the TCA cycle, oxidative phosphorylation, and the synthesis of serine [351, 352]. NAD+ also has functions beyond metabolism, acting as a signaling molecule that enables protein deacetylation through Sirtuins and supports ADP‐ribosylation via PARP proteins when DNA lesions occur. NAD+ is synthesized through the combination of adenosine monophosphate [34] and NMN. The hydroxyl group of AMP can be phosphorylated to produce NADP+ along with the release of a phosphate group. NMN, composed of a ribose ring, a phosphate group, and nicotinamide [286], is responsible for the redox function of NAD+. The carbon atom of NAM can accept hydrides, converting it into the reduced form, NADH. NAD+ not only participates in redox reactions but also donates ADP‐ribose to various enzymes, a process that yields nicotinamide as a metabolic byproduct.

*NAD+ in cancer metabolism*. According to the Warburg effect [353], cancer cells primarily rely on glycolysis for energy production, rather than OXPHOS, even under conditions of normal mitochondrial function and oxygen availability [354, 355, 356]. While the OXPHOS pathway can generate 36 moles of ATP per glucose molecule, cancer cells utilizing glycolysis produce only two ATP molecules per glucose molecule [357, 358]. Given the limited availability of nutrients, increased NAD+ levels are essential to support the high glycolytic demands of cancer cells. Pathways such as the serine synthesis, PPP, and fatty acid synthesis are upregulated in cancer, and glutaminolysis is also enhanced to serve as a primary nitrogen source. NAD+/NADH/NADP+/NADPH serve as essential cofactors for these pathways, which are crucial for facilitating rapid energy generation, counteracting reactive oxygen species [54] production, and promoting tumor proliferation [359].

Inhibition of nicotinamide phosphoribosyltransferase (NAMPT) can lead to the reduction of NAD+, subsequently inhibiting ATP synthesis [360]. Cancer cell growth suppression and death induction represent the ultimate effects of this process. These observations have positioned NAMPT as a clinically relevant drug target. A number of selective NAMPT inhibitors have entered clinical evaluation, with Phase II trials ongoing for several candidates [357, 360, 361, 362]. The most advanced in terms of characterization is FK866/APO866, a noncompetitive inhibitor that elicits substantial anticancer activity. Its suppression of NAMPT translates into decreased glucose consumption, reduced ATP generation, impaired fatty acid synthesis, and ROS accumulation. NAD+ depletion downstream of NAMPT inhibition further disrupts sirtuin and PARP function [362]. Importantly, FK866 leaves mitochondrial NAD+ untouched, which selectively sensitizes malignant cells to its action [361]. Moreover, FK866 is particularly potent against NAMPT‐overexpressing tumors that depend heavily on glycolysis [362, 363]. KPT‐9274, a second‐generation oral inhibitor, drives apoptosis and produces DNA lesions in vitro [364], while significantly attenuating tumor growth in various models [365], including pancreatic neuroendocrine tumors [366], triple‐negative breast cancer [367], and leukemia [368, 369]. This drug has progressed to Phase I assessment for advanced solid malignancies and non‐Hodgkin's lymphoma (NCT02702492) [370, 371]. OT‐82, a distinct NAMPT inhibitor, exhibits preferential activity toward hematologic versus solid tumors. In preclinical safety studies, OT‐82 was well tolerated in mice and nonhuman primates, with no observed neurologic, cardiac, or retinal toxicity [372], and has advanced to Phase I testing for relapsed or refractory lymphomas (NCT03921879). GMX1777 and GMX1778 represent additional small‐molecule NAMPT inhibitors with efficacy analogous to FK866. However, the development of numerous other NAMPT inhibitors has been discontinued owing to excessive toxicity.

#### 3.3.5 IMPDH

IMPDH in the cytoplasm oxidizes IMP to xanthosine monophosphate, representing a NAD+‐dependent rate‐determining step in the de novo synthesis of GTP [373]. GTP functions not only as a classic second messenger governing diverse cellular processes but also as an energy source for protein translation, and its levels are frequently elevated across various malignancies [374]. The salvage enzyme HPRT, though capable of recovering guanine, cannot compensate for the massive nucleotide drain imposed by aggressive tumor growth. To overcome this limitation, cancer cells drive GTP production through upregulation of IMPDH, ensuring an adequate supply of precursors for DNA and RNA synthesis during rapid expansion.

A variety of malignancies, ovarian cancer included, display aberrantly high IMPDH expression [375]. IMPDH2 is the predominant isoform upregulated in human tumors, in contrast to IMPDH1, which remains relatively unaffected [376, 377, 378, 379, 380]. In ovarian cancer, IMPDH2 overexpression has been linked to diverse pathological subtypes, shorter survival periods, and more advanced tumor staging, all of which collectively indicate a worse prognosis [381]. The oncogenic functions of IMPDH2 are mediated through its activation of the PI3K/Akt and Wnt/β‐catenin pathways, which propel tumor progression and trigger the epithelial–mesenchymal transition program [382]. Given its dramatically elevated activity, IMPDH—and particularly the IMPDH2 isoform—has emerged as a promising candidate for the development of anticancer agents.

In fact, IMPDH inhibitors have a long history of use as antitumor drugs [383]. Evidence from multiple studies indicates that IMPDH inhibitors trigger apoptosis through several distinct pathways. These include MEK/ERK pathway inactivation that suppresses Bcl‐2 [384], combined inhibition of Src/PI3K/Akt and mTOR that promotes Bax/Bak activation, disruption of mTORC1 or c‐Myc signaling that diminishes ribosomal RNA biogenesis [374, 385, 386], and mitochondrial pathway engagement that ultimately leads to cell death [387]. In ovarian cancer, Look and colleagues observed effective IMPDH suppression by the tizofurin metabolite thiazole‐4‐amide adenine dinucleotide [375]. Likewise, benzamide riboside exerts its apoptotic effects in ovarian cancer cells by blocking c‐Myc and its downstream regulator Cdc25A, which results in cell cycle blockade [388, 389]. Nevertheless, this phenomenon has been documented in only a limited number of ovarian cancer cell lines, and the molecular basis of apoptosis triggered by IMPDH inhibition is yet to be fully elucidated. From a clinical perspective, the utility of IMPDH inhibitors is hampered by inconsistent efficacy, high‐dose toxicity, and intertumoral variation in IMPDH abundance [383]. Currently, exploring IMPDH as an immunosuppressor or biomarker still holds great value, which may present an opportunity to revitalize its use as an antitumor drug.

### 3.4 Tumor Treatment Strategy Based on Nucleotide Metabolism

#### 3.4.1 Adenosine‐Targeting ROS‐Reactive Ectonucleotidase Inhibitor Therapy

It has been observed that the evasion of tumors from immune destruction is linked to the production of immunosuppressive adenosine within the TME. Anticancer therapies can elicit the release of ATP from tumor cells, prompting the swift conversion of ATP into adenosine by the ectonucleotidases CD39 and CD73, thereby intensifying immune suppression within the TME [390]. Researchers have devised a combined therapeutic strategy that not only stimulates the release of ATP to induce immunogenic cell death (ICD) but also concurrently restricts the degradation of ATP into adenosine, thus stimulating antitumor immunity and ultimately sustaining an antitumor immune response.

This study entails the creation of nanoparticles that generate reactive oxygen species [54] through electronic interactions and phenylboronic ester linkages. These nanoparticles encapsulate the ectonucleotidase inhibitor ARL67156 [391]. Under infrared radiation, the nanoparticles produce ROS, which triggers the release of ATP from MOC1 cancer cells in vitro. This event subsequently triggers the breakdown of the phenylboronic ester and the liberation of ARL67156 from the nanoparticle carrier, which in turn blocks the degradation of ATP to adenosine and strengthens the antitumor immune response.

ICD was induced in both human and mouse tumor cells, accompanied by elevated release of extracellular ATP (eATP), CRT, and HMGB1. These molecules facilitate tumor antigen presentation and T‐cell activation. In an oral cancer mouse model, the nanoparticles successfully delivered IR700 and ARL to the tumor site. Under light irradiation, the tumor cells were rapidly eliminated. Furthermore, the CD8+/T regulatory cells (Treg) ratio in the immunogenic TME increased, resulting in systemic and durable tumor responses across MOC1, MOC2, MC38, and 4T1 tumor models. Additionally, tumor‐specific T‐cell responses were induced in these models. When combined with PD1 blockade, these responses elicited even stronger antitumor effects.

In PDOTS models, NP700‐ARL (+) treatment resulted in phototoxic tumor cell killing. Furthermore, it overcame PDT‐driven immune suppression by reinstating T‐cell proliferation, thereby reinforcing the immune system's ability to fight tumors. The findings of this study indicate that the utilization of nanoparticles as a therapeutic approach for human cancers holds significant translational potential.

#### 3.4.2 ATP‐Targeting Aptamer Therapy

ATP, serving as the primary energy source in organisms, sustains cellular metabolism by participating in processes such as DNA synthesis and glycolysis [392, 393]. ATP also functions as an intracellular or extracellular messenger molecule, involved in the pathophysiological processes of numerous diseases [394, 395]. Altered ATP levels and localization are linked to various diseases, including cancer and inflammation‐related disorders. Intracellular ATP is primarily produced through oxidative phosphorylation in mitochondria [396]. Imbalances in mitochondrial ATP levels signify mitochondrial dysfunction, which can be regarded as a hallmark of tumorigenesis. Studies have shown that, compared with normal tissue, the eATP concentration in tumor tissue is much higher (10^3^ to 10^4^ times) [397, 398]. This eATP not only fuels tumor growth and progression by shaping the TME but also reprograms cellular immune responses by targeting specific cellular receptors [399]. Furthermore, cancer cells have been shown to release ATP into the extracellular milieu during autophagy and apoptotic events [400]. Overall, the spatial and temporal dynamics of ATP exert extensive effects on cellular and biochemical reactions, significantly influencing metabolic activities in both physiological and pathological states. Currently, many studies integrate aptamer probes with advanced functional nanomaterials, enabling high‐resolution ATP detection and visualization across scales from subcellular organelles to whole organisms.

Short DNA or RNA strands known as aptamers are produced through SELEX, an in vitro selection methodology. Their exceptional target recognition properties and synthetic accessibility have positioned aptamer‐based sensors as versatile platforms for detecting a wide range of targets—including ions, small molecules, proteins, and cells—in combination with signal transduction approaches [401, 402, 403]. The identification of a high‐affinity ATP aptamer by Huizenga and Szostak in 1995 marked a significant milestone in the application of aptamers to ATP analysis [404]. Building on this work, Li and coworkers developed structure‐switching aptamer sensors for ATP in 2003, an innovation that spurred further developments in the field [405, 406, 407, 408, 409, 410]. A key limitation of applying these probes to intracellular ATP detection lies in their polyanionic nature, which prevents them from crossing biological membranes independently. This barrier has been surmounted through the use of nanomaterial delivery systems that shuttle DNA probes into live cells for imaging applications [405]. The convergence of aptamer technology with nanomaterial engineering has consequently given rise to a range of nanosensors designed for spatiotemporal ATP imaging in living cells [411, 412, 413].

One well‐known example of aptamers used as targeting ligands is AS1411 [414]. This DNA aptamer specifically recognizes the protein nucleolin, whose expression is markedly elevated in breast cancer and glioblastoma [415, 416]. AS1411 has also been frequently employed as a targeting element in diverse nanocarrier systems, including lipid‐based and gold nanoparticle formulations [417, 418]. In a recent report, Liu and coworkers created a bispecific RNA‐α‐polyamide chimera that simultaneously recognizes CD44 and EpCAM using two double‐stranded RNA aptamers [419]. The resulting chimera exhibited a notably extended half‐life in the bloodstream, which enabled it to suppress tumor cell growth with greater efficiency. Shigdar et al. reported another notable example showcasing the great potential of aptamer chimeras [420]. Delivery of EpCAM aptamers to the brain presented a formidable obstacle, which the authors overcame by incorporating a second aptamer specific for the transferrin receptor to drive transcytosis across the endothelial barrier. This strategy was extended in subsequent work to create an aptamer‐based chimera for targeted doxorubicin delivery and ATP‐triggered cytoplasmic release. The design joins the AS1411 aptamer, which homes to nucleolin‐expressing cancer cells, with an ATP‐binding aptamer that functions as both a drug docking station and a stimulus‐sensitive releasing element. Construction of the chimera involved annealing the ATP aptamer to its complementary strand, which was attached to the AS1411 moiety via a thymidine linker. The double‐stranded segment thus formed served as the loading site for doxorubicin. Upon exposure to intracellular ATP, the chimera readily discharged its doxorubicin cargo, as confirmed by functional testing. More broadly, this work illustrates the flexibility of aptamer‐based platforms in formulating combination regimens with heightened selectivity and pharmacological efficacy [421].

The STING signaling pathway occupies a pivotal role in immune therapies [422]. Activation of this pathway amplifies tumor immunogenicity by reshaping the immunosuppressive TME and augmenting lymphocyte infiltration into tumor tissue [423, 424]. Endogenous activation of the STING signaling pathway in tumor cells triggers ICD, further elevating tumor immunogenicity. cDNs (including 2′,3′‐cGAMP, CDG, CDA, and 3′,3′‐cGAMP) serve as natural ligands and agonists for STING [425]. Upon binding to STING, CDNs activate the STING protein and downstream signaling cascades, resulting in the expression of IFN‐I [426, 427, 428]. This, in turn, induces the maturation and activation of dendritic cells (DCs) and stimulates T‐cell immune responses. However, CDNs are susceptible to degradation by phosphodiesterases, and their low cellular membrane permeability and limited druggability pose significant challenges for clinical application. Given that the cGAS–STING signal is a crucial nexus between radiotherapy and immunotherapy, targeting STING in combination with ionizing radiation [54] holds immense potential for achieving synergistic antitumor immune effects. Recently, various STING agonists have embarked on clinical trials, laying the groundwork for combination therapies [429, 430].

In clinical immunotherapy, the targeted delivery of CDN, a STING ligand drug, is hampered by the delivery system. The high negative charge and instability of CDNs render current delivery systems ineffective, severely impeding their clinical utility. Therefore, the development of novel and efficient delivery systems for CDNs to enhance tumor treatment outcomes has emerged as a focal area of research [431]. An inhalable liposome formulation coated with phosphatidylserine and loaded with the natural CDN compound cGAMP was developed, which was administered in conjunction with fractionated X‐ray irradiation for melanoma B16‐OVA. The dual treatment strategy was the most potent, driving tumor‐specific systemic immunity and eradicating distant B16‐OVA lesions that were not directly irradiated [432]. Parallel work demonstrated that polymer‐based delivery of STING agonists in conjunction with low‐dose fractionated infrared light also produced strong antitumor immune responses [433]. Although liposomes can effectively target tumor endothelial cells and induce acute tumor necrosis, CDNs can only be efficiently delivered to cancer cells via nanodiscs. A CDN prodrug with a diproline peptide linker was attached to thiol‐terminated polyethylene glycol (PEG)–phospholipid, and the resultant CDN–PEG–lipid was internalized by cells and cleaved by endosomal peptidases to release the STING agonist. Loading CDN onto nanodiscs significantly promoted tumor penetration and achieved a high therapeutic effect after a single administration, accompanied by minimal and transient toxicity. Across three distinct tumor models, LND–CDN eradicated 80% of tumors in the MC38 model and extended median survival by 50% in the 4T1 breast cancer model [434]. This study introduced a delivery vehicle comprising PEG–phospholipid‐modified liposomes self‐assembled to present CDNs. With high mobility and plasticity, this vehicle efficiently penetrates tumor tissues, enhancing CDN delivery and tumor treatment efficacy.

Despite the extensive use of nanomaterials in designing cytoplasmic delivery systems for CDNs, issues such as low loading capacity, inadequate release, complex composition, and potential biosafety risks have somewhat hindered the clinical translation of CDN cytoplasmic delivery systems. Consequently, researchers devised a nanocarrier‐free CDN cytoplasmic delivery strategy, where CDNs containing guanine [30] bases oligomerize in the presence of excess potassium (K+) and further self‐assemble with multivalent metal ions to form CDN nanoparticles (CDN NPs). CDN NPs enable cytoplasmic delivery of CDNs without the need for nanocarriers and can bolster tumor immunotherapy through various mechanisms, including the modification of the immunosuppressive TME, induction of ICD in tumors, augmentation of tumor‐infiltrating lymphocyte proportions, and elicitation of tumor antigen‐specific T‐cell responses. The combination of CDN NPs with the immune checkpoint inhibitor (ICI) anti‐PD‐1 can further amplify the immune therapeutic effects on solid tumors. This strategy circumvents the use of nanocarriers, boasts a straightforward and efficient preparation process, and holds promising prospects in tumor immunotherapy. The nanocarrier‐free CDN cytoplasmic delivery strategy enhances CDN cellular uptake efficiency and has demonstrated remarkable immune therapeutic effects in tumor immunotherapy, exhibiting broad clinical application potential. It also provides a crucial reference for the design of carrier‐free CDN cytoplasmic delivery (or self‐delivery of CDNs) strategies [435].

Manganese (Mn^2^^+^) is an essential micronutrient for human physiology and has been identified as a STING pathway agonist via cGAS activation [436]. Mn^2^^+^ not only directly activates cGAS but also enhances its sensitivity in recognizing cytoplasmic DNA, thus markedly lowering the threshold concentration of double‐stranded DNA (dsDNA) required for cyclic GMP–AMP (cGAMP) synthesis. Furthermore, Mn^2^^+^ enhances STING activation by augmenting the target affinity between cGAMP and STING [437]. Based on these findings, investigators have developed an alginate (Alg)–Mn material capable of sustainably releasing Mn^2^^+^ and working synergistically with radiotherapy. The coordinated release of Mn^2^^+^ is timed to coincide with the buildup of radiation‐induced DNA lesions, enabling the two therapeutic components to synergistically activate the cGAS–STING pathway in tumor cells. Findings from animal models confirm that Alg–Mn combined with radiotherapy produces significantly greater antitumor effects than either approach alone. In parallel, the combination group showed clear signs of dendritic cell maturation, enhanced T‐cell responsiveness, and robust immune infiltration, underscoring the ability of this dual strategy to reshape the TME into an immune‐active state [438]. Another research team combined synthetic polyphenols with radiosensitizers and Mn^2^^+^ to generate a metal‐phenolic network responsive to the acidic TME. This nano‐platform, in conjunction with X‐ray irradiation, also effectively activated the cGAS–STING signal [439]. Evidence for the potent antitumor immunity induced by STING agonist and radiosensitizer combination therapy came from robust inflammatory cytokine secretion, pronounced CD8^+^ T‐cell tumor infiltration, and effective memory T‐cell priming. Subsequent research developed a dendritic macromolecule‐encapsulated MnO_2_ nanocarrier for the codelivery of glucose oxidase (GOx) and cGAMP, an agonist of the STING pathway, to enhance tumor chemodynamic/starvation/immunotherapy. By potentiating both local and abscopal immune responses and enabling MR imaging, this approach improved tumor treatment, suppressed the growth of both primary and distant tumors, and significantly increased the overall survival rate of mice. Moreover, the codelivery mediated by dendritic macromolecules not only allowed Mn^2^^+^ to synergize with GOx and cGAMP to enhance STING activation and promote systemic antitumor immune responses but also enabled T1‐weighted tumor MR imaging. This offers great hope for efficient combined tumor treatment and diagnosis and is poised to become a promising nano‐platform for antitumor therapy [440].

AIM2, as a cytoplasmic DNA sensor, can recognize nuclear DNA and mitochondrial DNA released by genomic instability or mitochondrial damage [441]. It activates Caspase‐1 by assembling inflammasomes, enhancing the processing and secretion of IL‐1β and IL‐18, thereby coordinating inflammatory responses and influencing antitumor immunity in the TME [442]. There is a fine mechanism of antagonism and balance between AIM2 and the cGAS–STING pathway. On the one hand, there is crossregulation in their signal output: IFN‐I induced by cGAS–STING pathway activation can upregulate IFN stimulated genes (ISGs), which may have a negative regulatory effect on inflammasome components [443]. On the other hand, under specific cellular environments or stimulus intensities, AIM2 inflammasomes mediate cell death through Caspase‐1, which may cleave key proteins in the cGAS, STING, or IFN signaling pathways, thereby inhibiting cGAS–STING signaling. On the contrary, sustained activation of cGAS–STING may indirectly affect the assembly of AIM2 inflammasomes by inducing cell apoptosis or autophagy. In addition, the types and dynamics of immune cells activated by the two pathways differ. In tumor immunity, the balance between the two may determine the direction of tumor immune response—synergistic activation can promote effective antitumor immunity, while excessive or defective activation of a certain pathway may lead to immune suppression or chronic inflammation [444].

Moreover, in tumor immunity, the DNA sensing pathway mediated by hnRNPA2B1 is a key bridge connecting tumor derived DNA recognition and effective antitumor immune response. When tumor cells release their DNA into the cytoplasm due to genomic instability, therapeutic damage, or viral infection, these abnormal DNA are recognized by the innate immune system [445]. The nuclear RNA binding protein hnRNPA2B1 provides another important and complementary mechanism besides the cGAS–STING pathway. Under normal circumstances, hnRNPA2B1 is involved in RNA processing and transport [446], but when DNA appears in the cytoplasm, it undergoes dimerization and arginine demethylation, changing conformation and being transported to the cytoplasm, directly binding to double stranded DNA [447]. Subsequently, activated hnRNPA2B1 interacts with the adaptive protein STING through its structure, recruits and activates kinase TBK1, and subsequently phosphorylates transcription factor IRF3. After entering the nucleus, IRF3 initiates the expression of IFN‐I and a series of ISGs, triggering strong inflammation and antiviral states [448]. This process is particularly crucial in dendritic cells, as it not only induces IFN production, but also synergistically enhances the expression of MHC‐I antigen presentation‐related genes, thereby optimizing crosspresentation of tumor antigens, effectively activating tumor specific CD8^+^T cells, and forming adaptive immune attacks [449]. Compared with the cGAS–STING pathway, the hnRNPA2B1 pathway does not rely on the second messenger cGAMP, but acts as a direct DNA sensor to activate downstream signals. The two are often activated in different contexts, forming a synergistic network that ensures immune recognition robustness. In tumor immune surveillance and immunotherapy, this pathway has core significance: it can recognize DNA from tumor sources or released during treatment, trigger immune responses, and its activation can produce synergistic effects with radiotherapy, chemotherapy [450], and ICIs [451], enhancing antitumor efficacy. However, tumors may also achieve immune escape by downregulating hnRNPA2B1 expression and other means [452, 453]. Therefore, a deep understanding and targeting of the hnRNPA2B1 pathway provides a promising direction for developing new tumor immunotherapy strategies.

## 4 Mechanisms and Clinical Significance of Nucleotide Metabolism in Regulating Immune System Function and Related Diseases

In recent years, breakthroughs in the field of immune metabolism have revealed that immune cells undergo significant nucleotide metabolic remodeling during activation, differentiation, and execution of effector functions. This metabolic reprogramming is closely related to the strength, duration, and fate of immune responses. Among them, ATP participates in the rapid activation of immune cells as an energy currency, while its degradation product adenosine mediates immune suppression through receptor signaling pathways. The “ATP–adenosine axis” composed of the two plays a pivotal role in the dynamic balance between inflammation initiation and resolution. Current research indicates that nucleotide metabolism disorders are closely associated with pathological processes such as autoimmune diseases (such as systemic lupus erythematosus [SLE]), chronic inflammation, and tumor immune escape. For example, patients with SLE have abnormal ADA activity and eATP accumulation, which directly affect T/B cell dysfunction and autoantibody production. Exploring the dialogue mechanism between nucleotide metabolism and the immune system in depth not only helps to reveal the pathogenesis of immune‐related diseases, but also provides potential targets for the development of new metabolic intervention methods, which has important basic research value and clinical translation prospects.

### 4.1 Core Pathways of Nucleotide Metabolism and the Foundations of Immune Regulation

**FIGURE 4:**
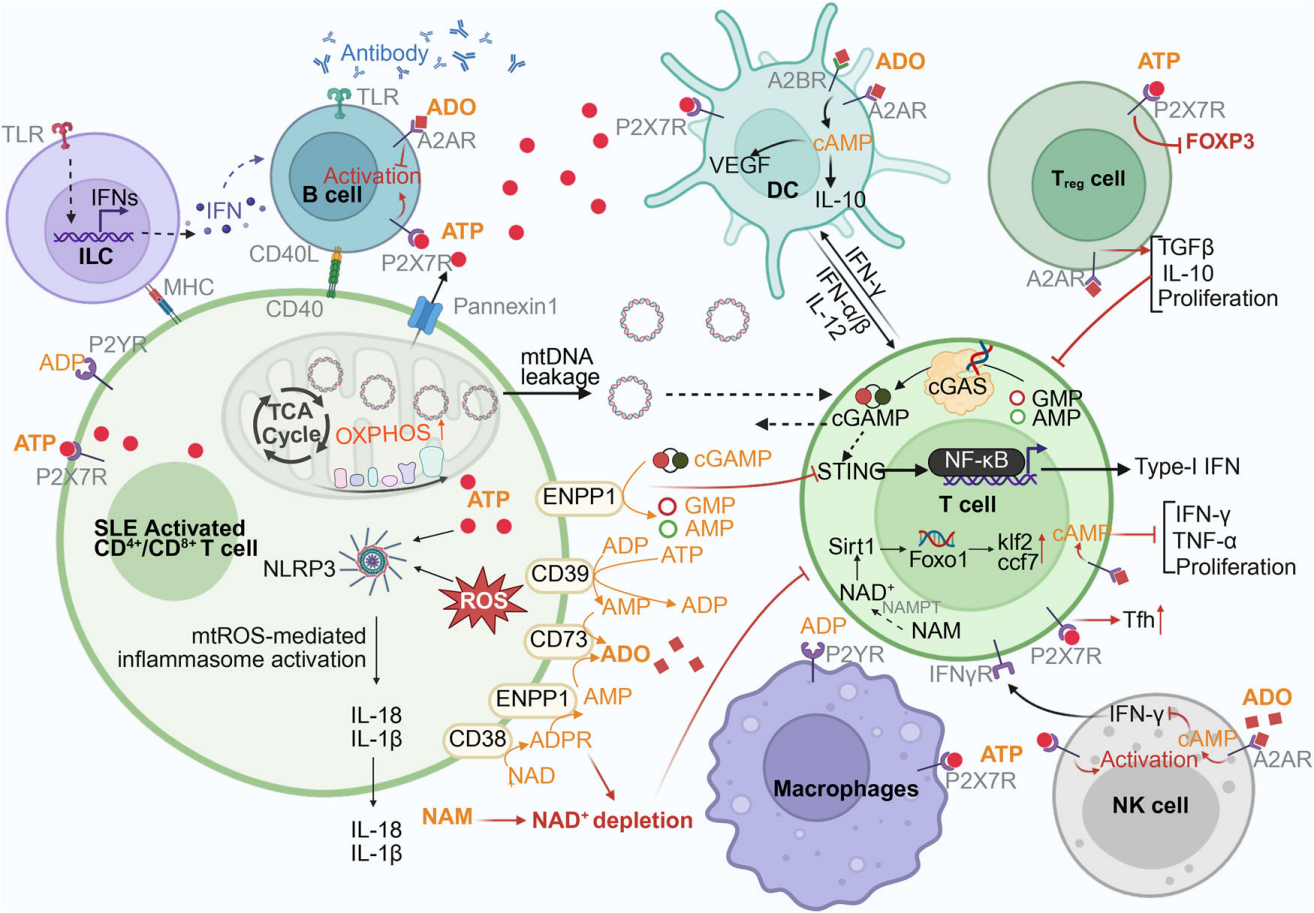
The role of extracellular purinergic signaling in modulating immune cell activation and function. ATP released from damaged cells or as a danger signal is converted to adenosine by ectoenzymes CD39 and CD73, which acts through A2A and A2B receptors to promote immune tolerance and anti‐inflammatory responses. In systemic lupus erythematosus (SLE), impaired CD39/CD73 activity leads to reduced adenosine production and increased ATP levels, activating proinflammatory P2×7R receptors and NLRP3 inflammasomes. This results in heightened production of Type I IFNs, IL‐1β, IL‐18, and autoantibodies, contributing to SLE pathogenesis. Targeting the ATP/adenosine axis through A2AR agonists, CD73 upregulation, or P2×7R inhibition may offer therapeutic benefits by restoring immune homeostasis in SLE and other inflammatory diseases. Created with BioRender.com.

The ICD is intricately linked to the liberation of proinflammatory signals, notably, ATP [454, 455], which triggers the cytosolic sensor cyclic GMP–AMP synthase (cGAS)‐mediated viral defense pathway. cGAS is a cytosolic DNA sensor that recognizes dsDNA. Upon activation, it synthesizes the second messenger 2′,3′‐cGAMP. This cDN, in turn, binds to the stimulator of IFN genes [241], culminating in the production of IFNs and proinflammatory cytokines. This includes the generation of IFN‐I [30] in innate immune cells and the downstream activation of the NF‐ΚB pathway [456], thereby augmenting immunity [457]. In brief, dendritic cells present antigens to T cells and drive T‐cell proliferation more effectively when eATP and cGAS–STING signaling act in concert. ICIs reinforce and prolong this process [458, 459, 460] (Figure 4).

#### 4.1.1 The Proinflammatory Function of ATP and the Immunosuppression of ADO

eATP is typically present at low concentrations in healthy tissues, but its abundance rises sharply during hypoxia, ischemia, tissue injury, or necrotic cell death [461]. This elevation in eATP constitutes a key danger‐associated molecular pattern that sets off inflammatory cascades through P2Y and P2X receptors on immune cells. In myeloid cells, ATP‐mediated activation of P2×7R and P2Y2R stimulates the release of inflammatory mediators, notably, IL‐1β, IL‐18, IL‐6, and IL‐12 [462, 463]. Within the CD8+ effector T‐cell population, signaling through P2×7R and P2×4R facilitates T‐cell clonal expansion and reinforces the persistence of memory T cells. P2×7R on Treg can suppress the expression of FOXP3. In CD4+ T effector cells, activation of P2×7R can promote T follicular helper function. Similarly, P2×7R can facilitate B cell activation.

Adenosine (ADO) is an immunosuppressive metabolite that accumulates from the hydrolysis of eATP by ectonucleotidases [464, 465]. In the TME, ADO promotes the production of IL‐10 and VEGF by myeloid cells through the activation of A2AR and A2BR, inhibiting antigen presentation. In CD8+ effector T cells, ADO activates A2AR, thereby inhibiting IFN‐γ, TNF‐α, and CD8 T cell proliferation. In CD4+ T effector cells, activation of A2AR suppresses IFN‐γ, TNF‐α, and proliferation. ADO can also inhibit other immune cells, such as Treg and B cells.

CD39 and CD73 are two major ectonucleotidases that work together to convert proinflammatory ATP into adenosine, a potent immunosuppressive mediator. The initial step is driven by CD39, which is formally known as ectonucleoside triphosphate diphosphohydrolase 1 or ENTPD1. This enzyme cleaves eATP and ADP to generate AMP [466]. CD73 then takes over and converts this AMP into extracellular adenosine [467]. Beyond these two enzymes, ENPP1 is another ectonucleotidase that can also break down eATP. It is also referred to as CD203a or PC‐1 [468]. The roles of CD38 and ENPP1 in cancer are discussed below.

#### 4.1.2 CD38

CD38 serves as a differentiation marker expressed on immune cells and possesses several enzymatic properties [469, 470]. First, it functions as a cyclase, converting NAD+ into cyclic adenosine diphosphate ribose (cADPR). Second, CD38 also possesses NAD glycohydrolase activity, allowing it to convert NAD+ and cADPR into ADPR [471]. As the primary NAD‐consuming enzyme in mammals, CD38 governs the availability of NAD+ both inside and outside cells. By controlling intracellular NAD+ concentrations, CD38 affects diverse cellular functions ranging from energy metabolism to nonredox reactions. These functions include NAD+‐dependent protein modifications such as mono‐ADP‐ribosylation and poly‐ADP‐ribosylation, which are catalyzed by members of the ADP‐ribosyl transferase and PARP enzyme families, respectively [472, 473, 474]. One 2013 study by Malavasi and coworkers revealed that ENPP1 breaks down CD38‐derived ADPR into AMP, and this product is subsequently converted to adenosine through dephosphorylation by CD73 [475, 476].

The expression of CD38 on both immune and malignant cells is subject to upregulation by multiple TME‐derived factors, such as TNF‐α, IFN‐γ, retinoic acid, and IFN‐β, reflecting a pathological adaptation to inflammatory signals [129, 477]. CD38 expression also increases in colorectal cancer patients postchemotherapy [478] and in melanoma patients undergoing anti‐PD‐1 treatment [129]. In addition, both human and mouse breast cancer cells exhibit dose‐dependent upregulation of CD38 following ionizing radiation [479].

Using murine models of lung cancer, the group led by Chen demonstrated that CD38‐dependent adenosine generation underlies resistance to PD‐1/PD‐L1 checkpoint blockade [129]. According to their model, retinoic acid upregulates CD38 on neoplastic cells through signals emanating from PD‐1/PD‐L1. Eliminating IFN‐β and CD38 either genetically or pharmacologically augments the antitumor efficacy of PD‐L1 inhibition, bolstering CD8 T cell‐driven immune surveillance. The investigators further established a link between CD38 expression and adenosine output, with the latter dropping after antibody‐mediated CD38 neutralization [480, 481, 482]. Extracellular NAD+ binds to P2×7 receptors on T cells and can elicit a form of cell death known as NICD. In NSCLC models, tumor cells expressing ART1 trigger NICD in CD8 T cells bearing P2×7R, and CD38 ablation aggravates this process, implying that CD38 on P2×7R‐positive CD8 T cells guards against NICD. In the setting of solid tumors, particularly after irradiation when extracellular NAD+ accumulates, the NADase function of CD38 protects T lymphocytes from NICD [483, 484]. Overall, these results suggest that for patients harboring ART1‐positive malignancies, concomitant use of CD38 and ICIs may lead to unintended toxicity through enhanced NICD in CD8 T cells infiltrating the TME.

#### 4.1.3 ENPP1

ENPP1 hydrolyzes ATP and ADPR, or directly hydrolyzes NAD+ into AMP, which is subsequently converted to adenosine by CD73 [476]. Studies have demonstrated that ENPP1, as an extracellular membrane‐bound enzyme, possesses the ability to hydrolyze extracellular cGAMP [55]. The physiological concentration of cGAMP in the extracellular matrix is regulated by the cGAMP hydrolase activity of ENPP1. By diminishing cGAMP's capacity to propagate immune signaling, this process generates AMP as a precursor for immune‐suppressive adenosine, thereby positioning ENPP1 as a potential immunotherapeutic target for intervening in cGAS–STING signaling pathways. Nonmembrane‐permeable inhibitors of ENPP1 can substantially elevate plasma cGAMP levels, bolstering immune responses and mitigating tumor burden, with related drugs currently undergoing clinical trials [485].

High expression levels of ENPP1 in breast cancer have been correlated with unfavorable prognosis [486] and increased incidence of bone metastases [487]. When breast tumors are irradiated, they release cGAMP into the extracellular milieu, and this release drives STING‐mediated immunogenicity, as recently reported by Carozza and coworkers. In mouse breast cancer models, both the knockout of the ENPP1 gene and the administration of ENPP1 inhibitors have been found to hinder tumor progression [488]. Intriguingly, radiation augments ENPP1 surface expression on 4T1 cells, suggesting that radiation‐induced activation of cGAS is accompanied by a regulatory mechanism aimed at dampening immune activation [479].

In 4T1 and other tumor types, gene knockout resulting in the loss of intratumoral ENPP1 has been demonstrated to decrease adenosine production in vitro and is linked to an increase in tumor‐infiltrating activated T cells and enhanced responses to ICIs in vivo [55]. In STING‐deficient mice, the antitumor or protumor effects of ENPP1 knockout or overexpression are attenuated, indicating that ENPP1 primarily promotes tumor progression by impeding STING activation by cGAMP in host cells. Furthermore, ENPP1 expression is notably higher in distal metastatic cancer cells compared with primary tumors, whereas an opposing expression pattern is observed for CD39, suggesting that adenosine derived from cGAMP, rather than ATP, dominates in some advanced and metastatic cancers.

A number of investigational agents directed against CD39, CD73, ENPP1, and the A2A/A2B adenosine receptors are currently being developed, several of which have advanced into clinical trials [489, 490]. Multiple inhibitors of the ectonucleotidase CD73 are being evaluated in clinical trials, either as monotherapy or in combination with other therapies such as A2A and/or A2B receptor antagonists and ICIs. These agents have exhibited manageable toxicity in early studies, albeit with limited activity as monotherapy [491]. In a randomized study of patients with Stage III non‐small cell lung cancer undergoing chemoradiotherapy, the addition of the anti‐CD73 antibody oleclumab improved the overall response rate [54] and progression‐free survival compared with anti‐PD‐L1 monotherapy [160, 492, 493]. Inhibiting CD73 blocks the terminal step of adenosine production, offering perhaps the most effective strategy for lowering adenosine within the TME. In contrast, therapeutic targeting of CD39 or ENPP1 is intended to promote the buildup of immunostimulatory ATP and cGAMP, respectively. For malignancies characterized by high adenosine activity, including renal cell carcinoma and colorectal cancer, A2A receptor blockade may represent the optimal therapeutic approach [319].

In summary, combined inhibition therapies should be customized to the specific characteristics of the TME [494]. In radiotherapy, CD39 blockade would raise eATP that stimulates immunity. Yet this gain may be undermined by adenosine produced through CD39‐independent mechanisms involving NAD+ and cGAMP. CD38 serves as an alternative route for adenosine generation. Nevertheless, clinical efforts to block CD38‐dependent adenosine production have thus far been unsuccessful [480], highlighting the need for further research on CD38's role in immune regulation [484]. Additionally, the radiation‐induced increase in adenosine within the TME suggests that targeting both A2A and the lower‐affinity A2B receptors may be beneficial.

### 4.2 Abnormal Nucleotide Metabolism and Autoimmune and Inflammatory Diseases

#### 4.2.1 Nucleotide Metabolism Disorder in SLE

SLE is a classic autoimmune disease in which poorly controlled immune responses give rise to systemic inflammation and multiorgan injury. Its clinical course is notoriously unpredictable, marked by alternating periods of inactive disease and sudden flare‐ups that often progress to permanent organ dysfunction. Patients with lupus experience disproportionately high rates of both illness and mortality, with lupus nephritis, cardiovascular pathology, and infections being the major contributing factors. The disease exhibits a profound sexual dimorphism, affecting females nine times more frequently than males, and disproportionately afflicts racial and ethnic minorities [495, 496]. While the current therapeutic armamentarium is extensive, the burden of broad immunosuppression and corticosteroid‐associated cardiometabolic sequelae remains significant. This therapeutic dilemma highlights an urgent need for targeted interventions capable of modulating immune responses without establishing persistent systemic immunosuppression. Emerging translational evidence posits that such precision may be achieved by targeting the metabolic substrate of inflammation: the fundamental rewiring of nucleotide metabolism within immune cells [497].

Mitochondrial bioenergetics serve as a critical checkpoint coupling energy metabolism to nucleotide biosynthesis. SLE T cells display a distinct metabolic phenotype defined by mitochondrial hyperpolarization and elevated reactive oxygen species, a state primarily attributable to Complex I dysfunction [498]. Crucially, this bioenergetic stress governs de novo pyrimidine synthesis via DHODH, an enzyme physically anchored to the inner mitochondrial membrane and tethered to the electron transport chain. By relying on the ubiquinone pool as an obligate electron acceptor, DHODH renders pyrimidine production dependent on the altered redox landscape of lupus T cells, acting as a metabolic rheostat that links mitochondrial respiration to lymphocyte proliferation [499]. This crosstalk is further cemented by the upregulation of the mitochondrial folate enzyme MTHFD2 in proliferating immune cells; by utilizing the cofactor FAD to drive the formate pool requisite for purine synthesis, MTHFD2 enforces a robust proinflammatory metabolic state [500].

Parallel to mitochondrial reprogramming, cytosolic metabolism undergoes a fundamental shift to meet the heightened biosynthetic demands of autoimmunity. Under the drive of constitutive mTORC1 activation, glucose flux in lupus lymphocytes is forcibly redirected from glycolysis into the PPP. This metabolic diversion fulfills a dual requirement: generating NADPH to buffer oxidative stress and providing R5P as the essential backbone for nucleotide synthesis. As highlighted in recent reviews, dysregulated flux through this pathway actively supports the autoimmune response by facilitating the rapid clonal expansion of autoreactive cells [501]. Downstream of this axis, the reliance on expanded guanine nucleotide pools identifies IMPDH as a key metabolic bottleneck. The clinical success of mycophenolate mofetil, a specific IMPDH inhibitor, validates the strategy of targeting the de novo nucleotide synthetic machinery to abrogate lymphocyte hyperactivity, sparing patients from the generalized toxicity associated with broad‐spectrum immunosuppressants [502].

The culmination of these intracellular metabolic perturbations manifests as a self‐amplifying inflammatory loop between the intracellular and extracellular compartments. In the SLE microenvironment, necrotic cell death and NETosis mediate the massive release of ATP, which acts as a pervasive damage‐associated molecular pattern. Upon binding to the P2×7 purinergic receptor, eATP triggers NLRP3 inflammasome assembly and the proteolytic maturation of IL‐1β and IL‐18 [503]. In SLE, the physiological brake normally provided by the ectonucleotidases CD39 and CD73 is compromised; this enzymatic impairment leads to persistent ATP stimulation and a deficiency in immunosuppressive adenosine, thereby promoting Th17 differentiation [504]. This extracellular dysregulation is further exacerbated by the leakage of mitochondrial components into the cytosol and beyond. Bioenergetic stress within the mitochondrion drives the release of oxidized mitochondrial DNA, which, by evading autophagic clearance, engages the cGAS–STING and TLR9 nucleic acid‐sensing pathways. This persistent activation of innate sensing mechanisms sustains the IFN‐I signature that characterizes the disease [505]. Thus, the breakdown of immune tolerance is orchestrated by a multidimensional pathology wherein nucleotide metabolism—spanning mitochondrial respiration, cytosolic biosynthesis, and extracellular signaling—integrates to drive disease progression.

#### 4.2.2 Abnormal Nucleotide Metabolism in Other Inflammatory Diseases

The pathological process of rheumatoid arthritis (RA) is closely related to the abnormal accumulation of purine metabolite uric acid [506]. The concentration of uric acid in synovial fluid of RA patients was significantly higher than that in serum. Uric acid crystallization induces the secretion of proinflammatory cytokines TNF‐α and IL‐6 by activating TLR4 receptor on the surface of macrophages, and promotes the proliferation and formation of synovial fibroblasts [507]. Clinical studies have shown that the joint damage of hyperuricemia RA patients is more serious than that of normal uric acid group [508], and the response rate to MTX treatment is reduced. Uric acid can also indirectly increase the level of adenosine in synovial tissue by inhibiting the activity of ADA, which promotes osteoclast differentiation and aggravates bone erosion through A2A receptor. IBD includes Crohn's disease [2] and ulcerative colitis (UC). The abnormality of nucleotide metabolism is mainly manifested in the disorder of adenosine signaling pathway. The expression of CD73 in the intestinal mucosa of CD patients was significantly upregulated, which was higher than that of normal mucosa, resulting in the continuous hydrolysis of AMP in the intestinal cavity to adenosine, and the local adenosine concentration increased [509]. High concentration adenosine activates ERK1/2 pathway of intestinal epithelial cells through A2B receptor, promotes phosphorylation of tight junction protein occludin, increases intestinal permeability, causes antigen translocation in intestinal cavity, and intensifies mucosal immune response [510]. In UC patients, the function of P2×7 receptor is defective, and the expression of P2×7 receptor mRNA in colonic mucosa is reduced by 40%, which leads to the activation of ATP induced inflammatory corpuscles, and the secretion of IL‐1β is reduced, which cannot effectively remove intestinal pathogens and form a chronic inflammatory state [511].

Nucleotide metabolic abnormalities in different inflammatory diseases have similarities and specificities. The common performance is the vicious cycle of “inflammation metabolite accumulation inflammation amplification”: inflammation activation leads to cell damage and releases a large amount of ATP. Abnormal expression of metabolic enzymes leads to imbalance of metabolite transformation. Metabolites further aggravate inflammation through receptor signaling. Specificity is reflected in the dominant type of metabolic abnormalities: RA is dominated by accumulation of purine decomposition products, IBD is characterized by disorder of adenosine signaling pathway, and psoriasis is characterized by over activation of atp–p2×7–nlrp3 axis. This difference in metabolic characteristics provides a theoretical basis for the precise targeted treatment of the disease. For example, uricase inhibitors can reduce the level of synovial uric acid in RA patients, and CD73 inhibitors can reduce intestinal inflammatory injury in CD model [512].

In summary, current research has clarified that nucleotide metabolism regulates immune system function through a dual mechanism of metabolic reprogramming and signaling molecules [513]. During the activation of immune cells, T/B lymphocytes undergo a metabolic shift from oxidative phosphorylation to glycolysis, accompanied by enhanced purine/pyrimidine synthesis. Key enzymes such as PRPP synthetase and HGPRT influence cell proliferation and differentiation by regulating nucleotide supply. At the metabolite level, eATP acts as a DAMP molecule to activate the NLRP3 inflammasome, while adenosine mediates immune suppression through A2A/A2B receptors. The metabolic axis formed by these two plays a dynamic balancing role in the initiation and resolution of inflammation. Disease‐associated studies have revealed metabolic disorders in SLE patients, such as reduced ADA activity and abnormal ATP release, leading to immune cell dysfunction and the production of autoantibodies. RA and IBD also exhibit characteristic changes in purine/pyrimidine metabolic pathways. However, there are still critical scientific questions that need to be addressed in the field: the complexity of metabolic networks limits the effectiveness of single‐target interventions; for example, CD73 inhibitors may simultaneously affect adenosine homeostasis in the TME and normal tissues. The metabolic heterogeneity of immune cell subpopulations has not been fully elucidated, and the differences in nucleotide metabolism dependency among different T cell subpopulations require further exploration. In clinical translation, the lack of specificity of metabolic markers and off‐target effects of drugs restrict the precision of targeted therapies. In the future, the application of metabolomics technology will further advance immune metabolism research [514]. Single‐cell metabolomics can reveal the metabolic heterogeneity of immune cell subpopulations, while spatial metabolomics can visually present the distribution characteristics of nucleotide metabolites in the inflammatory microenvironment. Combined with multiomics integrative analysis, new metabolic regulatory nodes are expected to be discovered. Personalized metabolic targeted therapy is an important direction for clinical translation, achieving stratified treatment by detecting patients’ purine/pyrimidine metabolic profiles, such as serum adenosine levels and PRPS activity. Additionally, combination therapy strategies based on metabolic characteristics, such as the concurrent use of CD73 inhibitors and A2A receptor antagonists, can synergistically block the adenosine immune suppression pathway, reducing the dosage and toxicity of single agents. Basic research must overcome key pathways for clinical translation: simultaneously developing highly specific metabolic markers for disease classification and efficacy monitoring; establishing organoid models to simulate disease microenvironments, optimizing drug screening processes [515], and improving targeted delivery efficiency through technologies such as antibody–drug conjugates and nanoparticle carriers to minimize metabolic interference with normal tissues [516]. With innovations in technological methods and the deepening of translational medicine, nucleotide metabolism is expected to become a new breakthrough in the precise treatment of immune‐related diseases.

## 5 Gout and Calculus—Abnormal Purine Catabolism

As previously mentioned, purine metabolism is a core aspect of nucleotide metabolism in the human body. An imbalance in the steady state of its catabolic pathways can trigger a series of metabolic disorder syndromes centered around hyperuricemia [517]. Purines are ultimately degraded to uric acid through a series of enzymatic reactions in the body. When the rate of uric acid production exceeds the capacity for excretion, the plasma uric acid concentration rises abnormally, leading to hyperuricemia [518]. This metabolic abnormality is not only the pathological basis of gouty arthritis but is also closely associated with urate nephrolithiasis, chronic kidney disease (CDK), and various other metabolic syndromes. In recent years, with the advancement of molecular biology techniques, the gene regulatory mechanisms of key enzymes and transport proteins in purine metabolism have gradually become clearer, providing new perspectives for the precise diagnosis and targeted treatment of diseases. However, clinical practice still faces challenges such as insufficient early intervention, drug resistance, and management of complications. A deeper understanding of the molecular mechanisms underlying abnormal purine catabolism and exploring its interactions with multisystem diseases is of significant clinical importance for improving the prevention and treatment of related diseases such as gout and stones.

### 5.1 Metabolic Basis of Hyperuricemia

Uric acid is the final product of the purine metabolic pathway, with a normal daily production of about 700 mg in adults, two‐thirds of which is excreted by the kidneys. As a direct marker of hyperuricemia [519], blood uric acid levels reflect the overall balance of the purine metabolic network. Abnormally elevated concentrations are a result of metabolic disorders, leading to diseases such as gout and uric acid stones [520]. It is noteworthy that uric acid has a dual biological effect. At physiological concentrations, it can exert antioxidant effects by scavenging reactive oxygen species and protecting vascular endothelial cells. However, excessive accumulation can trigger inflammatory responses and crystal deposition, exhibiting a dual‐edge sword characteristic in physiology and pathology [521].

**FIGURE 5:**
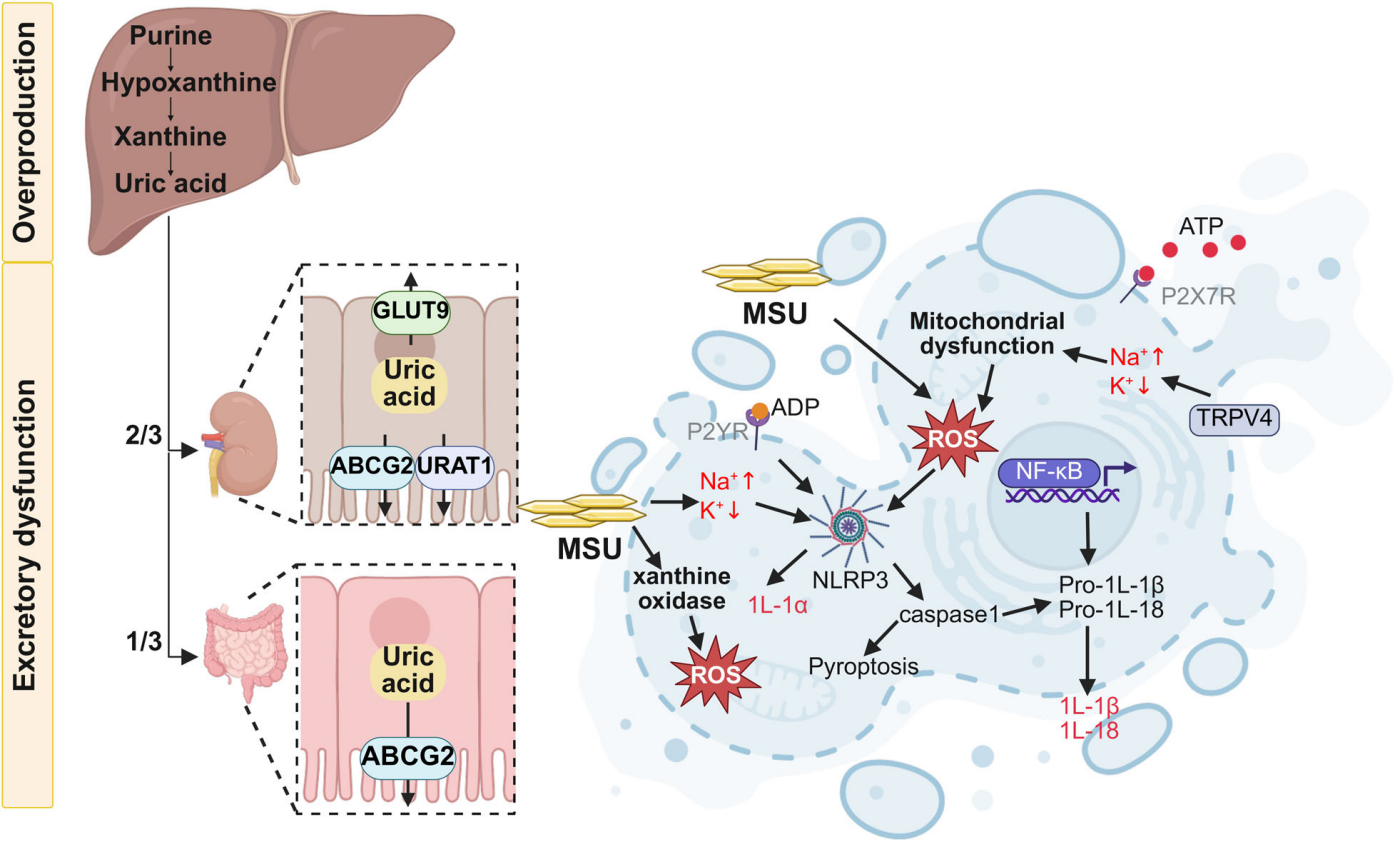
The pathophysiology of gout. This involves three key processes: overproduction of uric acid, impaired excretion, and abnormal secretion. Overproduction occurs through the metabolic conversion of purines into hypoxanthine, xanthine, and ultimately uric acid. Impaired excretion is characterized by reduced transport of uric acid into the renal tubules via the ABCG2/URAT1 pathway, coupled with increased reabsorption through the GLUT9 channel, leading to hyperuricemia. Monosodium urate (MSU) crystals trigger inflammation by inducing mitochondrial dysfunction, which results in the production of reactive oxygen species (ROS). This activation subsequently triggers the NLRP3 inflammasome, leading to the release of proinflammatory cytokines such as IL‐1β and IL‐18. Extracellular ATP further amplifies the inflammatory response by activating P2×7 and TRPV4 receptors, ultimately leading to the characteristic symptoms of gout. Created with BioRender.com.

Increased purine metabolism leads to excessive uric acid production. The homeostatic regulation of the purine metabolic network relies on the precise regulation of key enzymes. Abnormal function or genetic mutations significantly enhance the rate of purine synthesis, resulting in hyperuricemia due to excessive uric acid production. PRPS catalyzes the formation of PRPP from ATP and 5‐phosphoribose, and it is subject to allosteric inhibition by purine nucleotide products. Gain‐of‐function mutations in the PRPS gene (such as the R190Q variant of the PRPS1 gene) can relieve the inhibitory effect of the products, leading to a surge in PRPP synthesis, driving the massive production of purine nucleotides like IMP and AMP, ultimately increasing uric acid output by three to five times. Patients with this condition often present with early‐onset and refractory hyperuricemia. Deficiency of HGPRT blocks the reutilization of hypoxanthine and guanine, leading to substrate accumulation and entry into the catabolic pathway. Meanwhile, PRPP accumulates due to the inability of salvage pathways to consume it, activating de novo synthesis pathways in a “double hit” effect. For example, LNS is caused by a complete deficiency of HGPRT, presenting with severe hyperuricemia and accompanying neurological symptoms [522]; partial deficiency leads to Kelley‐Seegmiller syndrome, characterized mainly by isolated hyperuricemia and urinary stones [523]. XO plays a decisive role in the two‐step oxidation reaction from hypoxanthine to xanthine to uric acid, with its activity regulated by cofactors such as molybdenum and iron‐sulfur clusters. Genetic polymorphisms in the XO gene (such as rs2231142) can affect enzyme activity [524]. Increased dietary purine intake or accelerated nucleic acid breakdown due to cellular damage can enhance XO catalytic activity through substrate induction effects, becoming an important mechanism for secondary hyperuricemia (Figure 5).

Abnormal renal uric acid excretion leads to excessive uric acid levels. The kidneys are the primary organs for uric acid excretion, involving four processes: glomerular filtration, proximal tubular reabsorption, secretion, and postsecretion reabsorption, all coordinated by various transport proteins [525]. Urate anion transporter 1 (URAT1 [526]) is a key reabsorption protein that mediates the active reabsorption of urate through exchange with chloride ions. Gene‐activating variants (such as p.W258X) or functional enhancements can lead to increased urate reabsorption, commonly causing familial hyperuricemia. Clinical studies show that hyperactive URAT1 can reduce uric acid excretion fractions by over 30%, making it one of the main molecular mechanisms of primary hyperuricemia [527]. Glucose transporter 9 (GLUT9) has two splice isoforms, with GLUT9a located on the basolateral membrane participating in urate excretion, while GLUT9b is expressed on the brush border membrane to promote urate reabsorption [528]. Loss‐of‐function mutations can lead to intracellular urate accumulation, causing renal urate excretion disorders, and this gene variant is significantly associated with hyperuricemia in women [529]. ATP‐binding cassette transporter G2 (ABCG2) is a urate efflux pump expressed in the proximal tubules of the kidneys and the intestines. Functional loss variants such as Q141K can simultaneously reduce renal and intestinal urate excretion, leading to a 1.5–2‐fold increase in blood uric acid levels, representing an important genetic risk factor for the development of gout [530].

### 5.2 Pathophysiological Process of Hyperuricemia Mediating Gout

#### 5.2.1 Formation of Monosodium Urate Crystals and Mechanism of Joint Cavity Deposition

The formation of monosodium urate (MSU) crystals is the core pathological event in the occurrence of gout, strictly dependent on the supersaturation of blood uric acid and the local microenvironment. When plasma uric acid concentration exceeds its physical solubility, urate precipitates in the form of MSU, forming needle‐like or rod‐like crystals [531]. The temperature gradient is a key influencing factor for joint cavity deposition; peripheral joints such as the first metatarsophalangeal joint and the ankle joint have lower temperatures, resulting in a 15–20% decrease in uric acid solubility compared with core body temperature, making them common sites for crystal deposition. Conditions such as inflammation that lead to a decrease in the pH of synovial fluid further reduce urate solubility, promoting crystal nucleation [532]. Additionally, the loss of glycosaminoglycans on the surface of articular cartilage and micro‐injuries to the synovial tissue provide attachment sites for MSU, while a decrease in hyaluronic acid concentration in the joint fluid weakens its inhibitory effect on crystal formation. MSU crystals exhibit specific morphological characteristics, appearing as elongated needle‐like structures under optical microscopy, with negative birefringence and bright yellow refraction under polarized light microscopy. Scanning electron microscopy reveals characteristic step‐like structures on the crystal surface, which may participate in the recognition process by immune cells. Joint cavity deposition has certain tissue specificity, occurring not only in the synovial lining but also in the matrix of articular cartilage, subchondral bone, tendons, and their attachment points, forming tophi [533]. Clinically, crystal detection can be performed through joint fluid aspiration microscopy, dual‐energy CT, and ultrasound (“double track sign” or “snowstorm sign”), with polarized light microscopy of joint fluid remaining the gold standard for diagnosis [534].

#### 5.2.2 Immune Cell Recognition and NLRP3 Inflammasome Activation Pathway

Once MSU crystals deposit in the joint cavity, they can be recognized by innate immune cells, triggering a vigorous inflammatory response [535]. Macrophages, as resident immune cells in the joint, sense crystal signals through surface pattern recognition receptors, with scavenger receptor A and Fcγ receptors (FcγR) mediating the initial adhesion and phagocytosis of the crystals. After being engulfed by macrophages, the crystals accumulate in phagolysosomes, where their physical stimulation leads to decreased lysosomal membrane stability, ultimately resulting in lysosomal rupture. Lysosomal enzymes such as cathepsin B are released into the cytoplasm, along with ROS, forming upstream triggering signals that activate the NLRP3 inflammasome [536]. The activation of the NLRP3 inflammasome involves two stages: priming and activation. The priming stage is induced by proinflammatory signals, such as TNF‐α and IL‐1α, through the NF‐κB pathway, leading to the expression of NLRP3 and pro‐IL‐1β genes [537]. The activation stage is mediated by MSU crystals, which induce conformational changes in the NLRP3 protein, allowing it to interact with the adaptor protein ASC through PYD–PYD domains [538]. ASC then recruits pro‐Caspase‐1 through CARD–CARD domains, forming a multiprotein complex. The assembly of this complex promotes the autocleavage of pro‐Caspase‐1, generating active Caspase‐1 p20/p10 heterodimers. Studies have found that ATP released from lysosomal rupture can also activate the P2×7 receptor on the cell membrane [539], leading to potassium ion efflux, further amplifying the activation signal of the NLRP3 inflammasome, creating a cascade amplification loop of “crystal phagocytosis—lysosomal rupture—ionic imbalance.”

#### 5.2.3 Release of Inflammatory Mediators and Close Association With Acute Gout Attacks

Activated Caspase‐1 specifically cleaves inactive pro‐IL‐1β in the cytoplasm into mature IL‐1β, which is released extracellularly through gasdermin D‐mediated pyroptosis [540]. IL‐1β, as the initiating factor of acute gout attacks, binds to IL‐1 receptors on the surface of synovial cells and fibroblasts, activating MAPK and NF‐κB signaling pathways, inducing the expression of chemokines CXCL8/IL‐8 and adhesion molecules ICAM‐1. These mediators recruit neutrophils through the bloodstream, leading to their massive accumulation in the joint cavity, forming a vicious cycle of “neutrophil infiltration—release of inflammatory mediators—further cell recruitment” [541]. After phagocytosing MSU crystals, neutrophils release inflammatory mediators such as myeloperoxidase and elastase through degranulation, exacerbating synovial tissue damage. Meanwhile, the extracellular traps released by neutrophils can encapsulate and clear crystals, but their degradation products (such as DNA and histones) have proinflammatory activity, further prolonging the inflammatory response [542]. IL‐6, as a downstream inflammatory factor, participates in systemic inflammatory responses by inducing the liver to synthesize acute phase proteins (such as C‐reactive protein), with serum levels positively correlated with the severity of acute gout attacks [543]. TNF‐α enhances vascular permeability, promoting the exudation of inflammatory cells and worsening joint swelling. This inflammatory cascade can peak within hours, clinically presenting as sudden severe joint pain, redness, swelling, and limited movement. In the later stages of inflammation, monocytes replace neutrophil infiltration [544], phagocytosing and clearing crystals and necrotic tissue, gradually entering the intercritical period, but residual MSU crystals become foci for future recurrences.

### 5.3 Therapeutic Drug Targets and Applications for Diseases Related to Abnormal Purine Metabolism

#### 5.3.1 Mechanism of Action and Clinical Value of XO Inhibitors

XO inhibitors selectively inhibit the activity of XO, blocking the conversion of hypoxanthine to xanthine and xanthine to uric acid, thereby reducing uric acid production at the source. They are first‐line medications for the treatment of hyperuricemia and gout. Allopurinol, as the first marketed XO inhibitor, exerts its effect by competitively inhibiting the molybdenum‐pterin active site of XO [545]. Its active metabolite, oxypurinol, has a half‐life of 18–30 h, allowing for long‐lasting inhibition [546]. Pharmacokinetic studies show that allopurinol is rapidly absorbed in the gastrointestinal tract after oral administration (with a bioavailability of about 80%), reaching peak blood concentration in 2–6 h, and is primarily excreted by the kidneys. Clinical data indicate that an initial dose of 100 mg/day of allopurinol can lower blood uric acid levels by approximately 20%‐30%, while a maximum dose of 800 mg/day can achieve a reduction of over 50% [547].

Febuxostat, as a novel nonpurine XO inhibitor, tightly binds to the molybdenum atom of XO through the imidazole ring in its molecular structure, demonstrating higher specificity and potency in inhibition [548, 549]. Its oral absorption is not affected by food, with a peak time of 1–2 h and a half‐life of 5–8 h, primarily metabolized by the liver (about 49% excreted in feces), and does not require dose adjustment in patients with renal impairment. Compared with allopurinol, febuxostat lowers uric acid levels more rapidly, with a dosage of 40 mg/day capable of reducing blood uric acid by 40–50%, and its inhibitory effect on XO is not influenced by purine analogs [550].

The clinical advantages of allopurinol include its low cost and extensive experience with long‐term use, making it suitable for most patients with excessive uric acid production. However, it is important to be cautious of severe skin allergic reactions (occurrence rate of 0.1–0.4%) [551], and the risk is significantly increased in HLA‐B*58:01 gene positive populations [552]. Genetic screening is recommended before medication. Febuxostat, due to its dual renal and hepatic excretion characteristics, is more suitable for patients with renal impairment and has a lower incidence of allergic reactions, but the FDA warns that it may increase the risk of cardiovascular mortality, necessitating an assessment of patients’ underlying cardiovascular diseases during clinical use [553].

#### 5.3.2 Molecular Targets and Applications of Uric Acid Excretion Promoting Drugs

Uric acid excretion promoting drugs can increase uric acid excretion fraction and reduce blood uric acid level by selectively inhibiting urate reabsorption transporter in renal proximal tubule, which is mainly applicable to hyperuricemia with reduced uric acid excretion [554]. Benzbromarone, as a powerful URAT1 inhibitor, significantly reduces the reabsorption of uric acid in proximal tubules by blocking the urate chloride exchange process by competitively binding to the substrate binding site of URAT1 with urate [555]. Clinical studies showed that the daily use of 50 mg benzbromarone could increase the uric acid excretion fraction from the baseline 10% to 20–25% and reduce the blood uric acid level by 40–50% [551]. Lesinurad is a new selective URAT1 inhibitor, which can simultaneously inhibit organic anion transporter 4 and double block the pathway of uric acid reabsorption. Monotherapy can reduce blood uric acid by 20–30%, showing a synergistic effect with XO inhibitor [556].

#### 5.3.3 Progress in the Application of Uricase Drugs in Refractory Cases of Purine Metabolism Abnormalities

Uricase drugs mimic the physiological function of uricase, directly catalyzing the conversion of uric acid into allantoin, which has a solubility 10 times greater than uric acid, and is excreted by the kidneys, providing a new treatment strategy for refractory gout that is ineffective with traditional therapies [557]. Pegloticase is currently the only approved uricase drug, modified with PEG to extend its half‐life to 6–10 days while reducing immunogenicity [558]. Its mechanism of action does not rely on renal excretion but directly breaks down circulating uric acid molecules: 1 mg of pegloticase can degrade approximately 150 mg of uric acid. The recommended dose of 8 mg is administered intravenously every 2 weeks, which can lower blood uric acid levels to below 100 µmol/L within 24 h, and reduce the volume of tophi by over 50% within 6 months. Clinical evidence shows that pegloticase is suitable for patients with refractory gout who have been treated adequately with XO inhibitors for more than 3 months, with blood uric acid still over 480 µmol/L, and who have at least one palpable tophus. The clinical trial demonstrated that pegloticase achieved a target rate of blood uric acid below 300 µmol/L of 42%, significantly higher than the placebo group (5%), and the frequency of acute gout attacks decreased by 70% after 6 months of treatment [559]. For refractory patients with CDK (Stages III‐IV), pegloticase can significantly improve renal function indicators without dose adjustment, with an average increase in eGFR of 5–8 mL/min, possibly related to reduced uric acid load [560]. In terms of administration, pegloticase requires intravenous infusion over more than 2 h, and prophylactic antihistamines and corticosteroids should be used before the first dose to reduce the risk of allergic reactions. Immunogenicity limits the use of pegloticase, with about 15–20% of patients developing neutralizing antibodies, leading to accelerated drug clearance and decreased efficacy, with some patients experiencing infusion reactions such as fever, rash, and difficulty breathing, and in severe cases, anaphylactic shock [561]. Uricase may crossreact with uric acid transport proteins on red blood cell membranes, so long‐term use also requires vigilance for hemolytic anemia and infusion‐related reactions [562]. Currently, many new uricase formulations have been developed, such as PEGylated recombinant uricase and uricase‐albumin fusion proteins, aiming to optimize PEG modification processes and molecular structures to extend half‐life and reduce immunogenicity, providing better options for refractory gout patients [563].

In summary, abnormalities in purine metabolism connect genetic factors, metabolic disorders, and inflammatory responses in hyperuricemia, gout, and uric acid stones. The enzyme regulatory axis formed by PRPS–HGPRT–XO determines the rate of uric acid production, while the transport protein system composed of URAT1–GLUT9–ABCG2 dominates the balance of uric acid excretion. The imbalance of these two functions jointly drives the occurrence of hyperuricemia. The pathological process of urate crystal activation of the NLRP3 inflammasome revealing a new mechanism of metabolite‐driven inflammation. Current research still lacks clarity in areas such as intracellular signaling regulation of transport proteins, interactions between gut microbiota and uric acid metabolism [558], and epigenetic regulation of inflammasome activation. Future precision medicine strategies will further focus on gene testing‐based stratified treatment, targeted intervention in inflammatory pathways, and the development of new uric acid metabolic regulators, such as prioritizing uricosuric drugs for ABCG2 variants, NLRP3 inhibitors, IL‐1β monoclonal antibodies, uric acid transport protein modulators, and gut uric acid‐decomposing bacterial preparations [564].

## 6 Nucleotide Metabolism and Neurological Diseases

The nervous system has a special dependence on nucleotide metabolism, closely related to its structural complexity and high energy consumption characteristics. Neurons, as terminally differentiated cells, resulting in a continuous and stable demand for nucleotides for DNA repair and RNA renewal. During synaptic transmission, the release of neurotransmitters, operation of ion pumps, and signal transduction all rely on ATP hydrolysis for energy supply, while purine substances act as neurotransmitters or modulators, participating in key physiological processes such as synaptic plasticity regulation, neuroinflammatory responses, and neural regeneration by activating specific receptors [565]. Additionally, the presence of the blood–brain barrier limits the direct utilization of peripheral nucleotides, making brain nucleotide metabolism more reliant on the synergistic action of endogenous synthesis and salvage pathways [566]. This also makes the nervous system more sensitive to metabolic enzyme deficiencies or substrate supply imbalances. Recent studies have shown that abnormalities in nucleotide metabolism are closely related to the occurrence and development of various neurological diseases [567]. In hereditary metabolic disorders, enzyme deficiencies lead to the accumulation of purine/pyrimidine metabolic intermediates or the lack of key products, which can directly cause neuronal damage or induce progressive neurodegeneration by affecting mitochondrial genome replication. Furthermore, during cerebral ischemia/stroke, the massive release of eATP activates P2×7 receptors, triggering calcium overload and inflammatory cascades, becoming an important mechanism of secondary brain injury [568]. Research on the association between nucleotide metabolism and neurological diseases can be traced back to the 1950s, when the causal relationship between HGPRT deficiency and LNS was first established, pioneering the study of metabolic neurological diseases. With the development of molecular biology techniques, the discovery of purinergic signaling systems, and the cloning of receptor subtypes, a new functional dimension of nucleotides as neuromodulators has been revealed. In recent years, the application of metabolomics, gene editing, and brain imaging technologies has further promoted in‐depth analysis of the mechanisms by which nucleotide metabolism disorders lead to neuronal damage, providing new perspectives for the discovery of disease diagnostic markers and therapeutic targets [569]. Although current research has confirmed the central role of nucleotide metabolism in neurophysiology and pathology, the specific molecular mechanisms of most diseases remain to be elucidated, and the development of specific drugs targeting metabolic pathways also faces challenges. These issues constitute the current research hotspots and difficulties in this field.

### 6.1 The Role of Nucleotide Metabolism in Neural Development and Function

ATP, as the core molecule of cellular energy metabolism, provides direct power for neuronal activity through the hydrolysis of high‐energy phosphate bonds. In the central nervous system, processes such as the release of neurotransmitters from the presynaptic membrane, the operation of ion pumps during action potential conduction, vesicle recycling, and axoplasmic transport all rely on a continuous supply of ATP [570], and the dynamic balance of its intracellular concentration directly affects the functional state of neurons. In addition to its role as an energy carrier, ATP also acts as an important neurotransmitter/modulator involved in intercellular signal transmission. When neurons are stimulated, synaptic vesicles can release ATP along with classical neurotransmitters into the synaptic cleft, activating purinergic receptors on the postsynaptic membrane and regulating neuronal excitability. At nonsynaptic sites, ATP acts on distant target cells through volume transmission, participating in processes such as neuroinflammation, vascular regulation, and neural regeneration. This dual identity of ATP as both an “energy molecule” and a “signaling molecule” makes it a key hub connecting cellular metabolic status and neural functional activities.

Adenosine, as the main breakdown product of ATP, is generated in the extracellular space through a cascade of nucleotidease reactions, with its concentration being regulated by both cellular metabolic rates and the activity of membrane transporters [571]. As an endogenous neuromodulator, adenosine exerts a wide range of physiological effects by activating G protein‐coupled receptors (P1 receptor family). Under physiological conditions, adenosine regulates the firing frequency of neurons through a negative feedback mechanism: when neuronal activity increases, leading to higher ATP consumption, the extracellular concentration of adenosine rises, inhibiting excessive excitation to maintain the stability of neural networks [572]. Additionally, adenosine is involved in the regulation of the sleep–wake cycle, vasodilation, and the resolution of neuroinflammation. The dynamic balance of ATP and adenosine inside and outside the cell is precisely regulated by membrane transport proteins (such as ENTs and CNTs) and metabolic enzymes (such as CD39 and CD73). Once this balance is disrupted, it may contribute to the occurrence of neurological disorders such as epilepsy and chronic pain [573].

### 6.2 Molecular Mechanisms and Clinical Features of LNS

LNS is a rare X‐linked recessive genetic disorder characterized by a purine metabolism defect, caused by mutations in the HGPRT gene located at Xq26.2‐q26.3, resulting in complete loss of enzyme activity [574]. HGPRT plays a crucial role in the purine salvage synthesis pathway, responsible for catalyzing the conversion of hypoxanthine to IMP and guanine to GMP. This process not only recycles purine bases to conserve energy but also regulates de novo synthesis pathways through the consumption of PRPP via negative feedback [575]. When HGPRT activity is completely absent, the purine salvage synthesis pathway is blocked, leading to two key metabolic disturbances: on one hand, PRPP accumulates significantly in the cells due to ineffective utilization, strongly driving excessive de novo purine synthesis; on the other hand, hypoxanthine and guanine cannot be reused, resulting in their conversion to uric acid, causing plasma uric acid levels to rise dramatically, far exceeding normal levels [576]. The abnormal metabolism of uric acid leads to crystal deposition, which underlies the multisystem damage seen in LNS, manifesting as gouty arthritis, urinary stones, and renal damage [577].

However, the mechanisms underlying the occurrence of neurological symptoms are more complex and are currently believed to be closely related to the imbalance of the purine nucleotide pool and dysfunction of the brain's dopaminergic system. HGPRT is highly expressed in brain regions such as the basal ganglia, and its deficiency leads to insufficient synthesis of ATP and GTP in the synaptic vesicles of striatal dopaminergic neurons, affecting dopamine storage and release. Simultaneously, the accumulation of PRPP may interfere with neurotransmitter synthesis by allosterically activating other metabolic enzymes [578]. The typical neurological symptoms in patients gradually manifest after infancy, including severe extrapyramidal movement disorders (such as dystonia and choreoathetosis), progressive intellectual disability, speech development delays, and characteristic self‐injurious behaviors such as lip biting and finger biting. These self‐injurious behaviors are compulsive and uncontrollable, serving as an important clinical marker distinguishing LNS from other purine metabolism disorders, with their pathological mechanism potentially related to abnormalities in the central nervous system's reward and punishment pathways and pain perception disorders [579].

LNS is inherited in an X‐linked recessive manner, so almost all patients are male, while female carriers typically show no obvious symptoms but can pass the mutated gene to their offspring. Diagnosis of the disease primarily relies on typical clinical phenotypes, elevated plasma uric acid levels, and measurement of HGPRT enzyme activity (significantly reduced or absent enzyme activity in red blood cells or fibroblasts), with genetic sequencing clarifying the type of mutation. Although medications such as allopurinol can control hyperuricemia, there are currently no effective treatments for neurological symptoms [580]. Patients often die in adolescence due to severe movement disorders, swallowing difficulties, or infections, making early genetic diagnosis and genetic counseling crucial for disease prevention [581].

### 6.3 The Mechanism of Nucleotide Metabolism Regulation in Neurodegenerative Diseases

Alzheimer's disease (AD) represents the prototypical trajectory of neurodegenerative cognitive erosion, standing as the predominant etiology of dementia in the aging population. Currently, this debilitating syndrome afflicts over 50 million individuals globally—a figure projected to triple by 2050 as demographic shifts toward an aging society accelerate [582]. This clinical decline reflects a profound structural and functional disintegration within the central nervous system, where the progressive loss of cortical volume and the compensatory expansion of cerebral ventricles parallel the stealthy accumulation of amyloid‐beta (Aβ) proteotoxicity and Tau‐mediated neurofibrillary tangles [583]. While historical research has focused heavily on these protein aggregates, modern translational insights suggest that such hallmarks are merely symptoms of a deeper metabolic failure. It is now increasingly evident that the collapse of neural networks is fundamentally coupled to a systemic breakdown in brain energy homeostatic circuits and the subsequent dysregulation of nucleotide‐dependent signaling [584].

**FIGURE 6:**
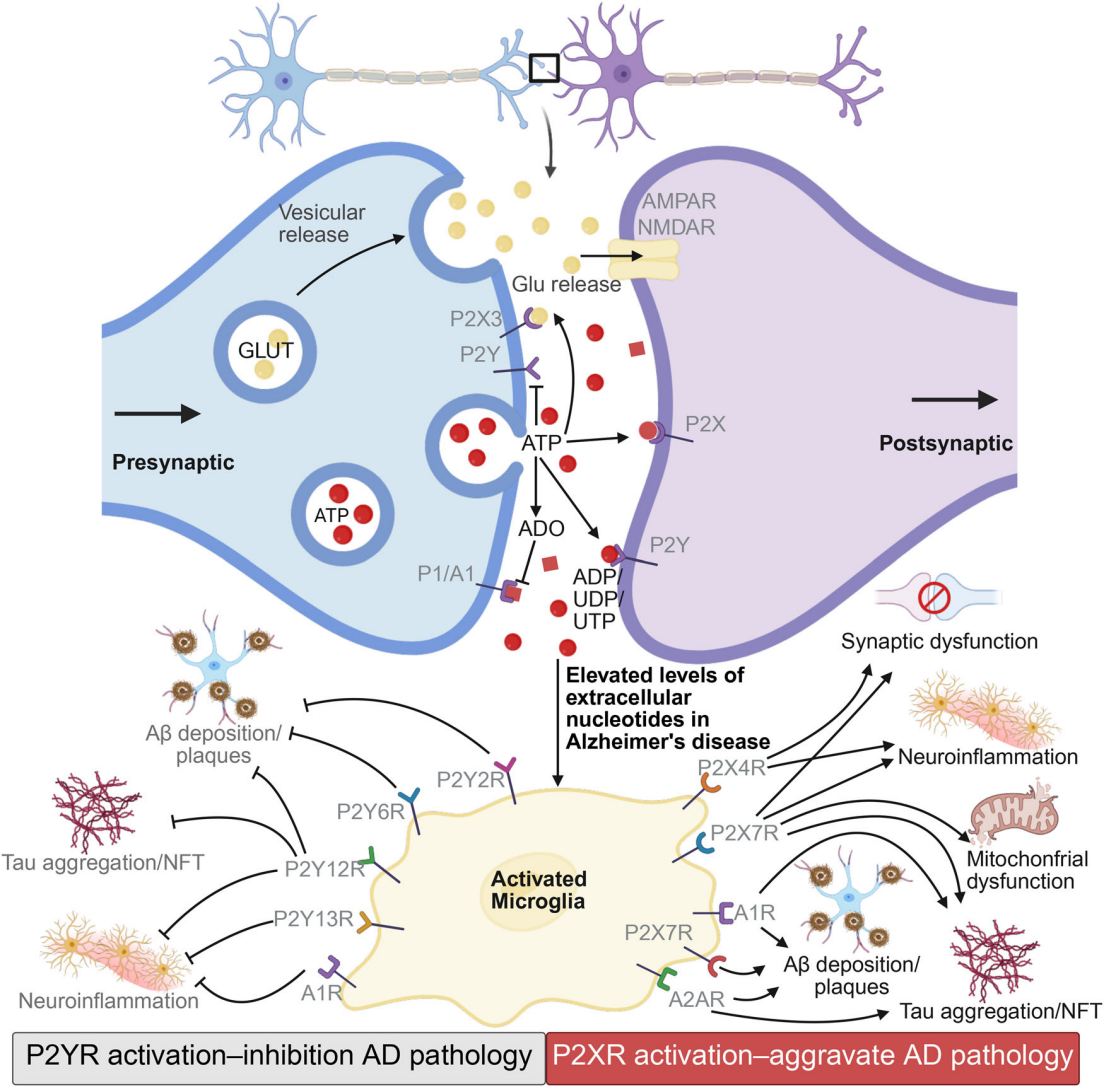
The dual role of purinergic signaling in the pathogenesis of Alzheimer's disease. ATP released from presynaptic terminals activates both P2X and P2Y receptors on neurons and microglia. Activation of P2Y receptors (P2YR) on neurons inhibits AD pathology by promoting synaptic function and attenuating neuroinflammation via the P2Y12R–P2Y13R–A1R pathway, which is also involved in regulating tau phosphorylation and neurofibrillary tangle formation. Conversely, activation of P2X receptors (P2XR), particularly P2×7R, exacerbates AD pathology by inducing microglial activation, leading to increased production of proinflammatory cytokines such as IL‐1β and IL‐18. This activation further triggers mitochondrial dysfunction, elevates extracellular nucleotide levels, and aggravates tau aggregation and Aβ deposition, collectively promoting neuroinflammation and synaptic dysfunction. The balance between P2YR and P2XR activation is critical for regulating neuroinflammation and synaptic integrity, offering potential therapeutic targets for AD management. Created with BioRender.com.

A defining shift in AD progression involves the functional reassignment of ATP from a requisite intracellular energy carrier to a potent extracellular damage‐associated molecular pattern [585]. As Figure 6, Aβ‐induced stress triggers the aberrant opening of astrocytic Connexin 43 hemichannels, facilitating the noncanonical efflux of ATP into the interstitium [586]. This eATP pool functions as a bidirectional molecular switch in the neuroinflammatory milieu. Specifically, the activation of metabotropic P2Y receptors such as P2Y2 and P2Y12 exerts neuroprotective effects by promoting microglial chemotaxis and Aβ phagocytosis. In stark contrast, the hyperactivation of ionotropic P2X receptors, most notably the P2×7 subtype, serves as a primary driver of disease exacerbation [585]. This P2×7‐mediated signaling recruits the NLRP3 inflammasome cascade, orchestrating the proteolytic maturation of IL‐1β and sustaining a self‐perpetuating state of chronic neuroinflammation that ultimately accelerates synaptic failure [587, 588].

Mechanistically upstream of these signaling aberrations, the bioenergetic residency of the neuron is governed by the flux of glucose through the PPP, which acts as the metabolic engine providing R5P for nucleotide biosynthesis [589]. In the AD brain, pervasive glucose hypometabolism—often conceptualized as “Type 3 diabetes”—severely restricts PPP flux, resulting in a critical scarcity of available R5P [584, 589]. This metabolic bottleneck cripples the neuron's capacity for both de novo synthesis and salvage‐based nucleotide repair, rendering the neuronal genome vulnerable to unrepaired oxidative DNA damage. Furthermore, the metabolic drift characterized by the depletion of NAD+ and the buildup of purine degradation products like uric acid destabilizes mitochondrial redox kinetics [590]. This thermodynamic instability precipitates the bioenergetic collapse of the tripartite synapse, where the failure of ATP‐dependent ion pumps leads to the loss of membrane potential and the ultimate termination of neurotransmission [591].

In synthesis, the orchestration of AD by nucleotide metabolism represents a multidimensional pathology wherein mitochondrial respiration, cytosolic biosynthesis, and extracellular signaling converge to drive disease progression. The transition from homeostatic nucleotide flux to pathological purinergic signaling—specifically, the ATP–P2×7–NLRP3 axis—marks a decisive tipping point in neurodegeneration [587] [PMID: 33889073]. By integrating the constraints of metabolic supply chains, such as PPP‐derived ribose synthesis, with downstream inflammatory and proteotoxic outcomes, it becomes evident that restoring nucleotide homeostasis offers a sophisticated therapeutic paradigm. Revitalizing these metabolic circuits may not only attenuate established proteinopathies but also fortify the structural and functional residency of the aging brain, providing a holistic strategy to preserve cognitive integrity.

### 6.4 Molecular Mechanisms and Clinical Features of ADA Deficiency

ADA is a rare autosomal recessive disease caused by ADA gene mutation [592]. The ADA gene located in the long arm of chromosome 20 (20q13.12) has pathogenic variation, resulting in ADA function defect or complete deletion [593]. Its core pathological feature is the toxic accumulation of metabolites caused by the blocking of purine metabolic pathway. When ADA activity is lost, adenosine and deoxyadenosine are largely retained in cells. The accumulation of deoxyadenosine is particularly significant in tissues highly dependent on purine metabolism, such as lymphocytes and nerve cells, because it can be phosphorylated by deoxycytidine kinase in cells to produce dATP, which has a strong inhibitory effect on ribonucleotide reductase, which is the rate limiting enzyme for deoxynucleotide production in DNA synthesis. Inhibition of its activity directly leads to cell division cycle arrest. At the same time, the blocked metabolism of adenosine leads to substrate shunting effect, prompting part of adenosine to produce amp through adenosine kinase catalysis, which is converted into imp by adenylate deaminase, resulting in the expansion of hypoxanthine metabolism pool.

Compensatory changes in the upstream and downstream enzymes constitute another feature of metabolic disorders. PNP, as the downstream key enzyme of ADA, can be upregulated in the absence of ADA. It attempts to maintain purine metabolism by accelerating the phosphorylation of inosine and deoxyinosine, but the compensatory effect has tissue specificity. The induced expression of PNP in brain tissue is significantly lower than that in peripheral blood lymphocytes. 5′‐nucleotidase (5′‐NT) showed two‐way regulation: the activity of extracellular 5′‐NT increased by 30–40%, promoted the hydrolysis of extracellular nucleotides to adenosine, and further increased the accumulation of extracellular adenosine [594]. The intracellular 5′‐NT activity decreased by about 25%, which may be related to the feedback inhibition of metabolites. This disorder of purine metabolic pathway forms a cascade effect, which develops from the initial loss of enzyme activity to the imbalance of the whole purine nucleoside metabolic network, laying the metabolic foundation for subsequent multiple system damage.

The abnormal accumulation of adenosine, deoxyadenosine, and their metabolic derivatives constitute the core pathological changes. Blood tests showed that the plasma adenosine concentration and deoxyadenosine level in typical patients were significantly increased, and the ratio of the two concentrations was positively correlated with the severity of the disease. The changes of metabolites in cerebrospinal fluid were more significant, and the concentration of adenosine was four to six times higher than that in peripheral blood, suggesting that the permeability of blood–brain barrier to these metabolites increased or there was local hypersynthesis in the central nervous system. In lymphoid tissues such as bone marrow and thymus, the accumulation level of deoxyadenosine is the highest, which can reach 15–20 times that of normal tissues, and directly affect the development microenvironment of hematopoietic cells and lymphocytes [595].

The abnormal activation of microglia is the initial link of neuroinflammatory response triggered by metabolite accumulation. In vitro experiments showed that deoxyadenosine could polarize microglia to proinflammatory M1 phenotype by activating TLR4/NF‐κ B signaling pathway, increase the proportion of CD16/32 positive cells, and increase the mRNA expression levels of proinflammatory cytokines TNF‐α, IL‐6, and IL‐1 β [596]. These inflammatory mediators form a cascade amplification effect through autocrine and paracrine, and the concentration of TNF‐α and IL‐6 in patients’ cerebrospinal fluid are increased, which directly induces neuronal apoptosis. Oxidative stress and inflammatory reaction promote each other and aggravate nerve injury together. Metabolite induced mitochondrial dysfunction increased ROS production, increased the level of 8‐OHdG and malondialdehyde in cerebrospinal fluid, and decreased the activities of superoxide dismutase and glutathione peroxidase, resulting in the imbalance of oxidation antioxidant system. This oxidative stress not only directly damages biological macromolecules such as lipids, proteins and nucleic acids, but also further amplifies the inflammatory response by activating MAPK and Nrf2 signaling pathways.

Astrocytes play a key regulatory role in the persistence of neuroinflammation [597]. In the absence of ADA, the uptake of glutamate by astrocytes decreased, resulting in the increase of glutamate concentration in synaptic space, which led to NMDA receptor‐mediated excitotoxicity. At the same time, its ability to synthesize neurotrophic factors (such as BDNF and GDNF decreased, weakening the protective effect on neurons. More importantly, activated astrocytes continuously recruit peripheral immune cells to infiltrate the central nervous system by secreting chemokines such as CXCL10 and CCL2, forming a chronic neuroinflammatory microenvironment. This cascade reaction of “metabolite accumulation inflammatory activation oxidative stress” eventually leads to the progressive loss of neurons and the destruction of neural circuits, which is also an important reason for the continuous progress of nerve injury after the correction of immune function in some patients [598].

As one of the most severe types of primary immunodeficiency diseases, patients face the risk of recurrent severe infections from birth due to developmental disorders of T cells, B cells, and natural killer cells. Untreated individuals often die in infancy. At the same time, about 30% of patients will experience neurological developmental abnormalities, manifested as intellectual disabilities, decreased motor function, seizures and other neurological complications. Some patients may even have irreversible neurological damage after immune function correction. In depth analysis of the metabolic disorder mechanism of ADA deficiency and its damage pathway to the nervous system can not only provide a theoretical basis for developing targeted treatment strategies, but also reveal the intrinsic relationship between purine metabolism, neural development, and immune regulation, and provide a new perspective for understanding the complex pathological network of multi system genetic diseases. Current research mainly focuses on the mechanisms and treatments of immune deficiencies, and there are still limitations in understanding how metabolic disorders affect neural function across systems, especially the lack of dynamic analysis of metabolic neural interactions during disease progression, which limits the development of neuroprotective interventions.

### 6.5 Nucleotide Metabolism Disorder in Cerebral Ischemia/Stroke

During cerebral ischemia/stroke, a sudden reduction in local cerebral blood flow leads to a rapid collapse of neuronal energy metabolism, triggering a series of cascade injury responses related to nucleotide metabolism [599]. Ischemia and hypoxia obstruct mitochondrial oxidative phosphorylation, sharply reducing ATP synthesis and causing failure of the sodium‐potassium pump in cell membranes, which leads to cellular depolarization and massive release of glutamate [600]. Simultaneously, a large number of neurons in the ischemic area undergo necrosis due to energy depletion, causing high concentrations of intracellular ATP to flood into the extracellular space through damaged cell membranes, raising eATP concentrations from physiological levels (1–10 µM) to millimolar levels within minutes. This abnormally elevated ATP acts as a potent damage signal, primarily mediating neuroexcitotoxicity through the activation of P2×7 receptors [601]. The P2×7 receptor is a nonselective cation channel expressed in microglia, astrocytes, and neurons. Overactivation of the P2×7 receptor forms a persistent calcium ion influx channel, leading to intracellular calcium overload [602]. In neurons, calcium overload directly activates calcium‐dependent proteases and apoptotic pathways, triggering cytoskeletal degradation and DNA fragmentation; in microglia, calcium signaling promotes the maturation and release of proinflammatory factors IL‐1β and IL‐18 through the activation of the NLRP3 inflammasome, recruiting more inflammatory cells to infiltrate the ischemic area. Furthermore, P2×7 receptor activation can also trigger the formation of membrane pores [603], leading to the release of proapoptotic factors and pyroptosis, further amplifying the inflammatory response and tissue damage. This “ATP–P2×7 receptor–inflammasome” axis constitutes the core mechanism for the secondary expansion of ischemic brain injury [604].

Astrocytes play a dual role in the purine metabolism disorder following ischemia [605]. On one hand, astrocytes gradually hydrolyze excess ATP into ADP, AMP, and eventually adenosine through high expression of CD39 and CD73, with adenosine exerting neuroprotective effects by activating A1 receptors (such as inhibiting glutamate release); on the other hand, severe ischemia can lead to dysfunction of astrocytes, reducing CD39/CD73 enzyme activity, which lowers ATP hydrolysis efficiency, while also releasing ATP to participate in the amplification of inflammation. This functional imbalance further exacerbates the disorder of extracellular purinergic signaling, becoming an important factor in the progression of the ischemic penumbra [606].

Intervention strategies targeting nucleotide metabolism disorders provide new directions for the treatment of ischemic stroke. P2×7 receptor antagonists (such as BBG, A‐438079) can significantly reduce cerebral infarct volume, inhibit neuroinflammation, and improve neurological functional outcomes in animal models, with mechanisms involving the blockade of calcium overload and inflammasome activation [607]. Exogenous administration of adenosine A1 receptor agonists can reduce ischemic glutamate release, but cardiovascular side effects limit their clinical application. In recent years, therapeutic strategies targeting the CD39/CD73 enzyme system have gained attention, as recombinant CD39 proteins [608] or CD73 activators can accelerate the degradation of ATP to adenosine, demonstrating effects in experimental models that alleviate blood–brain barrier disruption and neuroinflammation [609]. These studies suggest that regulating nucleotide metabolism disorders may become an effective means of neuroprotection during the acute phase of ischemic stroke, holding significant translational medical value.

Nucleotide metabolism serves as a core hub connecting cellular energy supply, genetic information transmission, and signal transduction, playing an irreplaceable role in the development, functional maintenance, and pathological processes of the nervous system. The purinergic signaling system participates in key physiological processes such as synaptic transmission, glial‐neuronal dialogue, and neuroinflammation regulation through the dual role of ATP and the neuromodulatory function of adenosine. The P2X/P2Y receptor family mediates rapid ionic signaling and slow metabolic regulation, while the P1 receptor family fine‐tunes neuronal excitability, sleep–wake cycles, and motor control through the specialization of subtypes like A1 and A2A. Various metabolic disorders caused by hereditary enzyme deficiencies directly lead to neuronal structural damage and functional impairment by disrupting nucleotide pool homeostasis or purinergic signaling. In neurodegenerative diseases, insufficient ATP synthesis due to mitochondrial dysfunction is an early event driving neuronal degeneration, while the imbalance of purine metabolites accelerates disease progression by affecting neurotransmitter release, protein homeostasis, and inflammatory responses. These findings reveal the central role of nucleotide metabolism abnormalities as a common pathological basis for various neurological diseases, with molecular mechanisms involving the accumulation of metabolic intermediates, disruption of signaling pathways, and collapse of energy supply in a multidimensional interplay.

Current research still faces many challenges, such as the spatiotemporal dynamics of the nucleotide metabolism network and its regulation of neuronal function, which have yet to be fully elucidated. For instance, the specific response mechanisms of different brain regions and cell types to metabolic disturbances require further investigation. Additionally, in clinical translation, the development of drugs targeting purinergic receptors faces issues of subtype selectivity, such as balancing the central penetration efficiency and peripheral side effects of A2A receptor antagonists, as well as the efficiency of NAD+ precursors' conversion in the brain and the assessment of long‐term safety. Furthermore, the limitations of the blood–brain barrier on the delivery of nucleotide‐based drugs and the compensatory mechanisms of metabolic pathways affecting therapeutic efficacy are bottlenecks that need to be overcome in the field. Future research could further focus on revealing the metabolic characteristics of specific neural circuits or subcellular structures, providing possibilities for discovering disease‐specific metabolic fingerprints. It could also explore the crossinteraction mechanisms between nucleotide metabolism and neuroimmunity, as well as epigenetic regulation, such as the regulatory effects of purine metabolites on microglial polarization phenotypes or the epigenetic regulatory mechanisms of NAD+‐dependent deacetylases in neural regeneration. Addressing these issues may pave the way for the development of personalized metabolic intervention treatment strategies, formulating precise remedial plans based on patient metabolic profiling (e.g., deoxynucleotide combination therapy for mitochondrial DNA depletion syndrome). Targeted delivery of gene editing technologies holds promise for curing hereditary enzyme deficiencies, while nanoparticle‐mediated blood–brain barrier penetration technologies will enhance the central delivery efficiency of nucleotide‐based drugs. Moreover, developing multitarget drugs that combine metabolic regulation and neuroprotective functions, such as dual‐target inhibitors that simultaneously regulate P2×7 receptors and the NLRP3 inflammasome, may provide more effective treatment options for complex neurological diseases. Breakthroughs in these cutting‐edge directions will advance nucleotide metabolism from basic research to clinical translation, opening new avenues for the diagnosis and treatment of neurological diseases.

## 7 The Mechanism and Treatment Strategy of Nucleotide Metabolism Disorder in Radiation‐Related Diseases

**FIGURE 7:**
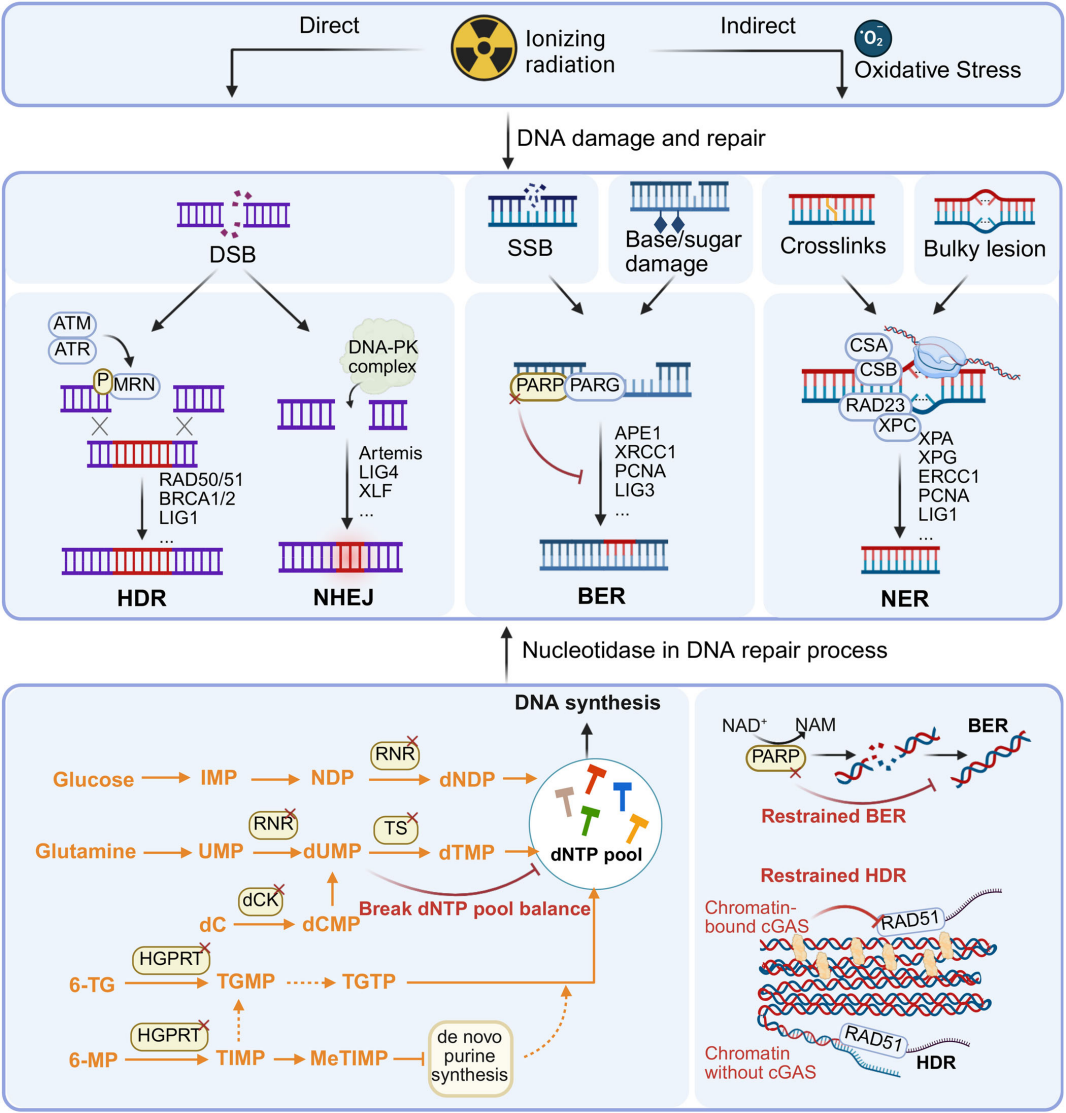
Regulation of DNA damage and repair by nucleotide metabolizing enzymes in response to ionizing radiation. This figure illustrates the various types of DNA damage caused by ionizing radiation and their corresponding repair pathways. It highlights the critical roles of nucleotide‐metabolizing enzymes such as ribonucleotide reductase (RNR), thymidylate synthase (TS), and poly(ADP‐ribose) polymerase 1 (PARP1) in modulating DNA repair processes, which significantly impact the efficacy of radiotherapy in cancer cells. Created with BioRender.com.

Radiation‐related diseases are a series of pathophysiological changes triggered by exposure to ionizing radiation, including acute radiation syndrome, chronic radiation injuries, and radiation‐induced tumors [610]. According to statistics from the World Health Organization, the incidence of radiation‐related diseases due to medical exposure, occupational exposure, and nuclear accidents is on the rise globally [611]. Complications such as radiation pneumonitis, radiation enteritis, and bone marrow hematopoietic dysfunction severely affect patients’ quality of life. Research indicates that radiation damage can disrupt nucleotide metabolism networks by directly damaging DNA molecular structures or indirectly inducing oxidative stress [612], which in turn affects the efficiency of DNA damage repair, creating a vicious cycle of “damage–metabolic disorder–secondary damage” [613]. Meanwhile, radiation therapy, as an important method in the comprehensive treatment of malignant tumors, is widely used in the clinical treatment of solid tumors such as lung cancer, nasopharyngeal cancer, and cervical cancer, with approximately 60% of cancer patients requiring radiation therapy during their disease course [614]. However, the radiation sensitivity of normal tissues limits the increase in radiation doses. Balancing the enhancement of tumor‐killing effects while protecting normal tissues has always been a core challenge in the field of radiation oncology. Therefore, clarifying the molecular mechanisms of DNA damage caused by radiation and the dual regulatory role of nucleotide metabolism in radiation damage will help develop metabolic intervention‐based treatment strategies for radiation‐related diseases (Figure 7).

### 7.1 The Nature of Radiation Damage: DNA Damage and Oxidative Stress

DNA damage by ionizing radiation mainly occurs through two interrelated mechanisms. First, the direct damage mechanism is embodied in the direct deposition of radiation energy [615]. When ionizing radiation such as X‐ray and γ‐ray penetrates biological tissues, part of the energy is directly transferred to DNA molecules, resulting in phosphodiester bond breakage, base structure damage or deoxyribose damage. This kind of damage is highly site‐specific and mainly occurs in the phosphate skeleton and base sites of the DNA double helix, resulting in direct single‐ or double‐strand breaks. However, in the water rich cell environment, the more important way is indirect effect. The core process is that the radiation energy is absorbed by the water molecules in the cells, which leads to the radiolysis of water and the production of a large number of reactive oxygen species such as hydroxyl radicals with high activity. These free radicals then spread and attack DNA, causing a series of complex chemical damage by seizing hydrogen atoms or adding them to bases [616]. Therefore, the indirect effect is essentially a key bridge for radiation‐induced oxidative stress to damage DNA.

Radiation induced oxidative stress is a continuous and possibly self‐amplified process. After the initial radiolysis of water produces a large number of reactive oxygen species, a series of secondary reactions will be triggered [617]. These active substances will damage mitochondria, leading to dysfunctional mitochondria to produce more endogenous ROS during respiration, forming a malignant positive feedback cycle [618]. At the same time, radiation can also activate NADPH oxidase and other enzymes on the cell membrane, making it continue to produce reactive oxygen species. In addition, the inflammatory reaction triggered by damaged cells will recruit immune cells, which will release more oxidative substances in an explosive manner during the clearance process, resulting in damage to adjacent cells that are not directly irradiated. This phenomenon is called “bystander effect.” Finally, continuous oxidative attack will exhaust the antioxidant defense system in cells, making cells more vulnerable to oxidative damage.

After DNA damage and oxidative stress, cells will activate a series of complex response networks, and the outcome depends on the severity of damage and repair efficiency [619]. If the damage can be perceived but cannot be repaired, cells usually start programmed apoptosis, which is an important self‐protection mechanism, especially in sensitive cells such as lymphocytes. If exposed to extremely high doses of radiation, cells may undergo uncontrolled necrosis and cause inflammation. Some cells may enter an irreversible aging state, survive but stop proliferation, and secrete harmful factors to affect the tissue microenvironment [620]. More dangerous is that if DNA damage is not faithfully repaired, it may lead to gene mutation, chromosome aberration, and induce malignant transformation of cells [621].

### 7.2 The Role of Nucleotide Metabolism in Radiation Injury

The core of cell damage caused by ionizing radiation is DNA breakage, in which nucleotide metabolism plays a dynamic dual role, and its state directly determines whether the cell fate is toward repair or collapse. In terms of protective effect, radiation injury activates the cellular stress signal network, regulates the expression of key enzymes in the nucleotide rescue synthesis pathway through transcription and translation levels, and provides sufficient dNTPs for DNA repair. In the purine salvage pathway, HGPRT catalyzes the conversion of hypoxanthine and guanine to IMP and GMP, and APRT specifically recovers adenine to produce AMP. At 3–6 h after radiation exposure, the mRNA levels of HGPRT and APRT were upregulated two to three times, and the binding sites of NF‐κ B and p53 in the promoter region were activated, which could enhance the enzyme activity by enhancing the transcription efficiency [622]. In pyrimidine salvage system, TK1 and TK2 play roles in S phase and mitochondria, respectively. TK1 provides raw materials for nuclear DNA repair by phosphorylating thymidine to dTMP [623]; TK2 kept the dTTP pool of mitochondrial DNA stable. This provides sufficient “raw materials” for high fidelity DNA repair pathways (such as source recombination), supports the accurate repair of damaged DNA, and helps cells avoid secondary genomic instability caused by lack of replication raw materials.

However, when the damage is too heavy or the metabolism is unbalanced, the disorder of nucleotide metabolism amplifies oxidative stress through multi node abnormalities, forming a cascade effect of DNA secondary damage. The results showed that the dNTP pool increased five to six times after 30 min of exposure to ionizing radiation, and the imbalance of dNTP library led to the wrong incorporation of DNA polymerase in the repair process, which significantly increased the mutation rate [624]. Sustained damage may also deplete dNTP, forcing cells to adopt error prone repair mechanisms and trigger chromosome rearrangement [625]. At the same time, the reactive oxygen species produced by radiation will directly attack the nucleotide metabolic enzymes and substrates, resulting in oxidative damage of dNTP precursor, and its incorporation into DNA will directly introduce mutation. The energy crisis caused by mitochondrial damage will further weaken the ability of nucleotide synthesis, form a vicious cycle of repair failure and damage accumulation, and ultimately promote cell death or malignant transformation [626]. Understanding this dual mechanism provides a key idea for clinical practice: in radiation protection, repair can be enhanced by maintaining metabolic homeostasis. In tumor radiotherapy, drugs can be designed to inhibit the nucleotide metabolism of tumor cells and selectively aggravate the repair crisis to achieve sensitization.

In addition, the relationship between radiation therapy dose/fractionation regimen and nucleotide metabolism adaptation has become an important frontier field in radiation biology and disease metabolism research. This relationship affects the efficacy of radiotherapy and the protection of normal tissues, and its core lies in the dynamic interaction between radiation‐induced DNA damage and nucleotide metabolism reprogramming by cells to repair the damage. Different radiotherapy regimens exert varying pressures on cells and microenvironments, leading to differentiated metabolic adaptations [627]. First, low‐dose/multiple conventional fractionated radiotherapy induces sustained metabolic stress leading to sublethal accumulation of DNA damage, activating sustained DNA damage response signaling pathways (such as ATM/ATR, PARP) [628]. In this case, in order to maintain sustained repair ability, cells (including tumor cells and proliferating normal cells) will adaptively upregulate key enzymes involved in de novo and salvage synthesis pathways of nucleotides, such as RNR and TK1 [629], whose expression or activity may increase. Therefore, cells establish a “metabolic adaptation” state, and the dNTP pool is maintained or even expanded to support multiple rounds of repair. This may lead to enhanced repair ability of tumor cells, which is one of the potential mechanisms of radiotherapy resistance. Large‐scale and complex DNA damage caused by high‐dose radiotherapy: not only does it generate a large number of DSBs, but it also forms more complex and difficult to repair DNA damage clusters [630]. The rapid increase in cell repair demand may lead to the rapid depletion of local or intracellular dNTP pools. If synthesis cannot keep up with consumption, dNTP depletion will become the limiting factor for repair failure. High dose radiation can directly or indirectly damage metabolic enzymes or disrupt their co factors, inhibiting nucleotide synthesis ability. High doses can seriously damage tumor vascular endothelial cells, leading to further hypoxia, acidosis, and nutrient deficiency in the TME, limiting the overall supply of nucleotide precursor substances, thus initiating an irreversible pathway of tumor cell death [631].

Current research suggests that low‐dose hypersensitivity effects (HRS) are associated with cellular adaptive responses [632]. Extremely low doses of irradiation (<0.5 Gy) can sometimes cause hypersensitivity, and preadministering extremely low doses may induce resistance to subsequent higher doses by activating protective mechanisms, including metabolic adjustments [633]. This is related to the preadaptation of early nucleotide metabolism and repair ability [634]. Besides, the tumor antigens and danger signals released by radiotherapy activate the immune system. The strong proliferation and effector function of T cells are also highly dependent on nucleotide metabolism. The competition between tumor cells and infiltrating immune cells for nucleotide precursors (such as glucose and glutamine) has become a key factor affecting the distant effects of radiotherapy and the efficacy of combined immunotherapy.

A deep understanding of the dynamic relationship between radiation therapy dose/dose regimen and nucleotide metabolism adaptation not only helps to reveal the biological basis of differences in radiation therapy efficacy, but more importantly, provides strong scientific basis for individualized radiation therapy design and rational combination therapy, which is a key link in achieving precise tumor radiation therapy.

### 7.3 Treatment Strategies for Radiation‐Related Diseases Based on Nucleotide Metabolism

#### 7.3.1 Regulation of Nucleotide Metabolism and Enhancement of Radiation Resistance in Normal Tissues

Exogenous supplement or endogenous regulation can optimize the DNA damage repair ability of normal cells, which can enhance the radiation resistance of normal tissues and reduce the side effects of radiotherapy. As the basic raw material for DNA synthesis and repair, the metabolic state of nucleotides directly determines the efficiency and fidelity of DNA repair. The first is the exogenous supplement of nucleosides or nucleotide precursors, such as adenosine, cytidine, and other mixtures, so that normal cells can rapidly supplement dNTP pool after radiation through an efficient remedial synthesis pathway, providing sufficient raw materials for DNA repair. Preclinical studies have proved that it has a clear protective effect on intestinal tract, bone marrow, and other tissues. On the other hand, drugs can regulate endogenous key metabolic enzymes to tilt the cell state, such as activating AMPK pathway (such as metformin) to promote nucleotide synthesis or inhibiting dNTP degrading enzyme SMAHD1, so as to enhance the production capacity of repair raw materials of the cells [635]. In addition, regulating related signaling pathways and cofactors is also an important strategy, such as reducing radiation‐induced harmful inflammatory response by inhibiting cGAS–STING pathway [636] or supplementing nicotinamide and folic acid to maintain PARP activity and one carbon metabolism, so as to support the repair process. The key to the success of this strategy is to take advantage of the inherent differences in metabolism between tumor cells and normal cells: many tumor cells are overly dependent on the dysfunctional “de novo synthesis” pathway, while normal tissues are better at using the “remedial synthesis” pathway of exogenous nucleosides. In the acute repair period after radiotherapy, timely nutritional supplementation can make the normal tissue dominant in the repair. In clinical practice, this strategy can also be used in combination with radiosensitizers (such as gemcitabine) to protect normal tissues by selectively supplementing specific nucleosides (such as deoxycytidine), without weakening or even enhancing the killing effect on tumors, so as to broaden the treatment window [637]. The future development will depend on the accurate monitoring of metabonomics and the progress of nano delivery technology to achieve the ultimate goal of “providing the right repair materials for the right cells at the right time,” and ultimately improve the quality of life of patients while improving the curative effect of radiotherapy.

#### 7.3.2 Radiosensitization Strategy for Tumors

PROTAC is a bifunctional molecule that binds to the target enzyme at one end and recruits E3 ubiquitin ligase at the other end, thereby labeling the target enzyme with ubiquitin chain and guiding it to be degraded by proteasome. The core of the strategy of enzyme degradation agent based on PROTAC in radiotherapy sensitization is to selectively degrade the key DNA damage repair enzymes or regulatory proteins in tumor cells by using PROTAC molecules, so as to produce synergistic lethal effect with radiotherapy [638]. Radiation induced DNA double‐strand breaks and other damages, while PROTAC systematically degraded the key enzymes responsible for repairing these damages, such as BRCA1/2 and RAD51 in the source recombination repair pathway, DNA‐PKCs in the nonhomologous end connection, and regulatory proteins PARP1 and ATR/CHK1, which changed the damage from “repairable” to “irreparable,” and eventually led to cell death [639]. Compared with traditional small molecule inhibitors, PROTAC has unique advantages in completely eliminating the function of target proteins, overcoming drug resistance caused by mutation or overexpression and targeting “nondrug” proteins by virtue of its catalytic degradation and event driven characteristics [640]. In general, this strategy provides a new way with great potential for reversing tumor radiotherapy resistance.

As mentioned above, MTHFD2 and MTH1 play a key role in the response of tumor cells to oxidative stress and metabolic reprogramming. Using its selective inhibitors to enhance the sensitivity of tumor to radiotherapy has become a promising combination therapy strategy. Its core is to reduce the ability of tumor to resist radiotherapy by targeting the metabolic fragility of tumor cells and the mechanism of genome stability. Radiotherapy kills cells by inducing DNA damage, but tumor cells can survive through enhanced DNA repair, antioxidant defense, and abnormal metabolism. MTHFD2 inhibitors limit the source of DNA repair materials by exhausting the nucleotide pool and can also reduce the level of NADPH, thereby weakening the antioxidant capacity of tumors. Therefore, they aggravate the ROS damage caused by radiotherapy and interfere with energy metabolism [641], making tumor cells unable to effectively repair the damage after radiotherapy. Preclinical studies have confirmed its sensitization effect in non‐small cell lung cancer and other models [642]. As a nucleotide pool cleaning enzyme, MTH1 can hydrolyze oxidized dNTP and prevent its incorporation into DNA [643]. Due to the high level of oxidative stress, the survival of cancer cells is highly dependent on MTH1 [644]. MTH1 inhibitors actively introduce “lethal mutations” and additional DNA breaks by allowing oxidative damaged nucleotides to be incorporated into new DNA and have synergistic effects with primary radiation damage [645]. At the same time, DNA containing oxidized bases will also interfere with the function of ATM, PARP, and other repair proteins, further hindering damage repair. This strategy has the advantage of selective killing to MTH1 dependent cancer cells [646]. In the future, the combination of MTHFD2 and MTH1 inhibitors may synergistically destroy the stability of tumor genome from the two sides of “raw material supply” and “quality control,” which has excellent antitumor potential.

In addition, nanomaterials as multifunctional platforms can enhance the sensitivity of tumors to radiation through various mechanisms. First, by utilizing the EPR effect of nanoparticles, traditional radiosensitizers or DNA repair inhibitors can be efficiently and targetedly delivered to the tumor site, improving therapeutic efficacy and reducing systemic toxicity [647]; second, using nanoparticles containing high atomic number elements such as gold and hafnium can locally amplify energy deposition under radiation, generating more secondary electrons and free radicals, directly enhancing the killing effect [648]. Meanwhile, nanomaterials are designed to regulate the TME or synergistically activate antitumor immunity to regulate the TME, fundamentally improving radiation resistance [649, 650].

At present, the research of radiation‐related diseases based on nucleotide metabolism faces multiple challenges, and the lack of target specificity is the core bottleneck restricting the clinical transformation. Most nucleotide metabolizing enzymes (such as RRM2 and TK1) are expressed in normal proliferating cells and tumor cells. Traditional small molecule inhibitors often cause toxic reactions of “no distinction between ourselves and foe.” For example, gemcitabine can inhibit tumor RRM2 while causing dNTP synthesis disorder in bone marrow hematopoietic stem cells [651]. The complexity of metabolic network aggravates the difficulty of intervention. Purine and pyrimidine metabolism are interconnected through PPP, one carbon unit metabolism, and other pathways. A single target intervention is easy to trigger compensatory metabolic reprogramming. In the process of clinical transformation, it is necessary to further improve the drug delivery system and dose optimization system. Nucleotide analogues have strong hydrophilicity and low membrane permeability and are easy to be rapidly removed after intravenous administration. Their plasma half‐life is usually less than 30 min, which is difficult to maintain the effective concentration. Oral preparations face the intestinal first pass effect, and the difference in bioavailability can reach 30–70%. Dose optimization lacks dynamic monitoring means. Currently, it relies on fixed body weight dose scheme and ignores individual metabolic baseline differences. Future research can break through the existing limitations with the help of single‐cell metabonomics technology. The metabolic characteristics of different cell subsets after radiation injury were analyzed by mass spectrometry flow cytometry, which can identify the specific nucleotide metabolic phenotype of tumor stem cells with radiation resistance, and guide the individualized target selection. In addition, the artificial intelligence algorithm constructs a metabolic radiosensitivity prediction model by integrating multi omics data, which can realize dynamic dose adjustment and maximize the treatment gain ratio. The crossfusion of these frontier directions is expected to promote the clinical application of nucleotide metabolism targeting strategy in the treatment of radiation‐related diseases.

#### 7.3.3 Combination Therapy Strategy: Synergistic Application of Nucleotide Metabolism Regulators and Radiotherapy

To effectively radio sensitize a variety of BRCA1/2‐mutated cancers, including breast cancer, prostate cancer, and pancreatic cancer, clinicians have devised a therapeutic regimen combining ionizing radiation with PARP inhibitors [652, 653, 654]. A clinical trial for triple‐negative breast cancer demonstrated that, in the context of BRCA1/2 mutations, radiotherapy combined with PARP inhibitors can indirectly increase the frequency of unrepaired DSBs through the BER pathway, enhancing radiotherapy efficacy with good safety and tolerability [655]. Studies have found that PARP inhibitors, such as olaparib and niraparib, can block DNA damage repair in ovarian cancer cells [656, 657], making them more sensitive to radiotherapy [658, 659]. PARP‐1‐targeted radiotherapy is effective in mouse GBM models, with I‐PARPi‐treated tumor‐bearing mice exhibiting longer survival times compared with controls [660]. Cellular experiments demonstrate that the PARP‐1/2 inhibitor MK‐4827 sensitizes human breast tumor xenografts to ionizing radiation [661]. Further research suggests that nitric oxide [54] donors can inhibit homologous recombination repair by blocking BRCA expression, and PARP inhibitors can provide new radiosensitization targets for BRCA1/2‐proficient patients through a cascade with NO donors [662]. These findings underscore PARP's crucial role in tumor RT tolerance and its potential for successful application in broader PARP‐targeted cancer therapies, leading to the development of novel combination therapies to overcome cancer drug resistance. Regulatory agencies have approved several PARP inhibitors over the last 10 years (Table 1), with each agent showing distinct yet overlapping clinical applications [663]. Olaparib has secured multiple indications over time, beginning with advanced ovarian cancer [664] in 2014 and broadening to include primary peritoneal, HER2‐negative breast, and metastatic castration‐resistant prostate cancers [664, 665, 666, 667]. Rucaparib followed a similar trajectory, initially approved for ovarian cancer in 2016 [668] and later for recurrent ovarian, primary peritoneal, and metastatic prostate cancers. Niraparib [151, 659] and talazoparib [669] have also received approvals for ovarian/peritoneal and HER2‐negative breast cancers, respectively. In parallel, veliparib (ABT‐888) [670] and fluzoparib remain under clinical evaluation; the latter was discovered in 2017 and is currently being tested in ovarian, lung, and breast cancer populations [671].

## 8 Clinical Treatment Strategy Based on Nucleotide Metabolism

Inhibition of nucleotide metabolic enzymes has emerged as a pivotal therapeutic strategy with expanding applications across oncology and immune‐mediated disorders (Table 1). The clinical landscape is anchored by poly(ADP‐ribose) polymerase inhibitors, among which olaparib, rucaparib, and niraparib have achieved Phase IV approval for the treatment of breast, ovarian, pancreatic, and prostate malignancies. Meanwhile, next‐generation agents including talazoparib and fluzoparib are currently advancing through Phase III trials for breast, prostate, and lung cancers, with veliparib similarly positioned in Phase III development for ovarian cancer, triple‐negative breast cancer, and non‐small cell lung cancer. These PARP inhibitors exploit synthetic lethality in DNA repair‐deficient tumors, representing a paradigm shift in precision oncology.

Classical antimetabolites remain foundational to contemporary chemotherapy regimens. Agents targeting ribonucleotide reductase, exemplified by gemcitabine, have attained Phase IV status for pancreatic, breast, bladder, ovarian, and lung cancers, while cytarabine serves as a mainstay for acute myeloid leukemia, acute lymphoblastic leukemia, and chronic myeloid leukemia. The TS inhibitor class encompasses capecitabine and 5‐fluorouracil, both Phase IV approved for gastrointestinal malignancies including colorectal, gastric, and pancreatic cancers, with additional applications in breast and cervical carcinomas. Pemetrexed, another established TS inhibitor, has achieved Phase IV approval for pleural mesothelioma and non‐small cell lung cancer. These agents continue to demonstrate therapeutic value through their disruption of de novo nucleotide synthesis essential for rapidly proliferating malignant cells.

Novel metabolic targets have recently entered clinical development. NAMPT inhibitors, specifically padnarsertib and daporinad, are undergoing Phase I–II evaluation for hematologic malignancies including acute myeloid and lymphoblastic leukemias, as well as selected solid tumors. The adenosine pathway has emerged as a particularly dynamic area of investigation, with antagonists targeting ectonucleotidases advancing through mid‐ and late‐stage trials. CD73 inhibitors oleclumab and quemliclustat have progressed to Phase III for non‐small cell lung, colorectal, and pancreatic cancers, while the antibody mupadolimab and IPH5301 are in Phase I–III development across diverse solid tumor indications. The CD39‐targeting landscape includes TTX‐030 in Phase II for pancreatic cancer and lymphoma, alongside IPH5201 and SRF617 in Phase I–II for melanoma, fibrosarcoma, and advanced solid tumors. These immunometabolic modulators function by relieving adenosine‐mediated immunosuppression within the TME, thereby potentiating antitumor immunity.

Beyond oncology, preclinical investigations have revealed substantial promise for modulators of the cGAS and the stimulator of IFN genes axis. STING inhibitors including C‐176, H‐151, nitro‐fatty acids, BB‐Cl‐amidine, palbociclib, SN‐011, and STING‐IN‐2 are currently in preclinical or experimental stages for SLE, Aicardi‐Goutières syndrome, STING‐associated vasculopathy with onset in infancy, COPA syndrome, amyotrophic lateral sclerosis, Parkinson's disease, and IBD. Concurrently, cGAS inhibitors demonstrate translational potential across inflammatory and neurological conditions. Suramin has advanced to Phase III for prostate cancer and plasma cell neoplasms, while oxychloroquine maintains Phase IV status for acute RA and lupus erythematosus. Emerging preclinical candidates perillaldehyde and PF‐06928215 specifically target aberrant cytosolic DNA sensing implicated in autoimmune pathogenesis.

Collectively, these advances underscore a broadening therapeutic landscape that extends from established cytotoxic applications in cancer to emerging indications in autoimmune and neurologic disorders. The diversification of target classes—from classical metabolic enzymes to innate immune sensors—affirms nucleotide metabolism as a versatile and continually expanding frontier for next‐generation drug development (Table 1).

## 9 Conclusion and Prospects

This review mainly elucidates the key regulatory role of nucleotide metabolism in tumor development, immune diseases, neurodegenerative diseases and other diseases. By delving into the metabolic pathways of various diseases, the importance of nucleotide metabolism as a therapeutic target has been highlighted. At the same time, we discussed enzymes such as PARP‐1, RNR, TS, G6PD, and MTH1 and gained a deeper understanding of how targeting these enzymes regulates cellular life activities, providing guidance for the development of therapeutic targets. In addition, this review emphasizes the importance of maintaining dNTP pool homeostasis for genome integrity and cellular adaptability and highlights the relationship between dNTP pool stability and genome integrity during disease processes.

Nucleotide metabolism constitutes a highly dynamic, compartmentalized core regulatory network intricately intertwined with cellular fate determination. The fundamental operations of life necessitate the maintenance of metabolic homeostasis. As demonstrated in the relevant literature, disruption to metabolic pathways has the potential to compromise multiple essential cellular functions, including substrate supply, energy balance, signal transduction, and genetic integrity. This disruption is a common feature in various pathological processes, including tumori, immune dysfunction, gout, neurological disorders, and radiation‐related diseases. It is anticipated that research in this field will undergo a transition, moving from the description of correlations to the identification of causal mechanisms and the development of precision intervention strategies. At the translational medicine level, high‐throughput metabolomics is being leveraged to discover novel biomarkers, such as specific nucleosides and related derivatives, as well as developing downstream allosteric regulators for key enzymes, tissue‐specific delivery drugs, and personalized therapies based on the principle of “synthetic lethality” is promising. Meanwhile, metabolic immunotherapy targeting the eATP–adenosine signaling pathway is becoming a new hotspot for reshaping the TME and treating autoimmune diseases. However, the research also faces numerous challenges, such as such as the spatiotemporal heterogeneity of nucleotide metabolism across cell types, tissues, organs, and subcellular compartments, compensatory pathways and resistance mechanisms due to metabolic plasticity, and the complex interactions of environmental factors like nutrition and gut microbiota. In the future, leveraging systems biology approaches such as single‐cell and spatial metabolomics, live‐cell imaging, and integrative computational models is expected to provide deeper insights into the fine regulation of this complex network.

In summary, through continuous exploration and interdisciplinary integration of nucleotide metabolism, innovative diagnostic tools and targeted therapies aimed at its key nodes are bound to emerge, ultimately achieving the fundamental goal of transitioning from understanding metabolism to precisely regulating metabolism to improve human health.

## Author Contributions

Xiaoying Zhao conceptualized the study and drafted the manuscript. Xiaoying Zhao, Yiyu Zeng, Shu Rao, Yike Xu, and Rui Liu contributed to editing and revising the manuscript. Mouna Khouchani and Hassna Soummane provided detailed suggestions during the article revision process. Rui Liu and Jingyi Li reviewed and finalized the manuscript. All authors have read and approved the final manuscript.

## Funding

This work was supported by West China School/Hospital of Stomatology Sichuan University (RD‐03‐202404; R.L.), Sichuan Provincial Department of Science and Technology (2025YFHZ0091; J.L.), the Scientific Research Program for Young Talents of China National Nuclear Corporation (CNNC202482; J.L.), the Science and Technology Bureau of Chengdu (2026‐YF05‐01289‐SN; J.L.), and the Open Project of Sichuan Clinical Research Center for Radiation and Therapy, The Second Affiliated Hospital of Chengdu Medical College, Nuclear Industry 416 Hospital (No. 2025ZDSYSY06; X.Z.)

## Ethics Statement

The authors have nothing to report.

## Conflicts of Interest

The authors declare no conflicts of interest.

## Data Availability

The authors have nothing to report.
